# Label-Free Quantitative Proteomics of Embryogenic and Non-Embryogenic Callus during Sugarcane Somatic Embryogenesis

**DOI:** 10.1371/journal.pone.0127803

**Published:** 2015-06-02

**Authors:** Angelo Schuabb Heringer, Tatiana Barroso, Amanda Ferreira Macedo, Claudete Santa-Catarina, Gustavo Henrique Martins Ferreira Souza, Eny Iochevet Segal Floh, Gonçalo Apolinário de Souza-Filho, Vanildo Silveira

**Affiliations:** 1 Laboratório de Biotecnologia, Centro de Biociências e Biotecnologia (CBB), Universidade Estadual do Norte Fluminense Darcy Ribeiro (UENF). Campos dos Goytacazes, RJ, Brazil; 2 Laboratório de Biologia Celular de Plantas, Instituto de Biociências, Universidade de São Paulo (USP), São Paulo, SP, Brazil; 3 Laboratório de Biologia Celular e Tecidual, CBB-UENF, Campos dos Goytacazes, RJ, Brazil; 4 Mass Spectrometry Research and Development Laboratory, Waters Corporation, São Paulo, SP, Brazil; Wuhan University, CHINA

## Abstract

The development of somatic cells in to embryogenic cells occurs in several stages and ends in somatic embryo formation, though most of these biochemical and molecular changes have yet to be elucidated. Somatic embryogenesis coupled with genetic transformation could be a biotechnological tool to improve potential crop yields potential in sugarcane cultivars. The objective of this study was to observe somatic embryo development and to identify differentially expressed proteins in embryogenic (E) and non-embryogenic (NE) callus during maturation treatment. E and NE callus were cultured on maturation culture medium supplemented with different concentrations (0.0, 0.75, 1.5 and 2.0 g L^-1^) of activated charcoal (AC). Somatic embryo formation and differential protein expression were evaluated at days 0 and 21 using shotgun proteomic analyses. Treatment with 1.5 g L^-1^ AC resulted in higher somatic embryo maturation rates (158 somatic embryos in 14 days) in E callus but has no effect in NE callus. A total of 752 co-expressed proteins were identified through the SUCEST (The Sugarcane EST Project), including many housekeeping proteins. E callus showed 65 exclusive proteins on day 0, including dehydrogenase, desiccation-related protein, callose synthase 1 and nitric oxide synthase. After 21 days on maturation treatment, 14 exclusive proteins were identified in E callus, including catalase and secreted protein. NE callus showed 23 exclusive proteins on day 0 and 10 exclusive proteins after 21 days on maturation treatment, including many proteins related to protein degradation. The induction of maturation leads to somatic embryo development, which likely depends on the expression of specific proteins throughout the process, as seen in E callus under maturation treatment. On the other hand, some exclusive proteins can also specifically prevent of somatic embryos development, as seen in the NE callus.

## Introduction

Sugarcane (*Saccharum* spp.) is an allogamous plant that belong to the Poaceae family and is the main source of sugar worldwide. Sugarcane is a significant component of the economy in more than 100 countries in tropical and subtropical regions [1]. Brazil plays an important role in the sugar industry being both the largest producer of this species [2] and the largest producer of sugar derived from sugarcane worldwide [3]. In addition, sugarcane has become an important bioenergy source and is classified among the most important energy crops for bioethanol production. Given its high potential to accumulate biomass, sugarcane lignocellulosic materials are candidates for the production of second-generation ethanol from cell wall hydrolysis [4]. Even though it has not yet proven economically viable, second-generation ethanol is highly desir able because it would enable to use of crop biomass fractions that are not animal feed or food for humans, for biofuel generation [5].

Sugarcane displays limited genetic diversity because most cultivated hybrids are derived from the interspecific hybridization of *Saccharum officinarum* and *Saccharum spontaneum* [6, 7]. Crop yield improvement is highly dependent on the incorporation of superior features, and with conventional breeding, this process occurs very slowly, taking 10–14 years to release a new variety [8]. Modern sugarcane breeding programs include biotechnological approaches, such as marker-assisted breeding, DNA mapping and genetic transformation [9]. Genetic transformation, which can introduce genes that encode desirable traits into elite sugarcane cultivars, provides an alternative method to improve pest and disease resistance, as well as yield. This method has already generated positive results, not only in terms of increased sugar content but also improved crop performance [10]. Improving sugarcane will require the development of an optimized tissue culture and plant regeneration system as a prerequisite for the production of genetically modified sugarcane plants. *In vitro* techniques for the mass propagation of sugarcane plantlets via somatic embryogenesis pathways are well established, but the production of highly nodular embryogenic (E) callus is a critical step in many ongoing efforts to improve the sugarcane germplasm through genetic transformation [9]. This pathway is preferred for the regeneration of plants that have been obtained from the genetic transformation of sugarcane using either particle bombardment or *Agrobacterium*-mediated transformation [10].

Somatic embryogenesis is analogous to zygotic embryogenesis, wherein a single cell or a small number of somatic cells are precursors to the formation of a somatic embryo [11]. This process is only possible with totipotent plant cells, which can undergo genetic reprogramming to differentiate into any cell type, thereby leading to the creation of an entire embryo from a group of cells or even a single somatic cell [12]. Sugarcane presents 2 types of callus: embryogenic (E) callus is smooth and compact, with the potential for somatic embryo formation, whereas non-embryogenic (NE) callus is friable or soft and translucent and lacks the potential for somatic embryos formation [13]. Microscopically, the E callus is formed by cells with embryogenic characteristics, such as a rounded shape, prominent nuclei, a high nucleus:cytoplasm ratio, small vacuoles and organized globular structures. However, the NE callus presents dispersed, elongated and vacuolated cells with a low nucleus:cytoplasm ratio and does not permit the development of somatic embryos even upon exposure to a maturation stimulus [13]. Compared with others tissue culture techniques, somatic embryogenesis offers advantages such as automated large-scale production and genetically stable regenerated plantlets, which are important in breeding programs, permitting the fixation of genetic gain by the capture of the additive and non-additive components of genetic variability [14, 15]. In addition, somatic embryogenesis permits the creation of cycling cultures through the use of cell suspensions or through secondary somatic embryogenesis, thereby permitting the large-scale commercial production of elite plants [15]. However, in spite of such advantages, the maturation and conversion phases for sugarcane somatic embryos still present a bottleneck against reaching the high-efficiency regenerative protocol.

During somatic embryo development, several biochemical and molecular processes occur that are important for understanding this morphogenetic route, though several of these processes have not been fully elucidated [16, 17]. The study of the physiological, biochemical and molecular aspects associated with the competence and determination of the E callus in sugarcane have the potential to identify specific markers (such as proteins), that could be used to monitor the development of somatic embryos in E and NE callus and/or genetically modified embryos. These studies could help in understanding induction and the acquisition of competence in E and NE callus to develop somatic embryos in this species. A better understanding of the phenomena related to somatic embryogenesis will permit the development of strategies for *in vitro* culture propagation and the genetic manipulation of plants [18].

Proteomic techniques can be used to develop specific proteomic maps for each stage of the somatic embryogenesis protocol, permitting the identification of specific differentially expressed proteins to serve as molecular markers for the acquisition of embryogenic competence and somatic embryo evolution during *in vitro* sugarcane morphogenesis. Through these proteomic maps, along with transgenesis technology, these markers can be strapolated and translated into practical applications in the field, which is a growing area known as translational plant proteomics [19]. The multidimensional protein identification technology “MudPIT” has become a popular approach for performing shotgun proteomics with high resolution orthogonal separation coupled to tandem mass spectrometry (2D-nanoLC-MS/MS) [20]. This technology has enabled the identification of low-abundant proteins [21], which are often missed when using two-dimensional electrophoresis [21]. Therefore, proteomics has evolved to focus on the functionality of the huge datasets that are acquired through a vast number of analytical technologies. The quality of the procedures, the orthogonality that is provided by MudPIT and the ion mobility [22–24] with increased selectivity and specificity have recently received great attention with regard to untargeted proteomics samples. Thus, modern acquisition techniques, such as a multiplex high-resolution format MS^E^ (multiplexed DIA—data-independent acquisition) [25] and high-definition HDMS^E^ (DIA with ion mobility) are valuable acquisitions that are required for shotgun proteomics and complex samples due to the overlapping chimeric peptide resolving power [26, 27]. This work is the first study to compare of E and NE callus under maturation treatment using 2D-nanoESI-HDMS^E^ technologies in sugarcane.

The present study determined the effects of maturation treatments on morphology and proteomics, as well as the relationship between maturation treatment and somatic embryogenesis competence and evolution in sugarcane cv. SP80-3280. In this sense, proteomics analyses were used to investigate differences in the acquisition of competence and somatic embryo development between E and NE sugarcane callus. In this work proteins were identified using the HDMSE (data independent acquisition with ion mobility) technique, which permits the identification of large number of protein in complex samples, being a qualitative and quantitative analysis. In total, 752 co-expressed proteins were identified, along with 65 unique proteins in the E callus at the end of the maturation treatment.

## Materials and Methods

### Plant material

E and NE callus from the sugarcane cv. SP80-3280 were indeced as previously described [13]. Briefly, immature nodal segments with axillary buds were collected at Universidade Federal Rural do Rio de Janeiro (UFRRJ), located at Campos dos Goytacazes, Rio de Janeiro, Brazil, where the species is abundant, with previous permission from the researcher (Dr. Carlos Frederico de Menezes Veiga). This species is not considered threatened. The nodal segments were then planted in plastic trays containing the commercial substrate PlantMax (DDL Agroindustria, Paulínia, Sao Paulo, Brazil). The trays were maintained under ambient conditions for approximately 3 months. During this time, new plants originated from sprouting axillary buds and grew approximately 45 cm. After removal of the mature leaves, the shoot apical meristems were used as explants for callus induction. The shoot apical meristems were surface sterilized in 70% ethanol for 1 min, immersed in 50% commercial bleach (hypochlorite from 1 to 1.25%) for 30 min and subsequently washed 3 times with sterilized water in a laminar flow chamber. Subsequently, explants were transversely sectioned into 2-mm-thick slices and inoculated in assay tubes (150 × 25 mm) containing 10 mL of MS [28] (Phytotechnology Lab, Overland Park, KS, USA) culture medium supplemented with 20 g.L^-1^ sucrose, 2 g.L^-1^ Phytagel (Sigma-Aldrich, St. Louis, MO, USA) and 10 μM 2,4-dichlorophenoxyacetic acid (2,4-D) (Sigma-Aldrich). The culture medium pH was adjusted to 5.8 prior to autoclaving for 15 min at 1.5 atm and 121°C. The explants were maintained in a growth room at 25°C ± 2°C in the dark.

After approximately 45 days, the induced callus were transferred to Petri dishes (90 × 15 mm) containing 20 mL of the same culture medium. The callus was subcultured every 21 days and separated into 2 types, E and NE, as previously described [13]. Smooth and compact callus were classified as E, whereas friable and soft callus ware classified as NE.

### Maturation treatment

For the maturation treatment, three colonies containing 200 mg of fresh mass (FM) obtained from E and NE callus were inoculated in Petri dishes containing 20 mL of MS culture medium supplemented with 20 g.L^-1^ sucrose, 2 g.L^-1^ Phytagel, and varying concentrations of activated charcoal (AC; Sigma-Aldrich; 0.0, 0.75, 1.5 and 3.0 g.L^-1^). The pH of the culture medium was adjusted to 5.8 prior to the addition of Phytagel (Sigma-Aldrich) was added. The culture medium was sterilized by autoclaving at 121°C for 15 min. After inoculation, the cultures were maintained in a growth chamber at 25 ± 1°C under dark conditions for the first 7 days. Thereafter, photoperiods of 16 h light (60 μmol.m^2^.s^-1^) were used for up to 28 days of culture.

E callus competence was analyzed at 0 (before incubation on maturation treatment), 7, 14, 21 and 28 days of incubation by monitoring the FM (the initial FM was 200 mg FM per colony) and the number of somatic embryos produced.

For both types of callus, three separate Petri dishes, containing three colonies each, were examined per treatment at each sampling time.

Samples of both callus types were collected at 0 and 21days and stored at -20°C for later proteomic analysis.

### Proteomic analyses

#### Total protein extraction

Only the treatment that gave yielded the greatest number of somatic embryos (MS medium supplemented with 1.5 g.L^-1^ AC) was used for proteomic analysis. E and NE callus at the beginning of the experiment (day 0, before incubation with the maturation treatments) and after 21 days of culture ware select for analysis and marked as E-0, E-21, NE-0, and NE-21. These time points were chosen because day 0 corresponds to cultures that are still in the dark, whereas day 21 corresponds to one week prior to the end of the maturation treatment in the light, thus yielding embryos at advanced stages of development, since on day 28 somatic embryos were already converting to plantlets.

Protein extracts were prepared in biological triplicate (500 mg FM) that as previously described [29]. The samples were ground and transferred to clear, 2-mL microtubes containing 1.5 mL of extraction buffer containing 7 M urea (GE Healthcare, Freiburg, Germany), 2 M thiourea (GE Healthcare), 1% DTT (GE Healthcare), 2% triton X-100 (GE Healthcare), 0.5% pharmalyte (GE Healthcare), 1 mM PMSF (Sigma-Aldrich), and 5 μM pepstatin (Sigma-Aldrich). The extracts were briefly vortexed and kept in extraction buffer on ice for 30 min followed by centrifugation at 12,000 g for 5 min at 4°C. The supernatants were transferred to clear microtubes, and the proteins were precipitated on ice for 30 min in 10% trichloroacetic acid (Sigma-Aldrich) and washed three times with cold acetone (Merck, Darmstadt, Germany). Finally, the proteins were re-suspended and concentrated in 0.5 mL of the same extraction buffer. The protein concentration was estimated using the 2-D Quant Kit (GE Healthcare) using bovine serum albumin (BSA, GE Healthcare) as a standard, and the samples were stored at −20°C until proteomic analyses.

#### Protein digestion

The three resulting protein extracts from each treatment were pooled according to Luge et al. (2014) [30], totaling 1000 μg, in order to evaluate the biological variance between treatments in the discovery proteomics approach. This pool was solubilized in 50 mM NH_4_HCO_3_ pH 8.5 and then centrifuged at 4000 g for 5 min at 8°C. Then, 50 μL (100 μg) of the supernatant was pipetted onto a VivaSpin membrane (GE Healthcare), to which 400 μL of 50 mM NH_4_HCO_3,_ pH 8.5, was added and followed by centrifugation at 4000 g for 10 min. at 8°C. This last step was repeated at least 2 more times for protein concentration and clean-up. Finally, 50 μL was left on the membrane, collected and used for digestion [31].

For trypsin digestion, a 2 μg.μL^-1^ solution of 50 μL of the previous sample plus 25 μL of 0.2% v/v *Rapi*GEST (Waters, USA) [32] was added to a 1.5-mL microfuge tube, vortexed for 5 sec and heated in an Eppendorf Thermomixer Comfort device at 80°C for 15 min. Then, 2.5 μL of 100 mM dithiothreitol (DTT) was added and placed in the thermomixer at 60°C for 30 min. The tubes were placed on ice (30 sec), and 2.5 μL of 300 mM iodoacetamide (IAA) was added, followed by vortexing for 5 sec and incubation in the dark for 30 min at room temperature. Then, 20 μL of trypsin (50 ng. μL^-1^) solution that was prepared with 50 mM NH_4_HCO_3_ pH 8.5 was added (Promega^,^ USA) and placed in a thermomixer at 37°C overnight, after which 10 μL of trifluoroacetic acid (TFA) 5% v/v was added to precipitate the surfactant *Rapi*GEST SF, vortexed for 5 sec, incubated at 37°C for 90 min (without shaking) and centrifuged at 4000 × g for 30 min at 8°C. Then, 100 μL of the supernatant was collected and transferred to the Total Recovery Vial (Waters, USA) for further shotgun mobility-DIA proteomics analysis.

#### Mass spectrometry analysis

Qualitative and quantitative bidimensional nanoUPLC tandem nanoESI-HDMS^E^ (multiplexed DIA—data-independent acquisition) experiments were conducted using both a 1-h reverse-phase gradient from 7% to 40% (v/v) acetonitrile (0.1% v/v formic acid) and a 500 nL.min^-1^ nanoACQUITY UPLC 2D Technology system. A nanoACQUITY UPLC HSS T3 1.8 μm, 75 μm × 15 cm column (pH 3) was used in conjunction with a reverses-phase (RP) XBridge BEH130 C18 5 μm, 300 μm × 50 mm nanoflow column (pH 10). Typical on-column sample loads were 500 ng of total protein digests for each of the 5 fractions (500 ng per fraction/load). For every measurement, the mass spectrometer was operated in resolution mode with a typical *m/z* resolving power of at least 35000 FWHM and an ion mobility cell that was filled with nitrogen gas and a cross-section resolving power at least 40 Ω/ΔΩ. The effective resolution with the conjoined ion mobility was 1,800,000 FWHM. Analyses were performed using nano-electrospray ionization in positive ion mode nanoESI (+) and a NanoLockSpray (Waters, Manchester, UK) ionization source. The lock mass channel was sampled every 30 sec. The mass spectrometer was calibrated with an MS/MS spectrum of [Glu1]-Fibrinopeptide B human (Glu-Fib) solution (100 fmol.μL^-1^) that was delivered through the reference sprayer of the NanoLockSpray source. [M + 2H]^2+^ = 785.8426) was used for initial single-point calibration, and MS/MS fragment ions of Glu-Fib were used to obtain the final instrument calibration. DIA scanning with added specificity and selectivity of a non-linear ‘T-wave’ ion mobility (HDMS^E^) device [33] was performed with a SYNAPT G2-S HDMS mass spectrometer (Waters, Manchester, UK), which was automatically planned to switch between standard MS (3 eV) and elevated collision energies HDMS^E^ (19–45 eV) applied to the transfer ‘T-wave’ CID (collision-induced dissociation) cell with argon gas; the trap collision cell was adjusted to 1 eV, using a millisecond scan time that was previously adjusted based on the linear velocity of the chromatographic peak that was delivered through nanoACQUITY UPLC to generate a minimum of 20 scan points for each single peak, both in low-energy and high-energy transmission at an orthogonal acceleration time-of-flight (*oa*-TOF) and a mass range from *m/z* 50 to 2000. The RF offset (MS profile) was adjusted such that the nanoESI-HDMS^E^ data were effectively acquired from *m/z* 400 to 2000, ensuring that any masses that were observed in the high-energy spectra with less than *m/z* 400 arose from dissociations in the collision cell. The samples and conditions were injected with the same amount on the column. Stoichiometric measurements based on scouting runs of the integrated total ion account (TIC) prior to analysis were performed to ensure standardized molar values across all conditions.

## Results

### Maturation and morphogenetic changes

**Fig 1:**
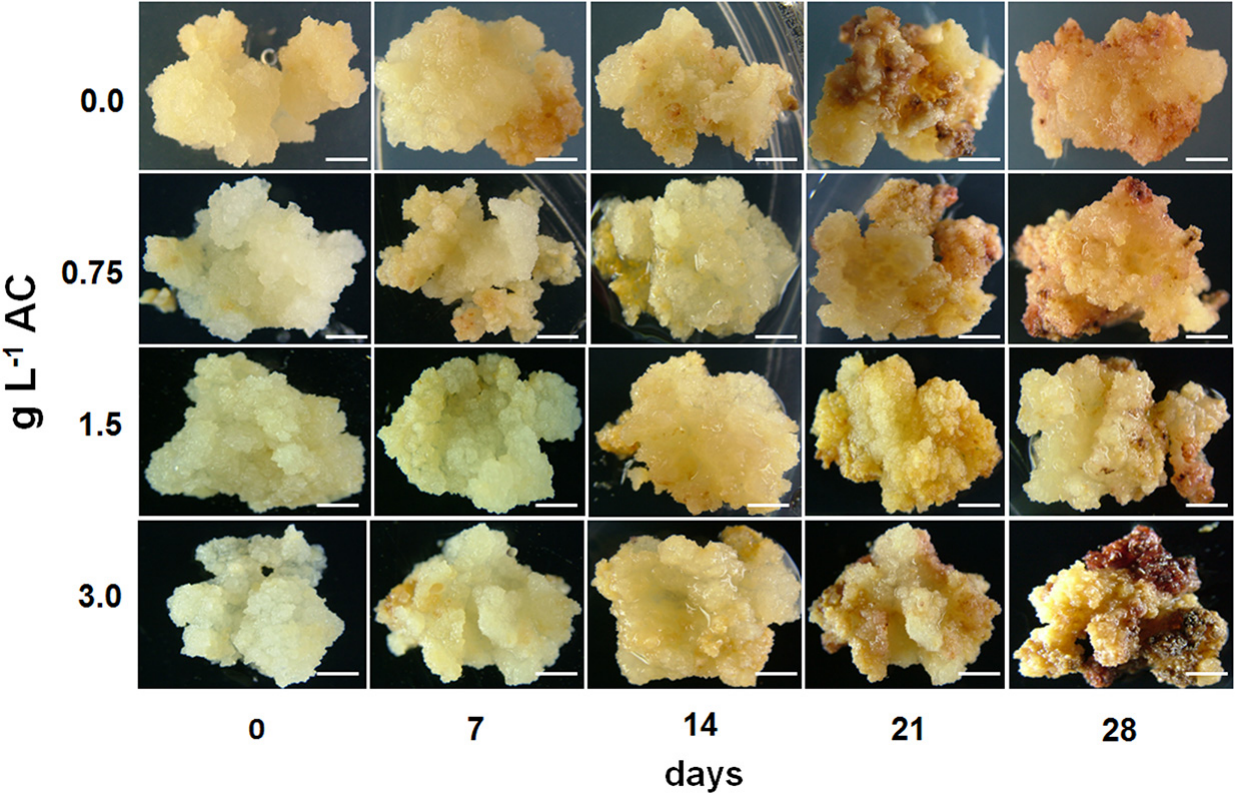
Morphological response of non-embryogenic callus during maturation treatment. Non-embryogenic (NE) callus subjected to maturation treatment in the presence of different concentrations (0.0; 0.75; 1.5 and 3 g L^-1^) of activated charcoal (AC). Bars = 5 mm.

**Fig 2:**
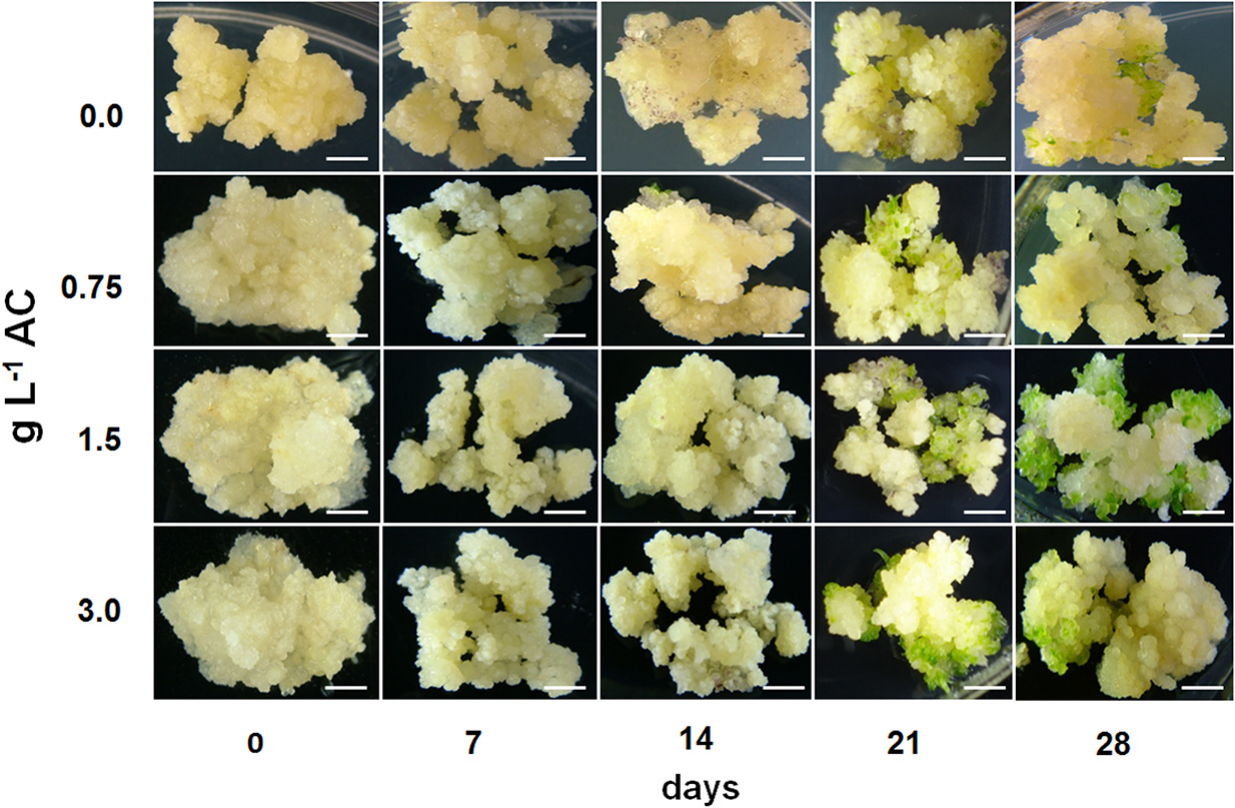
Morphological response of embryogenic callus during maturation treatment. Embryogenic (E) callus weresubjected to maturation treatment in the presence of different concentrations (0.0; 0.75; 1.5 and 3 g L^-1^) of activated charcoal (AC). Bars = 5 mm.

Maturation treatment were unable to promote somatic embryo differentiation in the NE callus at the end of 28 days of culture (Fig 1). In contrast, when E callus containing a pro-embryogenic mass (S1 Fig) were removed from the growth regulators and supplemented with AC, they were able to develop somatic embryos, permitting evolution to advanced stages of development during the 28 days of incubation and generating large numbers of healthy plantlets at the end of the process (Fig 2). Comparing the treatments, the response were observed on MS culture medium supplemented with 1.5 g.L^-1^ AC, with a high number of somatic embryos (158) already reached on the 14^th^ day of culture, and a total of 160 somatic embryos at the end of 28 days of culture (Table 1 and Fig 2). The treatment with 0.75 g.L^-1^ AC also showed a large number of somatic embryos at the end of the process (181 somatic embryos), but this treatment proved to be slightly delayed compared with the best treatment, generating a smaller number of somatic embryos (114) at 14 days (Table 1 and Fig 2). MS culture medium without AC (control) also produced somatic embryos but to a significantly lower degree than the other two treatments described above (Table 1 and Fig 2). AC cleary increase maturation and the number of somatic embryos that are formed. However, providing this compound in excess can also be harmful to somatic embryo formation, as observed with 3 g.L^-1^ AC, which led to a low number of somatic embryos (74) at the end of the process (Table 1 and Fig 2).

**Table 1:** Effect of activated charcoal (AC) on the number of somatic embryos that formed in sugarcane embryogenic (E) callus (200 mg FM) under different maturation treatments.

Time of culture (days)	AC (g L^-1^)
	0.0	0.75	1.5	3.0
	Somatic embryo number			
0	0*	0	0	0
7	0	0	0	0
14	12 ± 3,61	114 ± 3,61	158 ± 11,93	29 ± 1,53
21	97 ± 7,94	120 ± 15,72	120 ± 11,59	129 ± 15,31
28	110 ± 15,70	181 ± 17,50	160 ± 11,68	74 ± 11,68

*Mean ± standard deviation, n = 4.

### Protein identification during maturation treatment

The protein identification and quantitative data processing were performed using dedicated algorithms [25] and by searching against a database using the default parameters for ion accounting and quantitation [34, 35]. The databases utilized were reversed “on-the fly” during the database queries and appended to the original database to assess the false-positive identification rate. For proper spectra processing and database searching conditions, the ProteinLynx Global Server (PLGS) v2.5.2 software package with Apex3D, Peptide 3D, and Ion Accounting informatics (Waters, UK) was used. The SUCEST protein databank (http://sucest-fun.org/) containing specific annotations for sugar cane was utilized. The search conditions were based on taxonomy (*Saccharum spp*.); up to 1 maximum missed cleavages by trypsin allowed; variable modifications by carbamidomethyl (C), acetyl N-terminal, and oxidation (M); and a default maximum false discovery rate (FDR) value of 4%. The obtained proteins were organized by the software algorithm into a statistically significant list corresponding to increased and decreased regulation ratios between the different groups. Normalizations were performed automatically by Expression^E^ software which was included inside PLGS informatics (Waters, UK) using the recommended default parameters. Co-expressed proteins were filtered based on a fold change of log_2_ 1.2, as determined by the overall coefficient of variance for all quantified proteins across all replicates, and classified as up-regulated when log_2_ is 1.2 or greater and as down regulated when log_2_ is -1.2 or less.

After searching the SUCEST project database (http://sucest-fun.org/), 1267 proteins were identified with an average of 15 peptides per protein and were filtered only when found across all replicates. From these proteins, 752 were present in all four samples (S1 Table), and 403 were common across all of the conditions, as identified in two of three type of samples (S2 Table). From the total identified proteins, 65 were unique to the E callus at the beginning (E-0), with 14 proteins that were unique at 21 days of maturation treatment (E-21) (Table 2). The NE callus showed 23 unique proteins at the beginning (NE-0) and 10 unique proteins at 21 days of the maturation treatment (NE-21) (Table 2).

**Table 2:** Unique proteins that were identified in embryogenic (E) or non-embryogenic (NE) sugarcane callus under maturation treatment.

SUCEST accession number	Description	Peptide count	Peptides used for quantitation	Confidence score	Counts
**Unique E-0 Proteins**					
>SCVPCL6041D12	udp-sugar pyrophosphorylase-like	14	2	74.88	507.73
>SCJFRZ2009B04	s-adenosylmethionine:2-demethylmenaquinone. . .	12	3	74.35	1252.10
>SCRFLB1054G06	charged multivesicular body protein 5	10	3	63.70	3159.40
>SCRLSB1041H10	chorion family 2 expressed	9	2	52.40	46.50
>SCEZLB1007H06	meiotic nuclear division protein 1 homolog	8	1	43.04	495.37
>SCBFAD1089G07	akin gamma	7	1	41.21	1282.14
>SCACAM1071C11	coiled-coil protein	8	1	39.56	1466.22
>SCQSRT1035C03	peptidylprolyl cis-trans isomerase	7	3	39.09	736.50
>SCCCRZ1001D08	tubulin—tyrosine ligase-like protein 12	7	1	36.03	328.78
>SCQSAM1033A03	u4 tri-snrnp-associated 65 kda protein	7	3	34.98	579.64
>SCCCCL3120E07	mtn19-like protein	5	4	34.31	5131.92
>SCBGLR1098A04	lsm sm-like protein family member	3	2	28.31	1286.06
>SCQGLB1030C05	phosphoprotein phosphatase pp7	5	2	25.56	39.68
>SCEQRT1027A06	3-deoxy-d-arabino heptulosonate-7-phosphate synthase	5	1	24.48	78.72
>SCEPLR1051B05	chromdomain-containing protein crd101	5	2	24.40	450.84
>SCQGLR1062D09	methylosome subunit picln-like	4	1	22.27	1118.28
>SCQGSB1143E08	polyphosphoinositide phosphatase-like isoform 1	3	1	21.48	1624.85
>SCCCCL4002E09	monosaccharide transport protein mst1	2	1	20.80	9.31
>SCCCLB1025C07	deoxyribonuclease tatd	4	1	20.63	94.61
>SCQGRT1042A06	amp binding protein	4	1	19.80	89.38
>SCJLLR1054E12	ncase_orysj ame: full = neutral ceramidase short = n-cdase. . .	3	3	19.17	556.98
>SCCCLR1001H11	cosa_orysj ame: full = costars family protein	3	1	18.77	3920.17
>SCCCLR2002H10	ring-box protein	2	2	17.69	3728.50
>SCEPLR1008D03	had-superfamily subfamily iia	3	2	17.34	102.14
>SCRFLR2038B03	gata transcription factor 25	3	2	16.94	1444.23
>SCCCST2003E11	pheophorbide a oxygenase	2	1	14.51	1202.84
>SCACLR1036F06	hydroxymethylbutenyl 4-diphosphate synthase	3	1	14.14	249.50
>SCSFRT2070B09	carbohydrate transporter sugar porter transporter	3	1	13.68	14.26
>SCCCLR1068F03	ru1c1_sorbi ame: full = u1 small nuclear ribonucleoprotein. . .	1	1	13.24	80.59
>SCBGLR1047G06	cation transport protein chac	3	1	12.99	298.91
>SCCCLB2005F01	long form-like	2	1	11.23	260.34
>SCEZST3147G06	bcplh protein	2	1	10.84	776.66
>SCRULB1060G07	prli-interacting factor l-like	2	1	10.83	2170.47
>SCQGHR1012E03	nitric oxide synthase interacting protein	2	1	10.80	425.00
>SCSBHR1051A12	burp domain-containing protein	2	1	10.79	813.98
>SCCCCL4006E10	glycerophosphoryl diester phosphodiesterase-like	2	1	10.65	679.69
>SCBGSB1025C10	2 coiled coil domains of eukaryotic origin (kd)-like protein	2	1	10.55	1808.32
>SCUTRZ3073E01	carboxylic ester hydrolase	2	1	10.44	821.02
>SCSFAD1108H10	callose synthase 1 catalytic subunit	2	1	10.16	265.93
>SCAGLR1021B10	phosphoribosylanthranilate transferase	2	1	9.81	156.17
>SCAGLR2033F09	folylpolyglutamate synthase	2	2	9.77	158.84
>SCQSST3116H01	drought-inducible protein 1os	2	1	9.71	155.50
>SCCCCL4007E09	brca1 c terminus domain containing expressed	2	1	9.34	844.00
>SCQSST1036B01	adenylyl-sulfate kinase	2	1	8.92	64.13
>SCRFAM1026C02	dehydrogenase-like protein	2	1	8.68	129.72
>SCBFSD2038B09	gibberellin-regulated protein 2 precursor	1	1	8.27	570.52
>SCVPLR1028B03	hua enhancer 2	1	1	6.71	1482.94
>SCEQLB1068H03	methylglutaconyl- methylglutaconyl- hydratase	1	1	6.41	81.74
>SCEPCL6019C12	transferring glycosyl groups	1	1	5.83	27.80
>SCQSFL3032A08	Os03g0157700 [*Oryza sativa* Japonica Group]	1	1	5.60	72.20
>SCEPCL6029B05	af466199_12gb protein	1	1	5.60	208.87
>SCSBFL5016D03	sec14-like protein 1	1	1	5.26	25.97
>SCRLRZ3115E11	thimet oligopeptidase-like	1	1	5.17	422.23
>SCCCLB1C03F07	cslc7_orysj ame: full = probable xyloglucan glycosyltransferase. . .	1	1	5.11	757.22
>SCSGRZ3061F09	upf0414 transmembrane protein c20orf30 homolog	1	1	5.07	316.79
>SCCCRT1004A07	root-specific protein rcc3	1	1	5.03	350.96
>SCRLFL3005C03	speckle-type poz protein	1	1	4.90	679.77
>SCBFSD1037G05	desiccation-related protein pcc13-62 expressed	1	1	4.84	499.12
>SCJFRT2059A12	hipl1 protein expressed	1	1	4.49	124.63
>SCCCLB1023H09	200 kda antigen p200-like protein	1	1	4.21	283.81
>SCCCCL1002F10.b	cmp-kdo synthetase	1	1	4.16	482.69
>SCJFLR1013D06	zn—- containing protein	1	1	3.99	344.94
>SCJLLR1104A12	abivp1 transcription factor	1	1	3.99	631.91
>SCVPRZ2044C07	copper-transporting p-type atpase	1	1	3.73	24.30
>SCQSHR1020G11	lecithine-cholesterol acyltransferase-like 4-like	1	1	3.72	254.95
**Unique E-21 Proteins**					
>SCCCCL3120D10.b	catalase- expressed	5	1	46.01	247.02
>SCQSAM1033H01	disulfide oxidoreductase electron carrier oxidoreductase	7	1	40.30	176.62
>SCSGRZ3060B04	b-keto acyl reductase	3	1	23.03	401.66
>SCSGST1070D03	putative kinesin [*Oryza sativa* Japonica Group]	4	1	22.74	35.63
>SCSBSD2032F10	secreted protein	2	1	20.51	2497.09
>SCAGLR1064D03	wd40 repeat-containing protein smu1-like	3	2	15.79	592.08
>SCQGRT1045A06	tho complex subunit 4	3	2	14.61	526.94
>SCEQAM2038G11	craniofacial development protein	2	1	9.73	122.92
>SCCCRZ1002A07	transformation transcription domain-associated protein	2	1	9.70	301.41
>SCAGLB1071A03	ac079853_1 myosin heavy chain-like	2	1	8.66	220.75
>SCCCCL3080D01	arp9_orysi ame: full = actin-related protein 9	1	1	5.20	145.22
>SCJFSB1010B12	tgacg-sequence-specific dna-binding protein tga—like	1	1	4.97	651.41
>SCSBFL4011C12	Os07g0646200 [*Oryza sativa* Japonica Group]	1	1	4.32	299.86
>SCRFAM1027C11	ac079874_16 dna binding protein	1	1	4.04	346.24
**Unique NE-0 Proteins**					
>SCJFRZ2028D05	esterase d	6	2	38.40	202.16
>SCCCRZ3001A02	smc n terminal domain containing expressed	4	2	23.50	985.08
>SCVPRT2075D04	af061282_24 patatin-like protein	3	1	20.92	1306.42
>SCBFRT1071A02	4cll4_orysj ame: full = 4-coumarate—ligase-like 4	4	3	20.43	144.22
>SCBGRT1046F12	pleckstrin homology domain-containing protein 1	4	2	20.32	199.51
>SCJLFL1047A09	hox1b protein	3	1	19.41	4845.25
>SCQGST1032A06	myb-like dna-binding domain containing protein	2	1	18.26	324.54
>SCEPRZ1010A01	fasciclin domain	2	1	18.11	194.33
>SCCCRZ2C04F12	l-allo-threonine aldolase	3	1	15.03	205.83
>SCQGLR1041H02	primary amine oxidase-like	3	1	14.38	78.77
>SCBGST3106D08	isoflavone reductase irl	3	1	13.67	204.58
>SCRUSB1078F12	cmv 1a interacting protein 1	1	1	11.01	6097.33
>SCAGLB1069B07	helix-loop-helix dna-binding domain containing expressed	2	2	10.54	1992.79
>SCRFLB1053G11	mgdg synthase type a	2	2	10.14	5580.03
>SCAGLR2011C06	ring zinc finger protein	2	1	10.00	2380.70
>SCCCCL4001E08	pyridoxal biosynthesis protein pdx2-like	1	1	9.53	63.64
>SCCCCL4007B04	Os04g0129900 [*Oryza sativa* Japonica Group]	2	2	8.88	341.06
>SCMCRT2103F09	hmngt_sorbi ame: full = cyanohydrin beta-glucosyltransferase. . .	1	1	6.01	1475.92
>SCMCST1053E03	af114171_7tnp2-like protein	1	1	5.44	2735.70
>SCCCCL1001D11.b	oxoglutarate dehydrogenase (succinyl-transferring) e1. . .	1	1	5.43	521.40
>SCCCCL3005D09.b	tyrosine specific protein phosphatase family protein	1	1	5.19	50.02
>SCSGAD1006E05	cyt-p450 monooxygenase	1	1	4.72	392.38
>SCACLR1130H08	dl related protein	1	1	4.66	68.53
**Unique NE-21 Proteins**					
>SCRLLV1026A01.b	polyubiquitin-like protein	14	1	141.40	247.11
>SCEQLB1063H08	nicotinate-nucleotide pyrophosphorylase family protein	7	2	40.67	13721.30
>SCJLRT1018B07	nicotianamine aminotransferase a-like	4	2	21.43	1618.19
>SCEQRT1024G07	alkaline neutral invertase	3	1	18.70	154.00
>SCVPLR2027H10	pleckstriny domain-containing expressed	3	2	13.35	2332.11
>SCBFLR1083H12	bacterial-induced peroxidase precursor	1	1	12.70	713.41
>SCQSFL3038G02.b	Os12g0534000 [*Oryza sativa* Japonica Group]	2	1	9.44	2677.01
>SCCCCL4005F07	mitosis protein dim1	1	1	5.40	5183.42
>SCJFRT1012E10	loc100136880 isoform 1	1	1	5.34	8092.31
>SCSGRZ3062C02	af466200_17 galactosyltransferase Family	1	1	4.60	5144.71

Confidence scores were calculated by ProteinLynx Global Server (PLGS).

### Protein functional classification during maturation treatment

Functional classifications based on protein gene ontology were performed using the program Blast2GO (www.blast2go.com).

**Fig 3:**
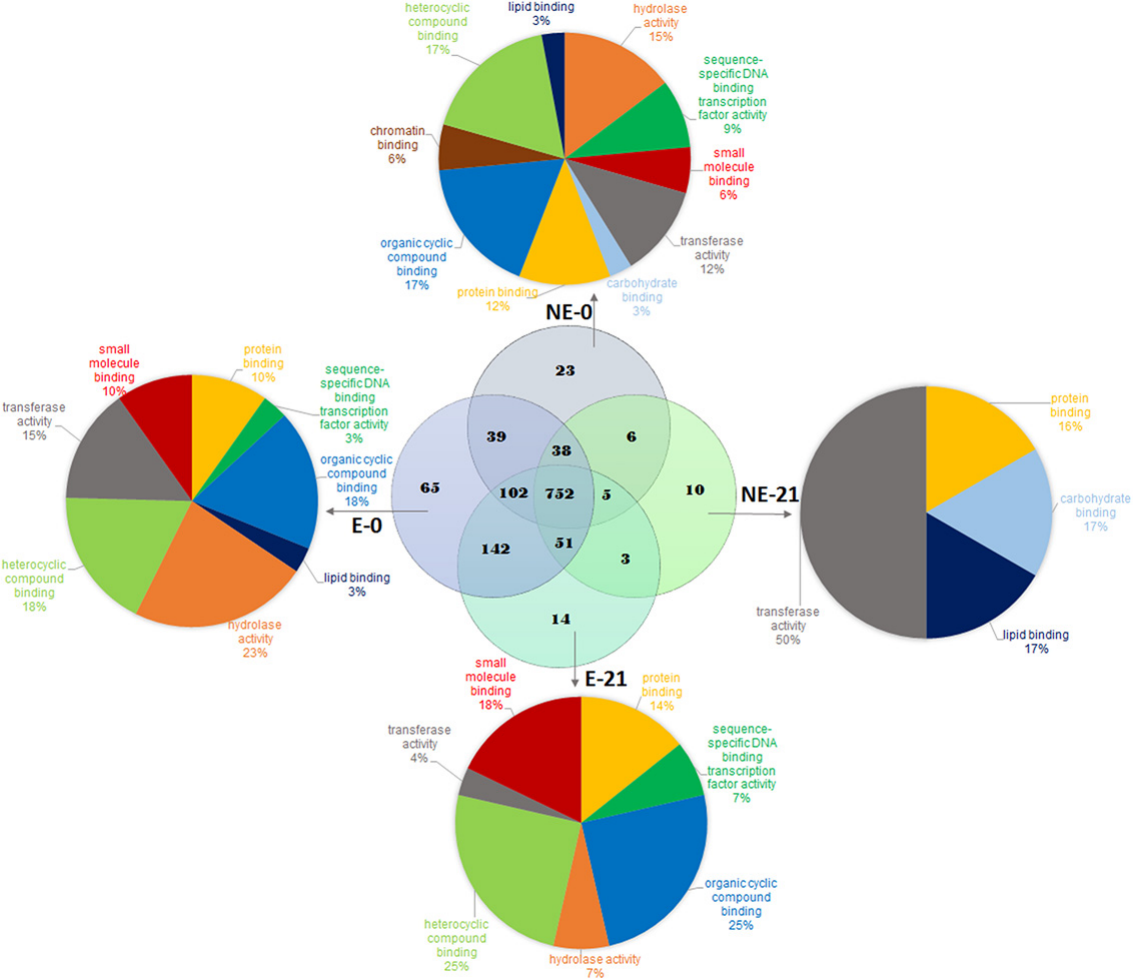
Venn diagram and pie charts displaying the numbers and functions of unique and co-expressed proteins. The number of unique and co-expressed proteins from embryogenic(E) and non-embryogenic (NE) callus during maturation treatment and the functional classification of unique proteins from embryogenic (E) and non-embryogenic (NE) callus at 0 and 21 days of maturation treatment (E-0, E-21, NE-0 and NE-21).

When the functional classifications ware performed on the four samples, approximately 23% of the 65 unique E-0 proteins were observed to possess hydrolase activity compared with 7% in the 14 unique E-21 proteins, 18% had heterocyclic compound binding activity, versus 25% in E-21, and 18% showed organic cyclic compound binding, versus 25% in E-21 (Fig 3). Lipid binding activity, which appeared in approximately 3% of the unique E-0 proteins, did not appear in E-21 (Fig 3). Of the 23 unique NE-0 proteins, approximately 6% had chromatin binding activity, which did not appear in the unique E-0 and E-21 proteins (Fig 3). In the NE-21 callus 50% of the unique proteins had transferase activity (Fig 3).

**Fig 4:**
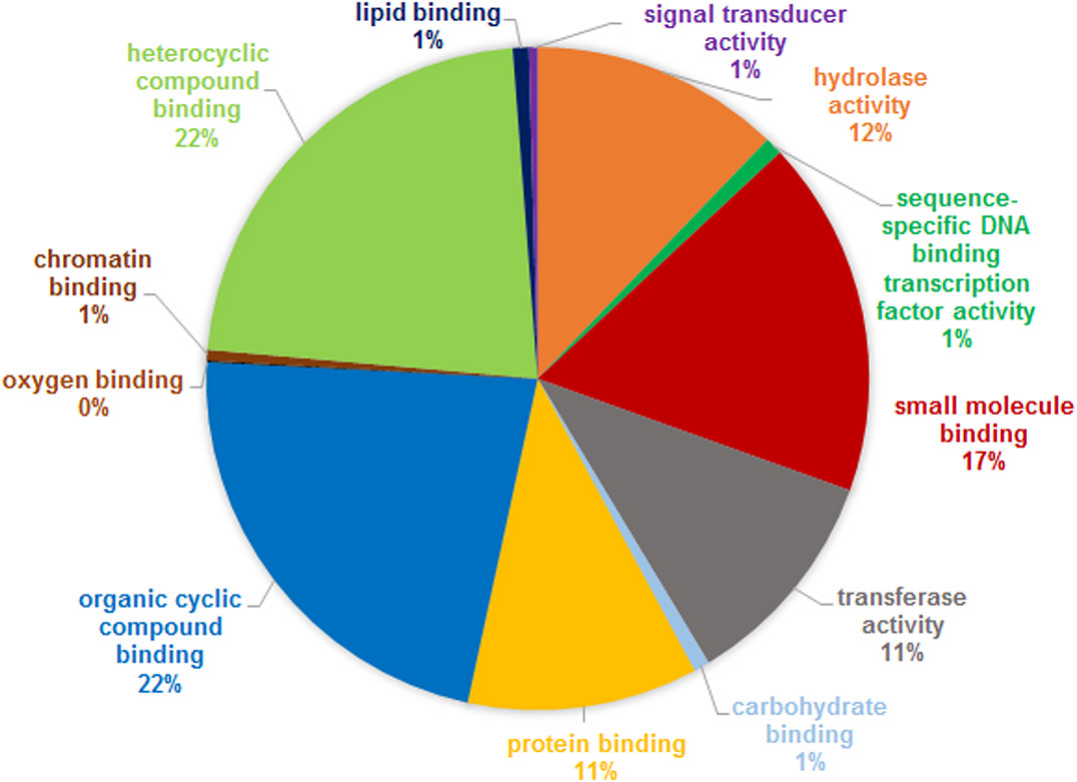
Pie charts showing the functional classification of the co-expressed proteins. Functional classification of the co-expressed proteins from embryogenic (E) and non-embryogenic (NE) callus before (0) and after 21 days of maturation treatment (E-0, E-21, NE-0 and NE-21).

When we analyzed the 752 proteins that were co-expressed in all callus samples, we observed that these proteins were categorized according to 3 main biological functions: 22% showed organic cyclic compound binding activity, 22% showed chromatin binding activity and 17% showed small molecule binding activity (Fig 4). E-0 callus had 163 proteins that were up-regulated and 129 that were down-regulated compared with NE-0 callus (S1 Table). When comparing callus between E-0 and E-21, 68 proteins were up-regulated, and 57 were down-regulated (S1 Table). The NE-0 callus had 107 proteins that were up-regulated compared with NE-21 callus and 111 that were down-regulated (S1 Table).

## Discussion

### Maturation and morphogenetic changes

Somatic embryo maturation and the progression to somatic seedling development represent bottlenecks in many species-specific somatic embryogenesis protocols. In the present work, supplementation with specific of concentrations AC (0.75 and 1.5 g L^-1^) was essential for somatic embryogenesis in sugarcane cv. SP80-3280 (Fig 2 and Table 1). The addition of 2,4-D into the culture medium is also necessary for induction and maintenance of sugarcane callus; this plant growth regulator (PGR) inhibits somatic embryo formation, the whereas removing 2,4-D from the culture medium is necessary for somatic embryogenesis evolution [13]. Furthermore, the addition of AC to the culture media caused a drastic decrease in endogenous levels of PGR and other organic supplements through the adsorption of these components [36], thereby promoting a decrease in the endogenous contents of 2,4-D content in sugarcane callus cultures. A likely explanation is that during the switch from auxin-containing to auxin-free culture medium, a residual amount of auxin travels with the callus, which can delay the evolution of somatic embryo development. Furthermore, in the AC addition, this auxin residue is rapidly adsorbed, thus preventing this PGR from inhibiting cell differentiation and permitting faster somatic embryo progression (Fig 2). However, the addition of AC to the culture medium will adsorb unwanted substances but may also adsorb some needed substances, such as macro- and micro-nutrients, vitamins and sucrose, which are important for somatic embryo development [36]. In this sense, the addition of high AC concentrations (3.0 g L^-1^) can disturb the formation of somatic embryos, as observed in this study (Fig 2 and Table 1). During maturation treatment, the culture medium supplemented with 3.0 g L^-1^ AC produced five times fewer somatic embryos (29) than did the treatment with the best AC concentration (1.5 g L^-1^), which promoted the formation of 158 somatic embryos at 14 days in culture (Fig 2 and Table 1). The excess AC may also have adsorbed essential substances for somatic embryo differentiation, such as macronutrients, micronutrients, vitamins, and sucrose, rendering them unavailable to the callus and preventing the formation of somatic embryos in sugarcane.

Some somatic cells, especially those of embryonic origin, possess embryogenic potential during their cell cycle. However this potential can diminish with the time, and one way to regain it is through an intermediary callus phase [17]. The transition from an embryonic cell to a somatic embryo depends on the embryogenic potential of these somatic cells. During this process, specific proteins may be expressed, that permit the resumption of embryogenic potential, culminating in the emergence of a totipotent cell that can give rise to a somatic embryo.

### Unique proteins that are expressed in embryogenic (E-0) callus

There are certain limitations to the identification of sugarcane proteins. First, because the sugarcane genome has not been sequenced. However, protein identification has been carried out using the sugarcane SUCEST EST database. Additionally, sugarcane possesses a polyploidy genome [37], and several cases have arisen in which different ESTs matched, causing redundancy in the identification of some proteins. Despite these difficulties, 2D-nanoESI-HDMS^E^ technology proved to be a good alternative in the identification of sugarcane proteins.

In shotgun proteomics analyses, 65 unique proteins were identified in E-0 callus (Table 2) and can be grouped into broad functional groups, such as high metabolic activity (Fig 3). These characteristic are common in the E callus culture of various species and are associated with the acquisition of competence [38–40].

Some proteins are highly related to intense cellular metabolic activity, a remarkable characteristic of E callus [41, 42]. E callus are formed by cells with prominent nuclei, culminating with a high nucleus:cytoplasm ratio [12, 13]. This high ratio translates to the high expression of proteins that fallow these cells to receive external signals and express their inherent potential in highlighting somatic embryo formation as the greatest essential characteristic of a totipotent cell [12]. Among these are the lsm sm-like protein (SCBGLR1098A04) (Table 2) which functions in mRNA metabolism in plants [43], peptidylprolyl cis-trans isomerase (SCQSRT1035C03) (Table 2), which has catalytic activity; coiled-coil protein (SCACAM1071C11) (Table 2), which functions in function of organizational and regulatory processes [44]; and sec14-like protein 1 (SCSBFL5016D03) (Table 2), which has transporter activity [45].

Another group of proteins is highly related to stress, which is an important factor in achieving embryogenic competence in plant cells. Stress induced by culture medium supplements, such as PGR, sucrose and AC, leads to changes the genetic reprogramming, which must take place at the chromatin level [38, 46], and consequently causes the expression of stress proteins, which leads to somatic embryo development. One of these proteins, the cation transport protein chac (SCBGLR1047G06) (Table 2), is associated with a general defensive response to various environmental stresses in plants [47]. Similarly, the protein polyphosphoinositide phosphatase-like isoform 1 (SCQGSB1143E08) (Table 2) is also associated with vesicular trafficking events and plant stress responses [48]. Another protein, copper-transporting p-type ATPase (SCVPRZ2044C07) (Table 2), is expressed in response to oxidative stress and hormone signaling [49]. The expression of the proteins drought-inducible protein 1os (SCQSST3116H01) (Table 2) [50] and S-adenosylmethionine:2-demethylmenaquinone methyltransferase-like (SCJFRZ2009B04) (Table 2) is also related to plant adaptation to drought stress [51]. The expression of these proteins suggests that the cultures are experiencing some type of stress and which may be responsible for the physiological modulation of the cells, thereby allowing the embryogenic competence [52].

In addition to these proteins, some of the unique E-0 callus proteins are closely related to E callus and somatic embryo characteristics (Table 2). Among these proteins, BURP domain-containing protein (SCSBHR1051A12) (Table 2) has been reported in plant microspores, and in somatic and zygotic embryogenesis, but its action in embryogenesis is not fully understood [53, 54] (Table 2). The BURP domain is conserved in diverse plant proteins, being found with divergent expression profiles and most likely divergent functions, suggesting that this protein is important and has a fundamental functional role [55]. Another protein closely related to somatic embryo formation is the dehydrogenase-like protein (SCRFAM1026C02) (Table 2), which is an enzyme that oxidizes a substrate through a reduction reaction in which one or more hydrides (H^-^) is transferred to an electron acceptor. Plants express many dehydrogenases, but we can highlight alcohol dehydrogenase 3 (ADH3). ADH3 represents a class of enzymes that may be related to a specific stage of embryonic development in *Vitis rupestris* [56], *Daucus carota [57]* and *Bactris gasipaes* Kunth [58] somatic embryos. Therefore, the function of this enzyme is important, among many other functions as the possible association of this protein with embryogenic competence for somatic embryo formation. In addition, hua enhancer 2 (HEN2) (SCVPLR1028B03) (Table 2) is a nuclear localized DExH helicase that plays a role in determining cell fate during late stages of floral development [59]. SKI2, an *Arabidopsis thaliana* DExH RNA helicase related to HEN2, is also associated with cell fate, affecting development during embryogenesis [60], which indicate that HEN2 may also be related to the somatic embryo development.

Some proteins were related to cell defences against biotic or abiotic stress, which can serve as a stimulus for the modulation of somatic embryogenesis, as a survival mechanism. In this context, nitric oxide synthase (NOS) interacting protein (SCQGHR1012E03) (Table 2), which synthesizes nitric oxide (NO), is unique in E-0 callus. In plants, NO serves as a signal in hormonal and defense response being a key signaling molecule in different intracellular processes [61]. NO can stimulate the activation of cell division and embryogenic cell formation in *Medicago sativa* [62], in addition to showing synergism with polyamines (PAs) in *Araucaria angustifolia* somatic embryogenesis [63]. Another stress response protein was the callose synthase 1 catalytic subunit (SCSFAD1108H10) (Table 2), a part of callose synthase, which is produced in response to wounding and pathogen attack [64] as well as during cell wall development [65] (Table 2). This enzyme is also expressed in *Arabidopsis* callus that are exposed to hyper-gravity [66] and in tomato seedlings that are exposed to salt stress [67]. In *Cichorium*, callose deposition seems to be an early marker in somatic embryogenesis [68]. Additional proteins were identified that are expressed in response to abiotic stress such as desiccation-related protein pcc13-62 expressed (SCBFSD1037G05) (Table 2) which is a protein that is up-regulated under desiccation or osmotic stress conditions or under the effect of abscisic acid (ABA) and does not depend on light for its expression [69, 70]. This protein is usually related to seeds whose embryos from undergo ABA signaling during desiccation and accumulate late embryogenesis-abundant (LEA) proteins, which are involved in desiccation resistance [69–71]. Such proteins are highly related to embryo formation and are present in E callus of sugarcane but not in NE callus, which have no potential for somatic embryo formation.

Other proteins were involved in metabolic pathways that may be anable the embryogenic potential of the cells. UDP-sugar pyrophosphorylase-like (SCVPCL6041D12) (Table 2) catalyzes the conversion of various monosaccharide 1-phosphates to the corresponding UDP-sugars, indicating a housekeeping function in plant [72]. One type of this enzyme, UDP-glucuronic acid, was also related to zygotic embryo development in *Glycine max* L., exhibiting a linear increase during this process[73]. In plants, saccharides act as carbon and energy sources, as well as osmotic agents and signal molecules [74]. Sucrose and hexoses exhibit similar endogenous saccharide patterns, regulating carbohydrate metabolism and being important for structural somatic embryo development [75]. In addition to sugar metabolism, vitamins may be involved in embryogenic competence. Folylpolyglutamate synthase (SCAGLR2033F09) (Table 2) catalyzes glutamylation following the initial glutamylation catalyzed by dihydrofolate synthase during plant folate synthesis [76, 77] (Table 2). The role of the vitamins biotin and folate are not fully known, but when combined with proline perhaps synergistically, these compounds permit high levels of somatic embryogenesis in hybrid onions (*Allium fistulosum* × *A*. *cepa* F1) [78].

Certain metabolic pathways, such as the tryptophan (Trp) pathway, may be highly related to embryonic competence. The protein 3-deoxy-d-arabino heptulosonate-7-phosphate synthase (SCEQRT1027A06) (Table 2) is the first enzyme of the shikimate pathway, which converts phosphoenolpyruvate (PEP) and erythrose 4-phosphate (E-4P) into 3-deoxy-D-arabino-heptulosonate 7-phosphate (DAHP) [79]. The shikimate pathway of plants mediates the conversion of primary carbon metabolites into phenylalanine (Phe), tyrosine (Tyr) and tryptophan (Trp), and numerous secondary metabolites that are derived from these metabolites [80] may be important for the acquisition of cell competence to develop somatic embryos in sugarcane callus. Along these same lines is phosphoribosylanthranilate transferase (SCAGLR1021B10) (Table 2). It is an enzyme that converts anthranilate and phosphoribosylpyrophosphate (PRPP) into phosphoribosylanthranilate (PR-anthranilate) and inorganic pyrophosphate in the Trp biosynthetic pathway [81]. Trp is the substrate for the synthesis of indoleacetic acid (IAA) [82], an important auxin for E callus induction. The expression of this protein in E-0 callus of sugarcane could be associated with the competence of these callus to develop somatic embryos during maturation treatments.

The balance of endogenous plant hormones plays a key role in somatic embryogenesis [83]. In this context along with the participation of other proteins in the formation of IAA as seen above, adenylyl-sulfate kinase (SCQSST1036B01) (Table 2) participates in the brassinosteroids biosynthesis pathway, where it catalyzes the ATP-dependent synthesis of adenosine 3'-phosphate 5'-phosphosulfate (PAPS), which is an essential metabolite for sulfur assimilation in plants [84]. PAPS serves as the sulfate donor for the sulfonation of brassinosteroids and peptide hormones [85]. Brassinosteroids are a class of plant steroid hormones whose functions are the promotion of cell elongation, cell division, and dedifferentiation [86]. A family of somatic embryogenesis receptor kinases (SERKs) has been genetically implicated in mediating early brassinosteroid signaling events [87], highlighting the importance of this PGR in somatic embryo development.

### Unique proteins that are expressed in maturated embryogenic (E-21) callus

When E callus are transferred to maturation medium culture supplemented with AC and are exposed to light for 21 days, their protein expression changes, leading to the identification of 14 unique proteins (Fig 3 and Table 2). Of these proteins, we can mention some that are related to E callus maturation.

Metabolic processes produce active oxygen species that are highly destructive to cellular components such as proteins, membrane lipids and nucleic acids [88]. Upon entering the maturation protocol, callus are exposed to intense light, which is a form of oxidative injury [89]. To minimize this effect, some proteins are differentially expressed in these callus, such as catalase-expressed (SCCCCL3120D10) (Table 2) and ac079853_1 myosin heavy chain-like (SCAGLB1071A03) (Table 2), representing one of the primary enzymatic defenses against oxidative stress [90, 91]. The TGACG-sequence-specific DNA-binding protein TGA-like (TGA) (SCJFSB1010B12) (Table 2) is involved in defense responses in Arabidopsis, but this function remains unknown [92].

Secreted proteins (SCSBSD2032F10) (Table 2) are of great importance for somatic embryogenesis because some of them have the ability to convert NE to E callus when added to the culture medium, such as like arabinogalactan proteins [93]. These proteins are likely to be used to enhance somatic embryogenesis protocols, to increase induction in non-responsive or low-embryogenic genotypes and to improve regeneration rates [94, 95]. In these present study, the expression of this protein leads us to believe that this protein is also important in the maturation phase, during which we observed somatic embryo development in sugarcane. The maturation and conversion phases for sugarcane somatic embryos still present a bottleneck against generating a high-efficiency regenerative protocol, and these proteins could help to improve these rates.

### Unique proteins expressed in non-embryogenic (NE-0) and maturated non-embryogenic (NE-21) callus

E and NE callus have different phenotypes and hence, different proteomes. However, NE-0 callus expressed proteins with similar functions to those expressed in E-0 and E-21 callus, save for proteins with lipid binding and chromatin binding activities (Fig 3). These may represent differentially expressed proteins, that provided the characteristics that are unique to NE callus. Among the proteins with lipid binding activity, pleckstrin homology domain-containing protein 1 (SCBGRT1046F12) (Table 2) interacts with the important, second messenger phosphatidylinositol 3,4,5-trisphosphate and may regulate specific cellular processes [96]. This binding can block external signals prevent response to these stimuli, in contrast to E callus, in which the cells would respond. Chromatin binding activity is found in the MYB-like DNA-binding domain containing protein (SCQGST1032A06) (Table 2), which is a transcription factor that is found in almost all eukaryotes [97]. In plants, MYB proteins regulate a vast array of metabolic, cellular and developmental processes and can act as transcriptional activators, repressors, or both [98]. Thus, this transcription factor may act on the differential expression of specific proteins in NE callus or may block the transcription of proteins that are essential in E callus, causing NE callus to lose the capacity to differentiate into somatic embryos. A few unique proteins (10) appeared in the NE-21 callus. One of these proteins was differentially, polyubiquitin-like protein (SCRLLV1026A01) (Table 2), which participates in the ubiquitin-dependent proteolytic pathway in which ubiquitin covalently attaches to proteins to be degraded [99]. The close relationship of protein to the proteolytic pathway may explain the low number of unique proteins in NE-21 callus, as many of these proteins may have been degraded. These10 unique proteins seem to indicate that the lack of-embryogenic induction in the NE callus is largely determined by the absence of proteins that are present in the E callus, rather vice versa.

### Co-expressed proteins in E and NE callus

The co-expressed proteins in all four types of callus predominantly consist of housekeeping proteins or proteins that are essential for the maintenance of basal cellular metabolism, such as chaperones, which promote correct protein folding (Fig 4 and S1 Table). These housekeeping proteins have been identified in several studies and are considered Déjà vu proteomics [100]. With this, it is worth mentioning only a few up-regulated proteins that are directly related to the development of somatic embryos.

Because these proteins appear in all callus, one would think that none of these proteins would play a key role in somatic embryogenesis and the development/conversion of somatic embryos. However, these proteins do not appear at the same concentration in all callus and may be up-regulated or down-regulated in relation to one another. Comparing the E-0 and NE-0 callus, the embryo-specific protein (SCCCCL3005C05.b) (S1 Table) appears up-regulated more than 15 times in E-0 callus, indicating that this callus culture is competent to develop somatic embryos (S1 Table). The late-embryogenesis-abundant group 3 protein variant 2 (SCQSRT2031H06) (S1 Table) and late-embryogenesis-abundant protein (SCJLLR1107G02) (S1 Table), an important class of proteins that are well known as markers of somatic embryos maturity, are also up-regulated more than 5 times in the E-0 callus (S1 Table). Proteins that are up-regulated in sugarcane E-0 callus may be associated with the acquisition of competence to develop somatic embryos, and the higher metabolism of embryogenic cells in E-0 could induce these changes in protein expression.

In E-21 callus, germin protein-like protein subfamily 1 member 17 precursor (SCCCCL7037E09) was identified (S1 Table), which is up-regulated almost 12 times compared E-0 callus (Table 2). Germins are thought to play a significant role during zygotic and somatic embryogenesis [101], indicating that the maturation treatment of E-21 callus led to an increase in the expression of this protein, thereby resulting in the development of somatic embryos in sugarcane. The embryo-specific protein (SCCCCL3005C05.b) (S1 Table) also appears up-regulated more than 35 times in E-21 compared with E-0, suggesting that this protein as an excellent marker of embryogenesis. The kinase interacting protein 1 (CIPK) (SCBFFL5072F08) up-regulated more than 119 times in E-21 compared with E-0. This protein is a target of Ca^2+^ sensors (calcineurin B-likeproteins [CBLs]) [102]. Ca^2+^ is a important second messenger, and the CBL-CIPK signaling network translates Ca^2+^ signals them into the correct cellular responses [102]. CBL and CIPK proteins are important components of abiotic stress responses, hormone reactions and ion transport processes in plants [103], indicating that all of these processes can be crucial for the development of sugarcane somatic embryos.

## Conclusions

The best treatment to promote somatic embryo maturation in sugarcane cv. SP80-3280 was MS culture medium supplemented with AC 1.5 g L^-1^, which led to the highest number of somatic embryos in E callus. In NE callus the formation of somatic embryos was not observed with any of the applied treatments.

The unique proteins found in the E callus, including dehydrogenase-like protein, desiccation-related protein-62 pcc13, callose synthase 1 catalytic subunit and NOS-interacting protein could play an important role in sugarcane morphogenesis and embryogenic competence, permitting the formation of somatic embryos. The NE callus exhibited more unique proteins that were related to protein degradation, which is indicative of callus with low metabolic activity and, consequently, a low level of cell differentiation, which therefore do not form somatic embryos. The unique proteins in E-21 callus, such as catalase-expressed and secreted protein could play an important role in the maturation of sugarcane somatic embryos. These proteins could be potential candidates for use in enhancing somatic embryogenesis protocols, potentially by increasing induction in non-responsive or low-embryogenic genotypes and improving regeneration rates. The co-expressed proteins were presented at high levels, and a crude analysis revealed that many of these proteins were involved in housekeeping functions. However, the expression levels of these proteins, whether down- or up-regulated, may indicate a role in certain important morphogenetic pathways, such as the embryo-specific protein in E-0 and E-21 callus and germin protein-like protein subfamily 1 member 17 precursor and kinase interacting protein 1 in E-21 callus. Thus, these proteins may also represent candidate biochemical markers for different stages of sugarcane somatic embryogenesis.

## Supporting Information

S1 Fig Histomorphological aspects of embryogenic and non-embryogenic callus. Embryogenic (A) and non-embryogenic (B) callus of sugarcane var. SP80-3280 on day 0 of maturation treatment submitted to histomorphological analyses. SE: somatic embryos; bars: A 500 μm; B 200 μm.
(TIF)

S1 Protocol Histological analysis protocol. Histological analysis by light microscopy to demonstrate histomorphological differences between embryogenic (E) and non-embryonenic (NE) sugarcane cell cultures.
(DOCX)

S1 Table Co-expressed proteins identified in the embryogenic and non-embryogenic sugarcane cultures submitted to maturation treatments. (DOCX)

S2 Table Non-exclusive proteins identified in the embryogenic and non-embryogenic sugarcane cultures submitted to maturation treatments. (DOCX)
