# Supplementary figures and images for: Label-Free Quantitative Proteomics of Embryogenic and Non-Embryogenic Callus during Sugarcane Somatic Embryogenesis
Source: PLoS One. 2015 Jun 2;10(6):e0127803. doi: 10.1371/journal.pone.0127803 (PMC4452777; doi:10.1371/journal.pone.0127803)

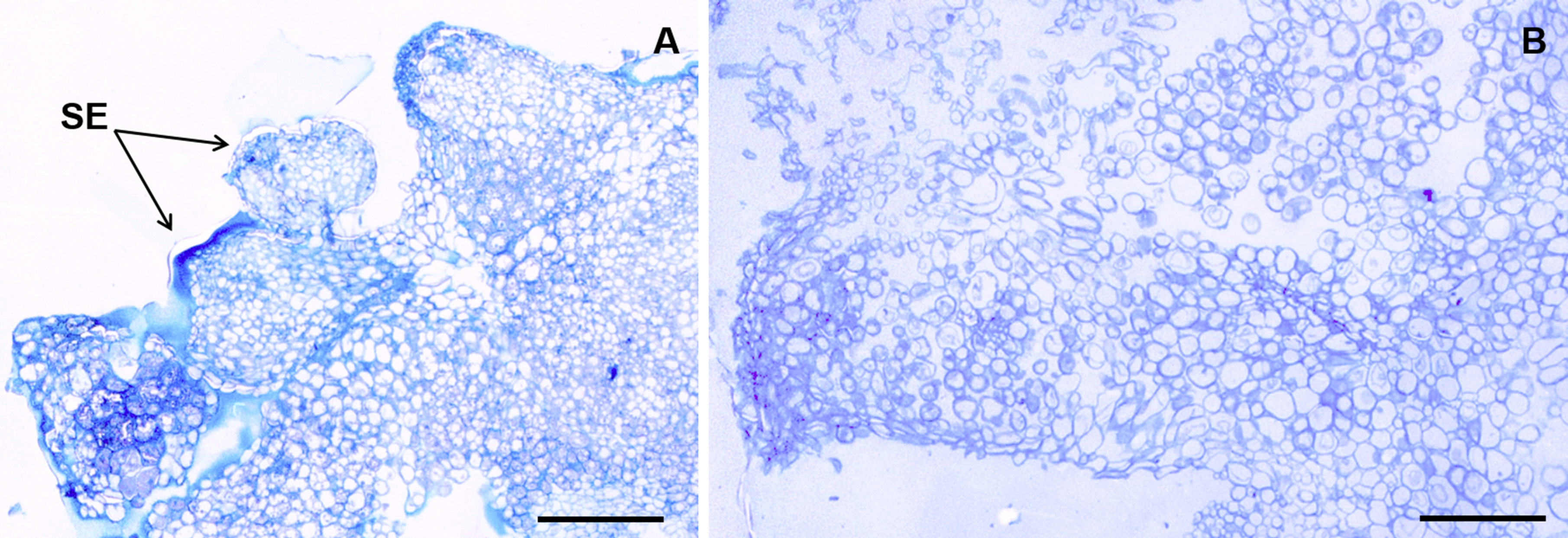

Supplement: S1 Fig — Embryogenic (A) and non-embryogenic (B) callus of sugarcane var. SP80-3280 on day 0 of maturation treatment submitted to histomorphological analyses. SE: somatic embryos; bars: A 500 μm; B 200 μm. (TIF) [file pone.0127803.s001.tif]
