# Supplementary material for: Label-Free Quantitative Proteomics of Embryogenic and Non-Embryogenic Callus during Sugarcane Somatic Embryogenesis
Source: PLoS One. 2015 Jun 2;10(6):e0127803. doi: 10.1371/journal.pone.0127803 (PMC4452777; doi:10.1371/journal.pone.0127803)
Supplement: S1 Protocol — Histological analysis by light microscopy to demonstrate histomorphological differences between embryogenic (E) and non-embryonenic (NE) sugarcane cell cultures. (DOCX) [file pone.0127803.s002.docx]

**Histological analyses**

Samples from E and NE cultures at day 0 ware used for histological analysis, in order to demonstrate the histomorphological differences between these two types of sugarcane cell cultures. Samples were fixed with 2.5 % glutaraldehyde (Merck, Darmstadt, Germany) and 4 % p-formaldehyde (Merck) in 100 mM sodium cacodylate (pH 7.2) (Merck) for 24 h, followed by dehydration with an increasing series of aqueous ethanol solutions. After dehydration, the samples were infiltrated with HistoResin (Leica® HistoResin, Heidelberg, Germany). Sections (approximately 5 µm thick) were cut and stained with a 1 % toluidine blue solution (Sigma-Aldrich). After water evaporation, sections were stained with a 1 % toluidine blue solution (Sigma-Aldrich). The sample sections were observed under an Axioplan light microscope (Carl Zeiss, Jena, Germany) equipped with an Axiocam MRC5 digital camera (Carl Zeiss), and the images were analyzed using AxioVisionLE version 4.8 software (Carl Zeiss).
