# Supplementary material for: Label-Free Quantitative Proteomics of Embryogenic and Non-Embryogenic Callus during Sugarcane Somatic Embryogenesis
Source: PLoS One. 2015 Jun 2;10(6):e0127803. doi: 10.1371/journal.pone.0127803 (PMC4452777; doi:10.1371/journal.pone.0127803)
Supplement: S1 Table — (DOCX) [file pone.0127803.s003.docx]

**Table S1.** Co-expressed proteins identified in the embryogenic and non-embryogenic sugarcane cultures submitted to maturation treatments.

| SUCEST accession number | | Description | Peptide count | | Peptides used for quantitation | | Confidence score | | Highest mean condition | | Lowest mean condition | | E-0 | NE-0 | E-21 | NE-21 |
| --- | --- | --- | --- | --- | --- | --- | --- | --- | --- | --- | --- | --- | --- | --- | --- | --- |
| >SCEZAD1078B05 | | curcin precursor | 1 | | 1 | | 3.9787 | | NE-0 | | E-0 | | 10.25848 | 13622.64 | 34.16956 | 137.015 |
| >SCJFST1010G02 | | capp1_sachy | 11 | | 6 | | 77.9309 | | NE-0 | | NE-21 | | 8348.267 | 19232.39 | 9256.739 | 359.7991 |
| >SCACSD1017D11 | | clone 205-like isoform 1 | 17 | | 4 | | 152.9226 | | E-0 | | NE-21 | | 8234.341 | 7809.416 | 700.9264 | 201.4516 |
| >SCCCCL4014A08 | | geranylgeranyl pyrophosphate synthase 4 | 4 | | 2 | | 20.7251 | | E-0 | | NE-21 | | 8534.896 | 2470.607 | 959.5335 | 109.0277 |
| >SCCCCL3005C05.b | | embryo-specific protein | 13 | | 12 | | 141.2254 | | E-21 | | NE-21 | | 2066.585 | 132.5363 | 73345.13 | 7.288948 |
| >SCJLLR1101G05 | | hmgi y protein | 2 | | 1 | | 10.4044 | | NE-0 | | NE-21 | | 340.7214 | 3706.119 | 1699.157 | 210.8935 |
| >SCEPRZ1008D03 | | fen11_sorbi | 4 | | 3 | | 27.7696 | | NE-0 | | NE-21 | | 1706.446 | 12984.94 | 2431.46 | 893.5078 |
| >SCQGFL3053H05 | | lz-nbs-lrr class rga | 2 | | 1 | | 9.8136 | | NE-0 | | E-0 | | 93.04693 | 4901.694 | 170.659 | 359.549 |
| >SCACHR1041H05 | | cyclic phosphodiesterase | 3 | | 2 | | 19.7656 | | NE-0 | | NE-21 | | 2139.018 | 3541.926 | 3345.375 | 339.5296 |
| >SCCCRZ1001H06 | | co-chaperone protein sba1 | 6 | | 4 | | 64.0411 | | NE-0 | | NE-21 | | 19605.58 | 48914.92 | 16330.74 | 4786.291 |
| >SCSBFL1101H09.b | | integral membrane single c2 domain protein | 5 | | 2 | | 33.0867 | | E-21 | | NE-21 | | 12021.12 | 6676.68 | 45850.57 | 660.4339 |
| >SCCCCL3002E04.b | | trehalose-phosphate synthase | 22 | | 10 | | 121.5711 | | E-0 | | NE-21 | | 9493.06 | 1060.605 | 4030.1 | 106.3465 |
| >SCSGHR1066E06 | | aspr2 protein | 23 | | 8 | | 147.6478 | | E-0 | | NE-21 | | 9149.26 | 3882.674 | 6048.93 | 410.2096 |
| >SCCCLR1069D07 | | guanylate binding protein | 10 | | 6 | | 52.0733 | | NE-0 | | NE-21 | | 2752.892 | 4093.555 | 1298.486 | 469.5092 |
| >SCQSST1038D08 | | sec12-like protein 2 | 3 | | 2 | | 16.7043 | | NE-0 | | E-21 | | 464.894 | 3901.299 | 246.8576 | 478.6646 |
| >SCJFRT2055E11.b | | beta-expansin 1a precursor | 19 | | 12 | | 127.8494 | | NE-0 | | E-21 | | 7773.25 | 52332.28 | 4657.187 | 6564.604 |
| >SCVPLR2019F03 | | asf sf2-like pre-mrna splicing factor srp32 | 7 | | 5 | | 47.9284 | | NE-0 | | NE-21 | | 1536.824 | 4504.274 | 2525.114 | 571.4726 |
| >SCCCRZ2C04F02 | | amidase family expressed | 14 | | 6 | | 85.3448 | | NE-0 | | E-0 | | 6258.07 | 73499 | 7947.87 | 9652.354 |
| >SCCCHR1002G07 | | gpi-anchored protein | 9 | | 4 | | 66.7657 | | E-0 | | NE-21 | | 44154.71 | 10450.31 | 33538.33 | 1372.436 |
| >SCEZLB1007F05 | | clpb3_orysj | 26 | | 15 | | 153.2617 | | NE-0 | | E-0 | | 9259.183 | 73881.34 | 17871.11 | 9921.871 |
| >SCQGHR1013D03 | | conserved oligomeric golgi complex subunit 5-like | 6 | | 5 | | 34.8339 | | E-0 | | NE-21 | | 22699.32 | 3663.37 | 1371.498 | 529.2136 |
| >SCEQLB1066E08 | | loc100284659 precursor | 4 | | 3 | | 26.3181 | | NE-0 | | NE-21 | | 606.9579 | 1692.314 | 885.4945 | 252.5909 |
| >SCSGST1072D03 | | calreticulin precursor | 19 | | 10 | | 122.667 | | NE-0 | | NE-21 | | 4049.291 | 25884.97 | 10858 | 4047.466 |
| **Table S1.** Continued |  | | |  | |  | |  | |  | |  |  |  |  |  |
| >SCJFLR1017A04 | | heat- and acid-stable phosphoprotein | 2 | | 2 | | 21.9397 | | E-21 | | NE-21 | | 5373.2 | 1135.684 | 5951.419 | 183.0112 |
| >SCAGSD1039H04 | | anthranilate n-benzoyltransferase protein 1 | 8 | | 6 | | 44.6412 | | NE-0 | | NE-21 | | 2725.669 | 5881.267 | 2081.17 | 971.665 |
| >SCEZRZ1015B08 | | phg1a protein | 7 | | 7 | | 31.9824 | | E-0 | | E-21 | | 5399.204 | 4235.963 | 257.7837 | 699.885 |
| >SCCCRT1003G09 | | isoform 1 | 10 | | 5 | | 62.8079 | | E-21 | | NE-21 | | 20111.49 | 12259.47 | 31147.98 | 2061.882 |
| >SCCCRT1001G11 | | lipid transfer protein | 15 | | 9 | | 116.9914 | | NE-0 | | E-0 | | 10859.78 | 257523.7 | 25568.72 | 43430.11 |
| >SCJFLR1074H09 | | nhp2-like protein 1 | 11 | | 8 | | 81.4932 | | E-0 | | NE-21 | | 108098.6 | 18676.75 | 26903.21 | 3389.828 |
| >SCEZLB1009D02 | | u2 small nuclear ribonucleoprotein a | 15 | | 11 | | 98.1446 | | NE-0 | | NE-21 | | 34323.76 | 95823.74 | 60557.71 | 18442.41 |
| >SCCCCL3003B01.b | | cbs domain protein | 25 | | 18 | | 284.1879 | | E-21 | | NE-21 | | 233909.2 | 169165 | 296230.9 | 33223.86 |
| >SCJFRT1012F01 | | protein-l-isoaspartate o-methyltransferase-like... | 5 | | 4 | | 28.1116 | | E-21 | | NE-21 | | 3393.302 | 5690.942 | 7655.616 | 1138.87 |
| >SCJLRT1015F05 | | osmotin-like protein precursor | 22 | | 17 | | 256.641 | | E-0 | | NE-21 | | 91860.46 | 29323.84 | 52880.13 | 6145.986 |
| >SCJLLR1104H07 | | phytocyanin-related protein pn14 | 2 | | 1 | | 12.5023 | | NE-0 | | NE-21 | | 4109.405 | 4405.142 | 2401.979 | 944.3243 |
| >SCCCAM2003E05 | | pirin-like protein | 4 | | 2 | | 21.3422 | | E-0 | | NE-21 | | 3865.225 | 724.1863 | 1579.028 | 160.8459 |
| >SCMCRT2089E02 | | pdc1_maize | 18 | | 8 | | 159.9512 | | E-0 | | NE-21 | | 21547.95 | 16655.42 | 12178.37 | 3739.72 |
| >SCEQRT2029D07 | | gh34_orysj | 2 | | 1 | | 11.7119 | | NE-0 | | NE-21 | | 3544.695 | 5166.808 | 2315.291 | 1169.686 |
| >SCSGFL1078A05 | | phosphopantothenate--cysteine ligase | 6 | | 5 | | 37.6406 | | NE-0 | | NE-21 | | 5093.587 | 10312.33 | 3254.986 | 2350.502 |
| >SCCCCL3001B10.b | | b chain semi-active e176q mutant of rice a plant... | 21 | | 7 | | 122.9118 | | E-0 | | NE-21 | | 3371.559 | 1728.38 | 1108.885 | 403.5121 |
| >SCSFFL4082E10 | | nnja4_maize | 1 | | 1 | | 8.6336 | | E-21 | | NE-21 | | 1678.457 | 4011.469 | 4376.204 | 949.1452 |
| >SCSBFL1043A02 | | pro-resilin precursor | 10 | | 9 | | 62.24 | | E-21 | | NE-21 | | 10608.7 | 704.6884 | 44374.02 | 167.8921 |
| >SCBFRZ2045E10 | | tpr domain containing protein | 22 | | 11 | | 180.3759 | | E-0 | | NE-21 | | 37738.6 | 16845.21 | 26775.9 | 4097.186 |
| >SCAGLR1043F02 | | 70kda heat shock protein | 59 | | 6 | | 566.1407 | | NE-0 | | E-21 | | 4346.079 | 23436.34 | 2679.956 | 5870.194 |
| >SCEQRT2028H08 | | phosphate chloroplast expressed | 11 | | 6 | | 84.1807 | | E-0 | | NE-21 | | 8438.567 | 1055.179 | 3173.679 | 270.2784 |
| >SCBGRT3074G12 | | loc100284334 precursor | 20 | | 12 | | 185.3751 | | NE-0 | | E-21 | | 8581.919 | 736176.5 | 4897.184 | 188638.7 |
| >SCEQRT1028C10 | | spo76 protein | 13 | | 6 | | 80.2677 | | E-0 | | NE-21 | | 9605.116 | 8445.394 | 4620.278 | 2175.576 |
| >SCRURT2005G02 | | indole-3-glycerol phosphate chloroplastic-like... | 6 | | 4 | | 36.94 | | NE-0 | | NE-21 | | 14135.53 | 14321.38 | 5682.16 | 3824.566 |
| >SCBFRZ2050A05 | | af384033_1 hd2 type histone deacetylase hda106 | 7 | | 4 | | 49.4199 | | E-0 | | NE-21 | | 12772.42 | 4948.467 | 7429.606 | 1339.628 |
| >SCCCRZ2001E10 | | dna-directed rna polymerase ii | 46 | | 25 | | 275.2676 | | NE-0 | | NE-21 | | 26701.7 | 37556.64 | 25187.14 | 10167.4 |
| >SCCCLR1001G12 | | gdp dissociation inhibitor | 14 | | 4 | | 106.7583 | | NE-0 | | E-21 | | 2354.357 | 9572.733 | 1681.266 | 2678.543 |
| **Table S1.** Continued |  | | |  | |  | |  | |  | |  |  |  |  |  |
| >SCSGAD1006C03 | | biotin synthase-like | 10 | | 6 | | 63.0486 | | E-0 | | NE-21 | | 84580.25 | 39993.52 | 40727.37 | 11197.23 |
| >SCJFRZ2025D08 | | hsc70-interacting protein | 26 | | 14 | | 211.5694 | | NE-0 | | NE-21 | | 16493.02 | 49180.13 | 30251.18 | 13901.53 |
| >SCBFLR1026G11 | | fatty acid biosynthesis1 | 12 | | 6 | | 81.2758 | | E-0 | | NE-21 | | 15541.13 | 3123.664 | 10036.26 | 902.6691 |
| >SCEZHR1087D09 | | mapeg family expressed | 5 | | 3 | | 54.4549 | | NE-0 | | E-21 | | 10406.66 | 33158.59 | 7158.302 | 9605.61 |
| >SCCCCL3080H04.b | | glycine-rich rna-binding protein 2 | 48 | | 26 | | 350.4326 | | E-21 | | NE-21 | | 92617.99 | 114120.1 | 154965.6 | 33302.84 |
| >SCEZLB1010A08 | | pseudouridine synthase 1 | 8 | | 6 | | 39.6867 | | E-0 | | NE-21 | | 20770.94 | 4165.265 | 20038.1 | 1221.344 |
| >SCQSRT2031H06 | | late embryogenesis abundant protein group 3... | 45 | | 32 | | 376.3579 | | E-21 | | NE-21 | | 177221.4 | 27030.12 | 234399.9 | 8022.879 |
| >SCCCRT1001H12 | | root cap protein 2 precursor | 11 | | 8 | | 93.8942 | | E-0 | | E-21 | | 38817.4 | 33340.49 | 8497.131 | 9948.497 |
| >SCACLR1126F12 | | lea1_orysj | 10 | | 6 | | 79.8727 | | E-0 | | NE-21 | | 43667.15 | 4966.897 | 43497.36 | 1497.096 |
| >SCBFRZ2016F05 | | unknown [Zea mays] | 172 | | 85 | | 1491.7787 | | NE-0 | | NE-21 | | 661380.3 | 935162.7 | 665131.9 | 283858.6 |
| >SCAGLR1043H10 | | apobec1 complementation factor-like | 18 | | 7 | | 137.4153 | | E-0 | | NE-21 | | 20713.91 | 15950.42 | 13396.39 | 4931.739 |
| >SCCCSD2C04B03 | | loc100284192 precursor | 7 | | 4 | | 43.415 | | NE-0 | | NE-21 | | 21994.73 | 38170.73 | 15912.63 | 11811.97 |
| >SCQSRT2036C02 | | translocon-associated protein beta containing... | 10 | | 6 | | 79.2186 | | E-21 | | NE-21 | | 14024.08 | 19707.78 | 28219.55 | 6146.433 |
| >SCCCLR1079A08 | | membrane steroid-binding protein 1 | 20 | | 10 | | 151.2625 | | E-21 | | NE-21 | | 14975.57 | 14667.98 | 37465.62 | 4597.427 |
| >SCSGRT2063H01 | | tpa: class iii peroxidase 64 precursor | 105 | | 75 | | 1017.829 | | NE-0 | | NE-21 | | 479424.8 | 1356623 | 444147.7 | 430163.2 |
| >SCJLRZ1023B07 | | minichromosomal maintenance factor | 57 | | 30 | | 353.9359 | | E-0 | | NE-21 | | 40521.7 | 39564.19 | 35830.82 | 12636.96 |
| >SCCCAM1C04B04 | | 10 kda expressed | 10 | | 4 | | 97.7551 | | NE-0 | | NE-21 | | 17838.81 | 21316.56 | 15230.41 | 7011.072 |
| >SCRFLR2037C07 | | ac092172_32 activator-like transposable element | 2 | | 2 | | 10.8622 | | E-0 | | NE-21 | | 3569.356 | 2389.852 | 1391.673 | 787.8611 |
| >SCCCLR1C04B11 | | selenoprotein o | 26 | | 14 | | 152.3315 | | E-0 | | NE-21 | | 29454.99 | 15766.57 | 13607.03 | 5198.808 |
| >SCCCCL3140F04 | | proliferating cell nuclear antigen | 27 | | 15 | | 247.3734 | | NE-0 | | NE-21 | | 15967.64 | 39562.86 | 20641.73 | 13130.94 |
| >SCACST3159D04 | | inosine-5 monophosphate dehydrogenase 2 | 4 | | 1 | | 24.2281 | | NE-0 | | E-0 | | 1882.933 | 7943.754 | 2326.698 | 2638.164 |
| >SCRLFL4102C08 | | minichromosome maintenance deficient protein 5 | 29 | | 19 | | 186.5796 | | E-0 | | NE-21 | | 26021.58 | 7954.051 | 14852.67 | 2645.203 |
| >SCCCCL2001B11.b | | xip1_wheat | 14 | | 3 | | 117.1312 | | NE-0 | | E-21 | | 253.2493 | 11644.13 | 118.5275 | 3876.128 |
| >SCCCLR1070C10 | | af384035_1 nucleosome chromatin assembly... | 17 | | 10 | | 125.4361 | | NE-0 | | E-21 | | 58289.69 | 71809.95 | 22926.27 | 24254.25 |
| >SCEZRT2023B09 | | ac018727_1 ubiquinone oxidoreductase subunit | 4 | | 3 | | 21.492 | | E-21 | | NE-21 | | 1388.139 | 1926.904 | 2702.305 | 652.5547 |
| >SCEZRZ1014E01 | | nucleotide pyrophosphatase phosphodiesterase... | 21 | | 10 | | 121.9374 | | NE-0 | | E-21 | | 6471.613 | 24193.68 | 4606.337 | 8312.028 |
| >SCEZRZ3098F04 | | bronze-2 protein | 8 | | 5 | | 52.3352 | | NE-0 | | NE-21 | | 10240.39 | 19472.9 | 10080.76 | 6706.954 |
| **Table S1.** Continued |  | | |  | |  | |  | |  | |  |  |  |  |  |
| >SCEQLR1093H04 | | polyadenylate-binding protein2 | 11 | | 5 | | 86.1342 | | E-21 | | NE-21 | | 14654 | 18816.27 | 32509.32 | 6643.371 |
| >SCEQRT2026A06 | | blight-associated protein p12 precursor | 2 | | 2 | | 21.7547 | | NE-0 | | NE-21 | | 3505.567 | 6742.9 | 2908.875 | 2383.467 |
| >SCJFRZ2026B04 | | gmp synthase | 5 | | 5 | | 40.199 | | NE-0 | | E-0 | | 2453.387 | 20444.29 | 4106.639 | 7244.688 |
| >SCEQLR1007A06 | | profilin-2 | 7 | | 5 | | 70.4517 | | NE-0 | | E-21 | | 32190.84 | 50948.42 | 11465.8 | 18067.61 |
| >SCEQLR1050A01 | | kda proline-rich protein | 11 | | 7 | | 72.4064 | | E-0 | | NE-21 | | 68751.97 | 9868.948 | 42586.63 | 3515.349 |
| >SCEPCL6019C06 | | c3h14_orysj | 7 | | 3 | | 43.5165 | | E-0 | | NE-21 | | 2305.464 | 187.1959 | 680.2542 | 66.68867 |
| >SCJFRZ1006A12 | | ac077693_26 pyruvate kinase | 17 | | 7 | | 112.1933 | | E-21 | | NE-21 | | 2319.575 | 1870.484 | 3806.124 | 667.2333 |
| >SCCCCL3001E12.b | | arabinogalactan protein | 15 | | 11 | | 99.1966 | | NE-0 | | NE-21 | | 17637.34 | 42757.71 | 34845.07 | 15313.77 |
| >SCVPAM2065H07 | | uracil phosphoribosyltransferase | 7 | | 5 | | 40.6418 | | E-0 | | NE-21 | | 6171.491 | 3828.115 | 4410.603 | 1379.854 |
| >SCCCLR1070C03 | | ef hand family expressed | 16 | | 12 | | 108.3608 | | E-0 | | E-21 | | 29997.77 | 25269.17 | 8682.702 | 9301.994 |
| >SCCCRZ1C01A05 | | nls receptor | 22 | | 16 | | 156.4408 | | E-21 | | NE-21 | | 39921.35 | 18789.3 | 48691.69 | 6985.96 |
| >SCBGLR1002H03 | | prefoldin subunit 3 | 33 | | 21 | | 268.1885 | | E-21 | | NE-21 | | 27090.14 | 28534.46 | 45107.43 | 10781.71 |
| >SCCCLR2002F10 | | histidine triad nucleotide binding protein | 21 | | 14 | | 169.6583 | | NE-0 | | NE-21 | | 33954.6 | 51995.42 | 26408.3 | 19702.24 |
| >SCEZHR1048F05 | | 3-hydroxyisobutyryl-coenzyme a hydrolase | 23 | | 16 | | 160.228 | | NE-0 | | NE-21 | | 31381.12 | 51275.9 | 27912.75 | 19434.1 |
| >SCJLRZ1024H10 | | myosin-like protein | 23 | | 12 | | 154.1587 | | NE-0 | | NE-21 | | 12377.58 | 17318.26 | 9766.813 | 6605.962 |
| >SCCCCL3120C04 | | superoxide dismutase 2 | 3 | | 1 | | 17.253 | | E-0 | | NE-21 | | 3161.909 | 1950.595 | 1517.485 | 746.9981 |
| >SCJLRT1019E04 | | early fruit mrna | 19 | | 9 | | 105.023 | | E-0 | | NE-21 | | 7732.982 | 3505.157 | 4971.766 | 1344.603 |
| >SCCCST1001B03 | | ruvb-like 2 | 13 | | 7 | | 77.7325 | | E-21 | | E-0 | | 1878.077 | 5972.429 | 13335.03 | 2324.254 |
| >SCJLLR2013G12 | | fusca homolog | 15 | | 9 | | 85.1998 | | NE-0 | | E-21 | | 13609.49 | 22565.26 | 6222.655 | 8856.839 |
| >SCCCLR1066F11 | | gamm1 protein | 16 | | 10 | | 97.8087 | | E-0 | | NE-21 | | 11825.14 | 11379.9 | 7520.187 | 4483.329 |
| >SCJLRZ1027D12 | | methylthioribosE-0-phosphate isomerase | 5 | | 4 | | 42.4532 | | E-0 | | NE-21 | | 37593.42 | 14877.64 | 33784.29 | 5875.188 |
| >SCRLCL6030A07 | | acyl carrier protein 3 | 27 | | 16 | | 243.7388 | | NE-0 | | E-21 | | 54251.96 | 127953.3 | 29482.91 | 50534.39 |
| >SCCCCL4012D10 | | polypyrimidine tract-binding protein homolog... | 9 | | 6 | | 49.0548 | | E-0 | | NE-21 | | 11850.44 | 1754.976 | 6229.037 | 698.445 |
| >SCCCCL5071F02 | | anionic peroxidase | 37 | | 24 | | 375.2503 | | E-0 | | NE-21 | | 695185.7 | 300182.6 | 560647.3 | 120625.8 |
| >SCQSFL3038A12 | | nuclear transport factor 2 | 125 | | 66 | | 752.1508 | | E-21 | | NE-21 | | 158210.2 | 56319.1 | 201196.1 | 23549.18 |
| >SCCCLR1C07H02 | | calmodulin | 22 | | 15 | | 192.2242 | | E-0 | | NE-21 | | 155405.9 | 121929.5 | 87987.89 | 51479.43 |
| >SCQGLR2010A04 | | tubulin alpha-3 chain | 118 | | 19 | | 1183.5289 | | E-21 | | NE-21 | | 96449.7 | 71487.64 | 110280.8 | 30669 |
| **Table S1.** Continued |  | | |  | |  | |  | |  | |  |  |  |  |  |
| >SCMCRT3082C04 | | rickettsia 17 kda surface antigen family protein | 4 | | 3 | | 41.4123 | | E-0 | | NE-21 | | 19627.27 | 14559.83 | 14262.45 | 6272.105 |
| >SCJLRT1013H07 | | electron transfer flavoprotein beta-subunit | 14 | | 9 | | 101.8223 | | E-0 | | NE-21 | | 29026.61 | 16117.72 | 12190.46 | 6959.043 |
| >SCACLR2014A07 | | threonyl-trna synthetase | 39 | | 19 | | 219.2549 | | E-0 | | NE-21 | | 27243.44 | 15978.9 | 24832.23 | 6934.326 |
| >SCQSRT1035E10 | | phosphoserine aminotransferase | 30 | | 21 | | 265.2805 | | NE-0 | | E-0 | | 45810.18 | 130091.8 | 51935.27 | 56653.01 |
| >SCSFSB1106D06 | | nadp-dependent oxidoreductase p2 | 93 | | 46 | | 585.0908 | | NE-0 | | E-0 | | 51182.41 | 125772.4 | 74921.01 | 54815.18 |
| >SCCCLR1072C07 | | vesicle tethering family expressed | 16 | | 5 | | 109.8345 | | E-21 | | NE-21 | | 4660.377 | 5967.63 | 7867.335 | 2619.233 |
| >SCSFRT2072E08 | | tbc1 domain family member 13-like | 2 | | 1 | | 10.6299 | | E-0 | | NE-21 | | 3026.915 | 2007.471 | 2188.935 | 884.346 |
| >SCJLLR1107G01 | | auxin-induced protein pcnt115 | 77 | | 49 | | 578.9254 | | E-0 | | NE-21 | | 172096.8 | 117381 | 169095.1 | 52138.42 |
| >SCCCLB1021C06 | | adhesion regulating molecule conserved region... | 8 | | 5 | | 62.9888 | | E-0 | | E-21 | | 15207.54 | 9514.027 | 3550.521 | 4229.058 |
| >SCCCCL4015B08 | | skp1-like protein 1a | 22 | | 14 | | 170.9967 | | E-21 | | NE-21 | | 36501.18 | 16967.66 | 65200.61 | 7602.448 |
| >SCJLAM1061C04 | | diaminopimelate decarboxylase | 37 | | 27 | | 259.7618 | | E-0 | | NE-21 | | 75135.35 | 48813.05 | 41469.9 | 22111.24 |
| >SCEZRZ1016A01 | | nucleolar protein expressed | 56 | | 38 | | 383.7019 | | E-0 | | NE-21 | | 81335.09 | 74701.6 | 60340.3 | 34373.37 |
| >SCEPRZ1008C01 | | acyl-peptide hydrolase-like | 14 | | 6 | | 93.2828 | | E-0 | | NE-21 | | 44983.17 | 34311.9 | 27391.09 | 15857.23 |
| >SCCCLR1C02B03 | | beta 5 subunit of 20s proteasome | 47 | | 11 | | 363.7959 | | E-21 | | NE-21 | | 33825.91 | 45105.92 | 69495.13 | 20936.55 |
| >SCCCRZ1002F11 | | beta-tubulin r2242 | 57 | | 11 | | 587.7045 | | E-0 | | NE-21 | | 74278.85 | 71154.07 | 47624.72 | 33177.55 |
| >SCACST3159D04 | | inosine-5-monophosphate dehydrogenase 2 | 18 | | 9 | | 148.4253 | | NE-0 | | NE-21 | | 25164.12 | 33977.62 | 24968.15 | 15987.78 |
| >SCCCLB1025C04 | | nascent polypeptide-associated complex alpha... | 17 | | 11 | | 146.7821 | | E-0 | | NE-21 | | 130729.3 | 100740.1 | 74112.93 | 47564.47 |
| >SCCCRZ2C04C08 | | prostaglandin e synthase 3 | 5 | | 5 | | 41.8054 | | E-0 | | NE-21 | | 36751.34 | 16756.93 | 27338.45 | 7996.927 |
| >SCCCCL4017D10 | | ranbp1 domain containing protein | 25 | | 16 | | 133.1254 | | E-0 | | NE-21 | | 22850.61 | 10176.12 | 7463.292 | 4930.13 |
| >SCRFLR1034A04 | | sec13-related protein | 15 | | 11 | | 95.6432 | | E-21 | | NE-21 | | 60757.39 | 54676.09 | 81266.4 | 26532.86 |
| >SCCCRT2001A06 | | quinone oxidoreductase | 16 | | 12 | | 137.456 | | E-21 | | NE-21 | | 13029.31 | 9713.393 | 19095.86 | 4732.964 |
| >SCCCLR1072B04 | | glyceraldehyde 3-phosphate dehydrogenase | 51 | | 13 | | 665.9893 | | E-0 | | NE-21 | | 770775.4 | 518275.4 | 663792.3 | 252634.1 |
| >SCCCCL1002F12.b | | enoyl-acp reductase | 32 | | 22 | | 236.894 | | NE-0 | | NE-21 | | 71584.47 | 86746.15 | 81690.61 | 42496.99 |
| >SCSGRT2064D05 | | phd finger protein | 8 | | 3 | | 39.8759 | | NE-0 | | NE-21 | | 2888.132 | 3499.87 | 3039.767 | 1735.115 |
| >SCBFLR1046F09 | | bah domain containing protein | 7 | | 3 | | 42.5079 | | E-21 | | NE-21 | | 1265.306 | 311.4779 | 3171.289 | 154.9217 |
| >SCJFRT1008G12 | | somatic embryogenesis related protein | 15 | | 8 | | 89.0304 | | NE-0 | | NE-21 | | 22208.38 | 26163.01 | 24385.94 | 13041.2 |
| >SCCCLR1070B12 | | plasma membrane h+-atpase | 48 | | 15 | | 302.2677 | | E-21 | | NE-21 | | 26546.45 | 31342.83 | 53515.99 | 15646.09 |
| **Table S1.** Continued |  | | |  | |  | |  | |  | |  |  |  |  |  |
| >SCBFRZ2017F08 | | pantothenate kinase 4 | 28 | | 20 | | 167.9045 | | E-0 | | E-21 | | 27162.13 | 26163.18 | 6883.14 | 13114.91 |
| >SCCCCL2001F11.b | | clc1_orysj ame: full=clathrin light chain 1 | 9 | | 4 | | 53.2832 | | E-21 | | NE-21 | | 5855.767 | 2453.989 | 7414.976 | 1231.822 |
| >SCCCLR1078D11 | | sorting nexin 1 | 21 | | 7 | | 115.3347 | | NE-0 | | E-0 | | 2751.052 | 8326.759 | 3991.44 | 4192.45 |
| >SCEZRZ3101F09 | | formate dehydrogenase | 20 | | 15 | | 174.4554 | | E-21 | | NE-21 | | 73161.97 | 38089.35 | 100938.5 | 19220.87 |
| >SCMCLR1125D12 | | 116 kda u5 small nuclear ribonucleoprotein... | 15 | | 10 | | 116.2499 | | NE-0 | | E-21 | | 16955.85 | 31854.59 | 12511.69 | 16208.19 |
| >SCCCRZ2C03A09 | | 5-methyltetrahydropteroyltriglutamate... | 94 | | 66 | | 810.5021 | | NE-0 | | E-21 | | 293238.9 | 457924.3 | 221567.8 | 233482.2 |
| >SCCCRT3001A02 | | chitinase b | 28 | | 22 | | 295.0208 | | E-0 | | NE-21 | | 273470 | 173355.2 | 212346 | 88600.41 |
| >SCJFRZ1007F04 | | nadph--cytochrome p450 reductase-like | 2 | | 1 | | 8.8185 | | NE-0 | | E-21 | | 2736.054 | 5843.504 | 2344.567 | 2989.69 |
| >SCVPRT2081E09 | | endo- -beta-glucanase 2-like | 39 | | 22 | | 261.6547 | | NE-0 | | E-21 | | 62792.95 | 73869.21 | 28859.34 | 37804.78 |
| >SCCCRT1004A10 | | pre-mrna-splicing factor syf1-like | 68 | | 38 | | 444.4595 | | E-21 | | NE-21 | | 43103.42 | 31157.54 | 56412.76 | 15970.89 |
| >SCSGHR1069E04.b | | trehalose-6-phosphate synthase 2 | 36 | | 21 | | 224.2937 | | E-0 | | NE-21 | | 31125.3 | 13526.22 | 16347.45 | 6948.419 |
| >SCEZFL5090B07 | | glycosyltransferase [Saccharum officinarum] | 9 | | 6 | | 52.9845 | | E-0 | | NE-21 | | 10425.95 | 4539.957 | 4857.533 | 2334.337 |
| >SCCCLR1048G01 | | asparaginyl-trna cytoplasmic 3 | 51 | | 31 | | 357.4129 | | E-0 | | NE-21 | | 65650.15 | 46798.07 | 49548.2 | 24114.26 |
| >SCSBAD1087E01 | | aminoimidazolecarboximide ribonucleotide... | 15 | | 11 | | 93.4449 | | E-0 | | NE-21 | | 19893.84 | 5411.426 | 12979.9 | 2797.844 |
| >SCCCCL3080A03 | | ago4a_orysj ame: full=protein argonaute 4a... | 19 | | 10 | | 96.6411 | | E-0 | | E-21 | | 19268.61 | 11949.65 | 1192.382 | 6190.217 |
| >SCCCLR2004G08 | | 20 kda chloroplastic-like | 26 | | 18 | | 180.2457 | | E-21 | | NE-21 | | 33033.14 | 37050.59 | 41056.15 | 19197.54 |
| >SCJFRZ1007F10 | | es43 protein | 8 | | 3 | | 51.1088 | | E-0 | | NE-21 | | 5967.87 | 742.8884 | 4304.821 | 385.2131 |
| >SCCCCL6005E02 | | ornithine--oxo-acid aminotransferase | 22 | | 8 | | 138.9313 | | E-21 | | NE-21 | | 11669.21 | 17553.08 | 18791.48 | 9105.552 |
| >SCCCST3006H01 | | asparate aminotransferase | 6 | | 2 | | 30.4836 | | NE-0 | | E-21 | | 4783.492 | 14881.43 | 3698.923 | 7721.678 |
| >SCQGLR1041F04 | | n-alpha-acetyltransferase auxiliary subunit | 5 | | 3 | | 27.4206 | | NE-0 | | E-21 | | 3801.054 | 41138.45 | 2796.419 | 21447.35 |
| >SCQGLR1062G12 | | ac026815_7 rna binding protein | 8 | | 7 | | 45.5295 | | E-21 | | NE-21 | | 20049.11 | 12235.34 | 24078.52 | 6393.833 |
| >SCUTLR1058B03 | | atp-dependent rna helicase ddx23 | 180 | | 63 | | 1121.7103 | | NE-0 | | NE-21 | | 86808.51 | 117642.8 | 104460 | 61830.14 |
| >SCCCLR1048C04 | | ftsh8_orysj | 9 | | 5 | | 63.4664 | | NE-0 | | E-21 | | 2959.241 | 3328.018 | 649.5664 | 1757.965 |
| >SCSFSD1066D11.b | | kh domain containing protein | 39 | | 19 | | 261.8801 | | E-21 | | NE-21 | | 20699.47 | 27736.84 | 35455.16 | 14699.91 |
| >SCQGHR1013H09 | | sucrose cleavage | 174 | | 113 | | 1550.9992 | | NE-0 | | E-0 | | 456706.9 | 1250714 | 517858.5 | 668383.1 |
| >SCCCCL3140F07 | | plasminogen activator inhibitor 1 rna-binding... | 34 | | 18 | | 241.139 | | E-21 | | NE-21 | | 28525.99 | 6686.977 | 30684.71 | 3585.271 |
| >SCQSLR1018E09 | | nucleosome chromatin assembly factor group a | 64 | | 44 | | 510.2717 | | E-21 | | NE-21 | | 193137.7 | 147671.3 | 271009.1 | 79275.14 |
| **Table S1.** Continued |  | | |  | |  | |  | |  | |  |  |  |  |  |
| >SCCCCL3001B04.b | | prolyl-trna synthetase -like | 35 | | 27 | | 264.7046 | | NE-0 | | E-21 | | 58016.27 | 122779.3 | 41448.39 | 65924.91 |
| >SCCCRZ2001A04 | | 2-hydroxy-3-oxopropionate reductase | 19 | | 14 | | 106.7992 | | E-0 | | NE-21 | | 19029.83 | 10211.36 | 18397.93 | 5498.327 |
| >SCCCLR2C03F11 | | annexin p33 | 95 | | 61 | | 765.9604 | | NE-0 | | NE-21 | | 169923 | 259366.2 | 198689.3 | 140256.1 |
| >SCEZAD1081B12 | | leucyl-trna synthetase | 43 | | 25 | | 265.4449 | | NE-0 | | E-21 | | 50110.91 | 80105.01 | 24452.83 | 43410.34 |
| >SCCCRZ2001C11 | | usp family protein | 32 | | 20 | | 259.1192 | | E-21 | | NE-21 | | 61343.17 | 52697.75 | 133927.5 | 28634.61 |
| >SCJFRZ3C01G05 | | lysosomal alpha-mannosidase-like | 14 | | 7 | | 75.6865 | | NE-0 | | NE-21 | | 8075.897 | 11701.38 | 9044.214 | 6380.334 |
| >SCAGLR1043H07 | | carrier protein | 22 | | 11 | | 143.2347 | | NE-0 | | E-0 | | 1018.081 | 13790.63 | 1812.751 | 7525.776 |
| >SCCCLR2002D08 | | translocon tic40 | 12 | | 5 | | 67.0322 | | E-21 | | NE-21 | | 7433.326 | 1630.517 | 7591.528 | 890.002 |
| >SCSGFL1078A06.b | | endoplasmin precursor | 86 | | 53 | | 678.8232 | | E-21 | | NE-21 | | 182447.8 | 177120.2 | 188442.4 | 96714.32 |
| >SCCCCL7C02A04 | | cupin family expressed | 16 | | 10 | | 160.3824 | | E-21 | | NE-21 | | 113367.5 | 3069.773 | 243333 | 1684.903 |
| >SCCCLB1024G12 | | glucosidase 2 subunit beta-like isoform 1 | 4 | | 2 | | 20.6856 | | E-0 | | E-21 | | 6336.318 | 5499.077 | 1859.324 | 3032.711 |
| >SCCCLR2001F05 | | delta-1-pyrroline-5-carboxylate synthetase | 37 | | 24 | | 233.9558 | | NE-0 | | E-0 | | 22056.56 | 42155.74 | 25802.42 | 23283.83 |
| >SCSGFL5C07C07 | | replication licensing factor mcm7 homologue | 64 | | 41 | | 393.213 | | E-21 | | NE-21 | | 56902.78 | 51006.17 | 60840.62 | 28401.23 |
| >SCCCLB1004D05 | | nadh-cytochrome b5 reductase-like protein | 30 | | 21 | | 236.5468 | | NE-0 | | NE-21 | | 45656.56 | 67372.69 | 38693.61 | 37599.27 |
| >SCBFLR1083H11 | | rae1-like protein | 12 | | 6 | | 80.1728 | | NE-0 | | E-21 | | 3672.048 | 18514.62 | 1924.544 | 10334.28 |
| >SCJLLR1107E04 | | snrk1-interacting protein 1 | 16 | | 10 | | 113.3718 | | E-21 | | NE-21 | | 16859.42 | 9986.667 | 26116.58 | 5589.197 |
| >SCCCCL3004H02.b | | proliferation-associated protein 2g4 | 23 | | 13 | | 177.0052 | | NE-0 | | E-21 | | 12233.23 | 50275.86 | 11581.64 | 28215.31 |
| >SCACLR2007C03 | | guanine nucleotide-binding protein beta subunit... | 34 | | 23 | | 336.4325 | | NE-0 | | NE-21 | | 189353.7 | 275623.8 | 168088.9 | 154703 |
| >SCCCLR2C02D02 | | macrophage migration inhibitory factor | 7 | | 6 | | 67.7377 | | E-21 | | NE-21 | | 36436.77 | 21238.68 | 72445 | 11958.45 |
| >SCJLRT2049G08 | | copa3_orysj ame: full=coatomer subunit alpha-3 | 43 | | 26 | | 280.1992 | | E-21 | | NE-21 | | 158485.4 | 30481.7 | 182556.2 | 17215.88 |
| >SCQSST1035B11 | | calreticulin-3 precursor | 5 | | 2 | | 23.8042 | | NE-0 | | E-21 | | 362.8173 | 12433.49 | 314.0735 | 7023.24 |
| >SCCCRZ1004G02 | | acetylornithine deacetylase | 34 | | 22 | | 252.0388 | | E-0 | | NE-21 | | 78344.28 | 47826.29 | 60535.73 | 27049.28 |
| >SCEPRZ1009B12 | | triosephosphate cytosolic | 79 | | 47 | | 815.0929 | | E-21 | | NE-21 | | 1032059 | 1010360 | 1046807 | 573461.8 |
| >SCEZLB1007F07 | | arginyl-trna synthetase | 34 | | 24 | | 222.2821 | | NE-0 | | E-21 | | 39850.79 | 96330.06 | 17782.52 | 54921.04 |
| >SCEQAM2039A03 | | monocopper oxidase-like protein sku5-like | 16 | | 10 | | 101.2518 | | NE-0 | | E-21 | | 16407.23 | 18547.62 | 10493.52 | 10621.8 |
| >SCCCCL4009B07 | | villin-2-like isoform 1 | 13 | | 8 | | 71.4903 | | NE-0 | | E-0 | | 3632.479 | 11624.74 | 5027.946 | 6673.302 |
| >SCSGLR1081B03 | | reticulon-4-interacting protein 1 | 11 | | 6 | | 78.3725 | | E-21 | | NE-21 | | 13010.34 | 9173.099 | 17908.92 | 5279.217 |
| **Table S1.** Continued |  | | |  | |  | |  | |  | |  |  |  |  |  |
| >SCAGLR1043D06 | | methyl binding domain106 | 33 | | 23 | | 327.3972 | | E-21 | | NE-21 | | 126800.5 | 77970.33 | 219334.1 | 45015.41 |
| >SCJLLR1011H01 | | msi type nucleosome chromatin assembly factor c | 11 | | 5 | | 57.765 | | E-0 | | E-21 | | 11621.21 | 9131.821 | 5100.079 | 5319.851 |
| >SCEZHR1084E11 | | peroxidase 1 precursor | 196 | | 116 | | 1500.1908 | | NE-0 | | E-21 | | 214332.2 | 762890.7 | 196183.6 | 445562.3 |
| >SCVPFL1134D05 | | riboflavin biosynthesis protein riba | 5 | | 3 | | 34.3912 | | NE-0 | | E-0 | | 1306.805 | 4532.236 | 1815.342 | 2651.968 |
| >SCCCRT2C04H02 | | enolase 1 | 64 | | 30 | | 715.8054 | | NE-0 | | E-21 | | 501699.8 | 896197.1 | 496830.8 | 525112.2 |
| >SCCCRT1004F06 | | aminotransferase y4ub | 27 | | 20 | | 161.2251 | | E-0 | | NE-21 | | 109953.2 | 28780.2 | 45769.83 | 16907.45 |
| >SCRFLR2034H11 | | heterogeneous nuclear ribonucleoprotein a3... | 51 | | 30 | | 361.9446 | | NE-0 | | NE-21 | | 59956.61 | 64495.27 | 62233.24 | 38095.87 |
| >SCCCLR2004H11 | | t-complex protein 1 theta chain | 247 | | 167 | | 1796.2058 | | NE-0 | | NE-21 | | 367881.4 | 437040.1 | 301286.9 | 258472.7 |
| >SCBGLB2072H09 | | cw-type zinc finger family expressed | 3 | | 2 | | 12.2557 | | E-21 | | NE-21 | | 827.7621 | 383.8954 | 2321.253 | 227.5287 |
| >SCRFLR1034B02 | | dead-box atp-dependent rna helicase 56-like | 69 | | 37 | | 539.1461 | | E-0 | | NE-21 | | 127907.6 | 69445.71 | 106213.7 | 41218.56 |
| >SCQSSB1059F03 | | ribose-5-phosphate isomerase | 13 | | 10 | | 94.1233 | | NE-0 | | E-21 | | 11375.72 | 18843.33 | 1980.486 | 11203.3 |
| >SCCCCL5071D04 | | tonoplast intrinsic protein | 1 | | 1 | | 8.0538 | | E-21 | | NE-21 | | 2071.329 | 153.1161 | 11229.84 | 91.78444 |
| >SCJFRZ2027B01 | | amp-binding protein | 30 | | 18 | | 207.5364 | | NE-0 | | E-21 | | 10054.19 | 48292.36 | 7906.608 | 29095.54 |
| >SCRLLR1059F06 | | mucin-like protein | 6 | | 3 | | 39.1242 | | NE-0 | | E-21 | | 4584.2 | 6730.207 | 1703.159 | 4077.27 |
| >SCBFRT3093E07 | | leucoanthocyanidin dioxygenase 1 | 8 | | 6 | | 53.157 | | NE-0 | | NE-21 | | 23543.82 | 34670.29 | 34361.15 | 21052.83 |
| >SCCCCL4003A03 | | tetratricopeptide repeat protein 1-like | 17 | | 10 | | 105.7923 | | NE-0 | | E-21 | | 3359.192 | 7391.617 | 1726.573 | 4501.017 |
| >SCVPRZ2038H03 | | proteasome alpha subunit | 273 | | 169 | | 2233.0416 | | NE-0 | | NE-21 | | 586563.6 | 907677.7 | 611121.1 | 558296.9 |
| >SCACFL5027E11 | | pyruvate kinase isozyme chloroplast precursor | 299 | | 196 | | 2363.9015 | | NE-0 | | NE-21 | | 719697 | 1035781 | 656631.2 | 642341.6 |
| >SCJFSB1012F11 | | phospholipase d alpha 1 | 64 | | 45 | | 475.6482 | | NE-0 | | E-0 | | 115868.5 | 354727 | 146014.1 | 222421.1 |
| >SCSFAD1068F07 | | beta-hexosaminidase beta chain precursor | 3 | | 3 | | 27.0589 | | E-0 | | NE-21 | | 10407.07 | 8224.332 | 9023.819 | 5172.37 |
| >SCJFRZ2026E06 | | pura1_sorbi | 11 | | 6 | | 90.9944 | | NE-0 | | E-21 | | 27358.06 | 39704.79 | 16044.91 | 25037.24 |
| >SCRLRZ3042G05 | | calmodulin-binding protein | 11 | | 6 | | 66.7211 | | E-21 | | NE-21 | | 4193.471 | 3806.943 | 12318.58 | 2400.631 |
| >SCCCLR1066A08 | | rh2 protein | 25 | | 17 | | 199.6764 | | E-21 | | NE-21 | | 394868.4 | 62804.44 | 686279.4 | 39640.3 |
| >SCAGRT2041G07 | | dihydrolipoamide s-acetyltransferase | 55 | | 38 | | 368.609 | | NE-0 | | E-0 | | 51565.38 | 109660.9 | 72459.27 | 69421.21 |
| >SCVPLR1028E03 | | pci domain containing protein | 47 | | 25 | | 360.5509 | | NE-0 | | E-21 | | 79730.7 | 108209.5 | 41642.19 | 68596.62 |
| >SCEQRT2090H11 | | transducin wd-40 repeat | 63 | | 35 | | 403.7021 | | E-0 | | NE-21 | | 65479.68 | 49014.09 | 54293.91 | 31103.67 |
| >SCCCSB1002A08 | | outer mitochondrial membrane protein porin | 71 | | 41 | | 579.0222 | | E-21 | | NE-21 | | 110848.2 | 114230.8 | 143874.5 | 72741 |
| **Table S1.** Continued |  | | |  | |  | |  | |  | |  |  |  |  |  |
| >SCCCCL5002C02 | | isopenicillin n epimerase | 13 | | 8 | | 90.5812 | | E-0 | | NE-21 | | 5782.331 | 883.2609 | 2924.96 | 564.0289 |
| >SCACLR1036F01 | | alpha-l-fucosidase 2-like | 10 | | 6 | | 62.5603 | | E-21 | | NE-21 | | 7214.004 | 3971.947 | 8211.804 | 2543.701 |
| >SCCCCL5004D04 | | cinnamoyl reductase-like 2a | 33 | | 20 | | 256.4894 | | E-0 | | NE-21 | | 72557.91 | 64074.58 | 60028.31 | 41213.78 |
| >SCSFRT2069D09 | | pyrophosphate-dependent phosphofructokinase... | 66 | | 29 | | 520.8001 | | NE-0 | | NE-21 | | 102853.9 | 122198.1 | 93271.21 | 78676.03 |
| >SCEPLB1043H10 | | rrna 2 -o-methyltransferase fibrillarin 1-like | 11 | | 10 | | 90.88 | | E-21 | | NE-21 | | 200664.5 | 23208.57 | 276916.8 | 14947.77 |
| >SCCCCL4009H11 | | af236369_1 prohibitin | 21 | | 16 | | 198.4113 | | E-21 | | NE-21 | | 108187.7 | 49983.28 | 130494.3 | 32217.84 |
| >SCSGHR1071C01 | | sumo-activating enzyme subunit 2-like | 20 | | 15 | | 125.5071 | | E-21 | | NE-21 | | 39974.71 | 19053.49 | 48161.15 | 12304.71 |
| >SCCCCL6002B05 | | nitrilase 2 | 10 | | 8 | | 83.8845 | | E-0 | | NE-21 | | 53769.3 | 22726.34 | 20925.3 | 14835.75 |
| >SCQGST1034A08 | | 14-3-3-like protein | 100 | | 68 | | 1000.6875 | | NE-0 | | E-21 | | 714481.4 | 1481639 | 709083.7 | 967775.7 |
| >SCCCCL3001A01 | | 2-hydroxyacyl- lyase-like | 8 | | 5 | | 64.4219 | | NE-0 | | E-0 | | 4161.339 | 10026.22 | 7259.066 | 6554.907 |
| >SCEPSB1128E09 | | nad-dependent malic enzyme 62 kda... | 44 | | 23 | | 268.799 | | NE-0 | | E-21 | | 36905.19 | 61815.89 | 25756.22 | 40439.55 |
| >SCVPCL6046A06 | | seed maturation protein pm41 | 17 | | 13 | | 116.4626 | | E-21 | | NE-21 | | 27935.78 | 13183.74 | 52056.28 | 8659.311 |
| >SCQSRT1035C02 | | dna-binding protein | 54 | | 42 | | 353.1174 | | NE-0 | | NE-21 | | 108387 | 131469.5 | 98393.24 | 86721.19 |
| >SCRLAM1013H08 | | u5 small nuclear ribonucleoprotein component | 34 | | 20 | | 205.9656 | | NE-0 | | E-0 | | 28909.73 | 57914.67 | 38128.38 | 38460.22 |
| >SCEQLB1065B12 | | fk506-binding protein 2-1 precursor | 10 | | 9 | | 61.412 | | E-21 | | NE-21 | | 17904.27 | 9733.813 | 22668.5 | 6490.266 |
| >SCMCLR1123B01 | | fasciclin-like arabinogalactan protein 10... | 26 | | 18 | | 185.5833 | | E-0 | | NE-21 | | 39403.8 | 16558.53 | 30468.35 | 11071.8 |
| >SCJFRZ2027B03 | | nadph-thioredoxin reductase | 8 | | 5 | | 55.989 | | NE-0 | | E-0 | | 2054.639 | 12256.54 | 5035.935 | 8281.633 |
| >SCEPRZ1010H09 | | dynamin-related protein 1a | 35 | | 18 | | 205.1526 | | E-21 | | NE-21 | | 62602.3 | 60227.79 | 66463.19 | 40847.37 |
| >SCACSB1038A04 | | rhicadhesin receptor precursor | 11 | | 9 | | 138.4831 | | NE-0 | | E-21 | | 116855 | 447323.7 | 77191.99 | 303575.4 |
| >SCEQLB1066D03 | | syntaxin 72 | 12 | | 9 | | 77.3964 | | E-21 | | NE-21 | | 22997.97 | 20547.07 | 27027.96 | 14004.1 |
| >SCEQRT1026G11 | | leucine aminopeptidase | 77 | | 44 | | 686.4492 | | E-21 | | NE-21 | | 164992.3 | 174897.2 | 177630.1 | 119770.9 |
| >SCEQAM1035F11 | | calcium ion binding | 10 | | 6 | | 64.6461 | | E-0 | | NE-21 | | 14615.28 | 2805.966 | 13825.92 | 1926.642 |
| >SCRLFL4028D02 | | na+ h+ antiporter | 3 | | 1 | | 17.7953 | | NE-0 | | E-0 | | 10111.08 | 80009.23 | 19986.98 | 54939.98 |
| >SCCCCL3002B06.b | | isochorismate synthase 1 | 28 | | 20 | | 229.2697 | | E-21 | | NE-21 | | 104275 | 67205.01 | 127662.5 | 46157.56 |
| >SCQGSB1144A01 | | PREDICTED: uncharacterized protein... | 32 | | 18 | | 185.4905 | | E-21 | | NE-21 | | 12051.74 | 9310.524 | 20785.99 | 6417.587 |
| >SCCCCL4011E04 | | pairing protein meu13-like | 4 | | 2 | | 23.1364 | | E-0 | | NE-21 | | 1388.743 | 344.6668 | 909.4002 | 237.9263 |
| >SCUTHR1065A04 | | nucleic acid binding protein | 60 | | 35 | | 352.7818 | | E-0 | | NE-21 | | 40129.63 | 20315.44 | 24435.38 | 14107.74 |
| **Table S1.** Continued |  | | |  | |  | |  | |  | |  |  |  |  |  |
| >SCBGAM1092H06 | | carboxypeptidase d | 13 | | 7 | | 121.1111 | | E-21 | | NE-21 | | 10577.64 | 12100.08 | 24351.72 | 8408.027 |
| >SCEQRT1025H04 | | heat-shock protein 101 | 36 | | 12 | | 207.1056 | | E-21 | | NE-21 | | 5652.596 | 2414.997 | 6680.752 | 1687.738 |
| >SCBFRZ2017C11 | | per1_maize | 15 | | 9 | | 131.0551 | | E-0 | | NE-21 | | 15548.62 | 10426.71 | 12847.17 | 7289.34 |
| >SCCCFL3002F10 | | dna replication licensing factor expressed | 132 | | 56 | | 791.317 | | E-0 | | NE-21 | | 170456 | 68698.14 | 160615.6 | 48085.72 |
| >SCEQRT2092A09 | | peroxisomal targeting signal 1 receptor | 57 | | 37 | | 363.565 | | NE-0 | | E-0 | | 42391.23 | 152763 | 49291.21 | 106989.9 |
| >SCJLLR1033F07 | | 2-cys peroxiredoxin bas1 | 17 | | 12 | | 144.5669 | | E-0 | | NE-21 | | 95273.67 | 87182.54 | 89350.56 | 61068.46 |
| >SCRLLR1016F07 | | alpha- -galacturonosyltransferase 1-like | 72 | | 44 | | 585.3258 | | NE-0 | | E-0 | | 103343.5 | 325892.2 | 135569.6 | 228666 |
| >SCCCLR1C01E07 | | histidinol-phosphate aminotransferase | 7 | | 4 | | 46.9907 | | NE-0 | | E-21 | | 5289.503 | 5866.128 | 3954.729 | 4143.786 |
| >SCBGHR1061F05 | | phosphoribosylamine--glycine ligase | 14 | | 7 | | 97.2493 | | NE-0 | | E-21 | | 22293.84 | 58495.7 | 20677.24 | 41365.1 |
| >SCCCLR2001D01 | | histone h4 | 128 | | 79 | | 1083.4304 | | E-0 | | NE-21 | | 753854.4 | 393670.1 | 634370.3 | 278821.5 |
| >SCQGLR1086B12 | | stress responsive protein | 20 | | 12 | | 134.116 | | NE-0 | | NE-21 | | 5882.284 | 6563.433 | 4950.185 | 4649.453 |
| >SCEZLB1012H08 | | actin-like protein 6a | 16 | | 12 | | 91.8375 | | E-0 | | NE-21 | | 17344.37 | 7081.566 | 10237.87 | 5077.334 |
| >SCVPRZ2041G12 | | indole-3-acetic acid-amido synthetase | 56 | | 36 | | 397.5192 | | E-0 | | NE-21 | | 143433.7 | 42131.11 | 55305.94 | 30211.17 |
| >SCCCLR2003G06 | | thaumatin-like cytokinin-binding expressed | 20 | | 12 | | 150.1229 | | NE-0 | | E-0 | | 49037.01 | 75202.2 | 71884.94 | 54048.66 |
| >SCACHR1036C12 | | hypersensitive-induced response protein | 39 | | 21 | | 246.3901 | | E-21 | | NE-21 | | 39537.28 | 42816.8 | 48480.11 | 30835.02 |
| >SCRLAM1012D07 | | iq calmodulin-binding motif family protein | 18 | | 15 | | 128.2619 | | NE-0 | | E-0 | | 33574.67 | 69188.61 | 46288.84 | 49901.46 |
| >SCEQRT1033B09 | | 2-oxoglutarate e1 subunit | 30 | | 13 | | 221.0685 | | NE-0 | | E-21 | | 17409.72 | 26735.4 | 11512.32 | 19347.83 |
| >SCSGAM1095C10 | | prs4_orysj | 32 | | 13 | | 261.372 | | E-0 | | NE-21 | | 74892.45 | 43254.84 | 66431.65 | 31363.46 |
| >SCACLR2029H09 | | xylogen protein 1 | 11 | | 9 | | 108.9826 | | E-0 | | NE-21 | | 107769.1 | 51824.15 | 98386.1 | 37737.26 |
| >SCRURT2008D11 | | glycosyl hydrolase family 3 n terminal domain... | 77 | | 45 | | 543.3879 | | NE-0 | | E-0 | | 41165.65 | 116845.4 | 56625.39 | 85102.8 |
| >SCJFST1012D03 | | mannosylglycoprotein endo-beta-mannosidase... | 11 | | 6 | | 55.7308 | | NE-0 | | E-21 | | 431.8701 | 9243.849 | 211.3116 | 6743.537 |
| >SCCCLR2002B03 | | dynamin-like protein | 2 | | 2 | | 18.0477 | | E-21 | | E-0 | | 866.8606 | 1827.795 | 3889.421 | 1340.13 |
| >SCJFRT1012A02 | | oep24_orysi | 14 | | 8 | | 85.5001 | | E-0 | | E-21 | | 46041.86 | 21283.05 | 4751.748 | 15610.14 |
| >SCRFFL5039B12 | | ankyrin protein | 30 | | 16 | | 181.3596 | | E-0 | | NE-21 | | 52934.78 | 44062.91 | 48696.31 | 32464.8 |
| >SCQSLR1061C03 | | small nuclear ribonucleoprotein e-like | 31 | | 16 | | 231.1935 | | NE-0 | | E-0 | | 37800.26 | 66301.91 | 47839.37 | 48883.83 |
| >SCMCLR1053H06 | | lysyl-trna synthetase | 22 | | 14 | | 162.262 | | NE-0 | | E-0 | | 14696.43 | 44147.86 | 37540.87 | 32608.06 |
| >SCCCLR2001E04 | | huntingtin-interacting protein k-like | 9 | | 5 | | 88.5859 | | NE-0 | | E-21 | | 98325.29 | 225374.2 | 52908.66 | 167026.4 |
| **Table S1.** Continued |  | | |  | |  | |  | |  | |  |  |  |  |  |
| >SCCCLB1004H08 | | adenosine kinase | 25 | | 18 | | 244.7617 | | NE-0 | | E-0 | | 95256.16 | 173290.5 | 101767 | 128862.4 |
| >SCCCCL4011E03 | | toc75_orysj | 23 | | 15 | | 128.3332 | | E-0 | | NE-21 | | 20962.34 | 10499.95 | 16564.13 | 7828.337 |
| >SCQSLR1061B01 | | fibroblast growth factor 2-interacting factor | 22 | | 16 | | 123.2497 | | NE-0 | | E-0 | | 19052.43 | 32498.6 | 21985.39 | 24289.97 |
| >SCJLLR1054A06 | | 1-aminocyclopropanE-0-carboxylate oxidase 1 | 22 | | 19 | | 157.5474 | | NE-0 | | E-21 | | 33000.39 | 126846.2 | 15696.77 | 94853.14 |
| >SCMCCL6053E09 | | tryptophan synthase beta-subunit | 12 | | 8 | | 87.0141 | | NE-0 | | E-21 | | 6731.032 | 19031.68 | 6367.3 | 14236.97 |
| >SCQGLB1027C07 | | vip3 protein | 10 | | 7 | | 69.5008 | | E-21 | | NE-21 | | 32876.58 | 23823.74 | 35648.45 | 17941.93 |
| >SCCCLR1C01G03 | | adp-ribosylation expressed | 34 | | 20 | | 225.708 | | E-21 | | NE-21 | | 88074.64 | 68799.58 | 95959.3 | 51831.39 |
| >SCJLRT1016A04 | | plastid-lipid-associated protein 2 | 5 | | 1 | | 28.0025 | | E-0 | | NE-21 | | 3018.852 | 2266.618 | 2269.277 | 1708.314 |
| >SCRFAM2071E02 | | glucan endo- -beta-glucosidase precursor | 29 | | 21 | | 174.1288 | | E-21 | | E-0 | | 25478.32 | 42177.08 | 86602.12 | 31828.41 |
| >SCBGFL4053G08 | | phosphoenolpyruvate carboxylase | 165 | | 104 | | 1208.1647 | | E-0 | | NE-21 | | 382090.9 | 195893.1 | 344223.7 | 148349.5 |
| >SCCCRZ2002H06 | | aspartic proteinase oryzasin-1 precursor | 41 | | 28 | | 336.1406 | | NE-0 | | E-21 | | 114097.8 | 122595.8 | 81073.1 | 94389.26 |
| >SCCCLB1002D05 | | loc100286338 precursor | 12 | | 7 | | 84.6403 | | E-0 | | NE-21 | | 16799.22 | 13064.34 | 13944.44 | 10062.5 |
| >SCVPLR2027C10 | | tcp-1 cpn60 chaperonin family protein | 51 | | 36 | | 376.2577 | | E-0 | | NE-21 | | 128967 | 97807.57 | 115546.2 | 75929.36 |
| >SCSFLR2009H09 | | cytosolic chaperonin delta-subunit | 144 | | 74 | | 1169.3742 | | NE-0 | | E-21 | | 200616.2 | 265758.5 | 200316.4 | 206345.3 |
| >SCSGLR1045G11 | | cml7_orysj | 17 | | 10 | | 131.4453 | | E-0 | | NE-21 | | 26557.11 | 12359.01 | 17553.04 | 9633.523 |
| >SCRFAD1117F03 | | eukaryotic translation initiation factor 2 beta... | 494 | | 267 | | 3429.5305 | | NE-0 | | NE-21 | | 510099.4 | 524077.4 | 422520.6 | 410523.6 |
| >SCCCLB1023G10 | | armadillo beta-catenin-like repeat family protein | 17 | | 6 | | 112.9474 | | E-0 | | NE-21 | | 11340.03 | 2149.754 | 4585.333 | 1686.436 |
| >SCQGST3123C08 | | pectin methylesterase | 3 | | 2 | | 16.4109 | | NE-0 | | E-21 | | 331.1384 | 722.1381 | 190.716 | 569.3307 |
| >SCJLLR1054F05 | | dnak-type molecular chaperone | 111 | | 36 | | 1063.5405 | | NE-0 | | E-0 | | 190782.3 | 354332.4 | 206237.2 | 279669.8 |
| >SCCCLB1001H09 | | methyl-binding domain protein mbd111 | 46 | | 28 | | 311.7707 | | E-0 | | NE-21 | | 81090.59 | 59041.97 | 48025.76 | 46623.55 |
| >SCCCST1006A11 | | glycine dehydrogenase | 19 | | 11 | | 108.9144 | | E-21 | | NE-21 | | 20298 | 9888.559 | 24266.31 | 7823.209 |
| >SCCCLR1C02F07 | | inositol-3-phosphate synthase | 26 | | 12 | | 205.9797 | | E-21 | | NE-21 | | 26338.82 | 2543.435 | 63197.93 | 2028.232 |
| >SCCCCL3002A02.b | | chloride intracellular channel 6 | 23 | | 17 | | 250.8429 | | E-0 | | NE-21 | | 189823 | 90946.06 | 152418.9 | 72602.59 |
| >SCCCCL5003C11 | | glutathione s-transferase 4 | 216 | | 141 | | 1804.5944 | | NE-0 | | E-21 | | 1048644 | 1076003 | 726802.7 | 861353.4 |
| >SCUTSD2028F03 | | glyceraldehyde-3-phosphate partial | 99 | | 43 | | 1106.989 | | E-21 | | E-0 | | 795085.4 | 1005330 | 1274650 | 807328.3 |
| >SCSGAM1094A08 | | ribonucleotide reductase | 25 | | 13 | | 147.9039 | | E-21 | | NE-21 | | 8595.877 | 5199.716 | 11917.39 | 4187.315 |
| >SCCCLR1C04F06 | | diphosphomevalonate decarboxylase | 8 | | 6 | | 50.6094 | | E-21 | | NE-21 | | 4090.957 | 854.0574 | 15357.31 | 688.1318 |
| **Table S1.** Continued |  | | |  | |  | |  | |  | |  |  |  |  |  |
| >SCCCRZ1003F10 | | c2 domain containing protein | 24 | | 16 | | 167.6721 | | NE-0 | | E-0 | | 57512.21 | 89918.59 | 68861.01 | 72546.75 |
| >SCPRRT3026E05 | | aspartyl-trna synthetase | 31 | | 19 | | 207.9798 | | E-21 | | NE-21 | | 66556.89 | 75390.55 | 78503.35 | 61126.05 |
| >SCACLR1057G08 | | activator of 90 kda heat shock protein atpase | 23 | | 13 | | 169.4497 | | NE-0 | | E-21 | | 27748.06 | 59881.27 | 16104.42 | 48567.3 |
| >SCSFRT2072C08 | | ml domain protein | 6 | | 5 | | 49.6795 | | NE-0 | | E-0 | | 12901.25 | 79734.26 | 13170.21 | 64924.73 |
| >SCEZRZ1012F05 | | methylenetetrahydrofolate expressed | 30 | | 18 | | 201.373 | | E-0 | | E-21 | | 34282.9 | 25586.16 | 20658.54 | 20868.39 |
| >SCCCCL7038G03 | | methionyl-trna synthetase | 34 | | 22 | | 226.1558 | | NE-0 | | NE-21 | | 37944.78 | 39824.95 | 38791.98 | 32502.11 |
| >SCEZRZ1014D09 | | coatmer beta subunit (beta-coat protein)... | 32 | | 13 | | 190.1765 | | E-21 | | NE-21 | | 11773.07 | 12392.53 | 15811.08 | 10119.93 |
| >SCCCLR1024C10 | | endothelial differentiation-related factor 1 | 3 | | 3 | | 37.3708 | | E-21 | | E-0 | | 2180.491 | 4481.767 | 10267.46 | 3674.765 |
| >SCUTSD2025B07 | | elongation factor 1-alpha | 286 | | 194 | | 2666.7892 | | E-21 | | NE-21 | | 1633347 | 1724345 | 1753848 | 1414859 |
| >SCRURT2011B01 | | 2-dehydro-3-deoxyphosphooctonate expressed | 15 | | 10 | | 93.936 | | NE-0 | | E-21 | | 17454.58 | 32275.36 | 17217.97 | 26491.88 |
| >SCCCCL3080C05 | | aspartyl expressed | 14 | | 6 | | 84.7793 | | NE-0 | | NE-21 | | 6924.249 | 8368.89 | 7207.115 | 6869.8 |
| >SCBFLR1083E03 | | nucleoside diphosphate kinase i | 31 | | 20 | | 275.33 | | E-21 | | E-0 | | 121169.7 | 147116.7 | 168235.8 | 121283.5 |
| >SCCCFL4092E06 | | pyrophosphate--fructose 6-phosphate... | 58 | | 42 | | 467.9343 | | NE-0 | | E-21 | | 339588.9 | 518421.1 | 199443.6 | 427696 |
| >SCCCFL1001E01 | | loc100283433 precursor | 10 | | 3 | | 52.9694 | | E-0 | | NE-21 | | 3312.04 | 1924.839 | 3205.94 | 1588.821 |
| >SCEQLR1050F11 | | hypothetical protein SORBIDRAFT_03g004340... | 1945 | | 1002 | | 12700.9317 | | NE-0 | | E-21 | | 2054525 | 2462972 | 1988846 | 2035515 |
| >SCCCLR2003A04 | | allene oxide cyclase 4 | 23 | | 9 | | 168.2744 | | NE-0 | | E-21 | | 10284.17 | 13434.27 | 2940.445 | 11118.03 |
| >SCCCCL4017C01 | | syh_orysj | 15 | | 9 | | 77.4613 | | NE-0 | | E-21 | | 8457.308 | 10406.76 | 4846.561 | 8665.216 |
| >SCQGLR1019E07 | | e3 ubiquitin-protein ligase upl1-like | 19 | | 13 | | 108.2425 | | NE-0 | | E-0 | | 6700.948 | 35902.79 | 6728.296 | 29900.07 |
| >SCVPLR2019B03 | | polygalacturonase inhibitor 1 precursor | 16 | | 11 | | 123.5409 | | NE-0 | | E-0 | | 24223.89 | 43446.67 | 30967.68 | 36182.95 |
| >SCEQRT1028A02 | | gme2_orysj | 23 | | 10 | | 178.988 | | NE-0 | | E-0 | | 1736.575 | 31515.72 | 3887.324 | 26248.75 |
| >SCRFAM1026D04 | | ran gtpase activating protein | 48 | | 23 | | 298.9883 | | E-0 | | E-21 | | 52795.85 | 28585.23 | 20838.83 | 23908.47 |
| >SCJLFL1053D04 | | nodal modulator 1-like | 15 | | 8 | | 83.6124 | | E-0 | | E-21 | | 8017.094 | 7347.709 | 6063.324 | 6145.961 |
| >SCCCLR1C03G09 | | chalcone flavanone isomerase1 | 4 | | 3 | | 23.3373 | | NE-0 | | E-0 | | 29.7517 | 1388.701 | 33.13002 | 1162.936 |
| >P00330 | | ADH1_YEAST Alcohol dehydrogenase 1... | 25 | | 20 | | 218.6479 | | E-21 | | NE-21 | | 54015.14 | 42137.33 | 374341 | 35565.13 |
| >SCQSAM2101B07 | | splicing arginine serine-rich 7 | 95 | | 53 | | 661.8774 | | E-0 | | NE-21 | | 116706.1 | 110511.7 | 110693.7 | 93791.5 |
| >SCCCRT2002B07 | | trna- expressed | 7 | | 3 | | 35.1372 | | E-21 | | NE-21 | | 2426.741 | 249.3176 | 3223.02 | 212.0904 |
| >SCMCRZ3065B07 | | xylulose kinase | 18 | | 8 | | 99.4766 | | NE-0 | | E-0 | | 1970.062 | 16202.37 | 6282.198 | 13827.22 |
| **Table S1.** Continued |  | | |  | |  | |  | |  | |  |  |  |  |  |
| >SCVPFL3040H07 | | salt tolerance expressed | 33 | | 28 | | 317.0089 | | E-0 | | NE-21 | | 251460.1 | 240998.8 | 222168.7 | 205695.5 |
| >SCVPCL6041E09 | | leukotriene a-4 hydrolase | 30 | | 23 | | 255.7269 | | E-0 | | NE-21 | | 89128.81 | 34980.55 | 72535.73 | 29935.91 |
| >SCSFFL3093H05 | | sister-chromatid cohesion protein | 12 | | 7 | | 73.0126 | | E-0 | | NE-21 | | 8699.827 | 1313.395 | 5002.368 | 1125.531 |
| >SCVPRZ2038C12 | | polyubiquitin 14 | 26 | | 5 | | 227.1644 | | E-0 | | NE-21 | | 44891.4 | 26439.86 | 33323.55 | 22667.63 |
| >SCRFRT3057C02 | | translational initiation factor eif-4a | 115 | | 46 | | 1039.4061 | | NE-0 | | E-21 | | 248597.1 | 286578.9 | 228670.3 | 245809.3 |
| >SCCCST2004E07 | | gras family transcription factor containing... | 10 | | 5 | | 63.4382 | | E-0 | | E-21 | | 6601.26 | 1391.955 | 609.9562 | 1193.942 |
| >SCCCLR1024G01 | | low quality protein: alanyl-trna synthetase-like | 106 | | 56 | | 699.4997 | | E-0 | | NE-21 | | 97401.23 | 85609.82 | 85387.54 | 73604.27 |
| >SCCCLR2001H06 | | transcriptional coactivator-like | 17 | | 9 | | 91.7628 | | E-21 | | NE-21 | | 6933.847 | 3443.456 | 13172.12 | 2960.566 |
| >SCRFLR1012E03 | | isopentenyl pyrophosphate:dimethyllallyl... | 4 | | 3 | | 23.4073 | | E-0 | | NE-21 | | 1893.905 | 701.8276 | 1202.537 | 603.7318 |
| >SCCCLR1C01A11 | | tyrosyl-trna synthetase | 16 | | 10 | | 118.6678 | | NE-0 | | E-0 | | 16635.2 | 28118.55 | 25462.83 | 24199.02 |
| >SCVPLB1015B03 | | fructose-bisphosphate aldolase | 105 | | 72 | | 1061.5568 | | NE-0 | | E-0 | | 870112.8 | 1353397 | 1225315 | 1165637 |
| >SCSGRT2062E10 | | transformer-sr ribonucleoprotein | 5 | | 3 | | 29.6866 | | E-21 | | NE-21 | | 1767.69 | 670.431 | 2956.072 | 578.0975 |
| >SCVPCL6041A03 | | cysteinyl-trna expressed | 10 | | 8 | | 68.0316 | | E-21 | | NE-21 | | 2621.543 | 1407.225 | 8597.425 | 1222.321 |
| >SCEPCL6018F02 | | glycyl-trna synthetase | 75 | | 52 | | 478.2277 | | E-0 | | NE-21 | | 88030.32 | 86624.65 | 85253.16 | 75263.2 |
| >SCBGLR1117A05 | | ran-binding protein 1 | 22 | | 16 | | 230.7805 | | E-21 | | NE-21 | | 59761.54 | 34275.68 | 60293.37 | 29812.93 |
| >SCRFRT3060F01 | | actin-depolymerizing factor 3 | 15 | | 9 | | 121.8193 | | E-21 | | NE-21 | | 23773.79 | 5996.99 | 33279.94 | 5233.604 |
| >SCAGLR1021D02 | | ubiquitin-conjugating enzyme e2 n | 49 | | 30 | | 396.7887 | | E-21 | | NE-21 | | 145520.4 | 65868.3 | 191691.8 | 57760.24 |
| >SCEPAM1015B01 | | tryptophanyl-trna synthetase | 22 | | 20 | | 134.1992 | | E-0 | | NE-21 | | 39691.26 | 18554.88 | 26792.28 | 16328.06 |
| >SCCCLR1024C11 | | stromal 70 kda heat shock-related protein | 52 | | 23 | | 365.3031 | | E-0 | | NE-21 | | 96371.73 | 56035.74 | 75716.72 | 49324.29 |
| >SCCCRZ1001D04 | | cytoplasmic 2 | 56 | | 41 | | 446.465 | | E-0 | | NE-21 | | 249919.9 | 134091.5 | 188013.3 | 118191.9 |
| >SCACAM1072H07 | | gcn5-related n- expressed | 7 | | 5 | | 51.2557 | | NE-0 | | E-0 | | 3330.35 | 4730.989 | 3565.234 | 4171.443 |
| >SCCCCL3120B01 | | fructokinase-2 | 42 | | 31 | | 443.8685 | | NE-0 | | E-0 | | 245682.9 | 463442 | 359332.8 | 408938 |
| >SCCCCL4011G05 | | rna-binding protein luc7-like 2 | 78 | | 46 | | 454.437 | | E-0 | | NE-21 | | 37891.93 | 19107.31 | 36265.27 | 16878.27 |
| >SCJLFL3019C04 | | pmm_orysj | 9 | | 6 | | 78.0818 | | NE-0 | | E-21 | | 2572.568 | 11685.43 | 2452.054 | 10335.83 |
| >SCCCLR1024D03 | | malate cytoplasmic | 108 | | 79 | | 1109.0054 | | E-21 | | NE-21 | | 719765.7 | 730947.7 | 868406.8 | 648927.4 |
| >SCVPRZ2042F09 | | glutaminyl-trna synthetase | 27 | | 15 | | 161.3599 | | NE-0 | | E-21 | | 12400.99 | 15329.59 | 9947.018 | 13628.95 |
| >SCUTLR1058E05 | | spermidine synthase 1 | 19 | | 12 | | 127.7186 | | NE-0 | | E-0 | | 14944.81 | 23201.39 | 20277.16 | 20656.27 |
| **Table S1.** Continued |  | | |  | |  | |  | |  | |  |  |  |  |  |
| >SCRFSB1020B02 | | serine threonine-protein phosphatase 6... | 175 | | 95 | | 1248.6378 | | NE-0 | | E-21 | | 341905.6 | 379557.6 | 333791.4 | 338606.6 |
| >SCSFAD1123C03 | | 6-phosphofructokinase chloroplastic-like | 39 | | 13 | | 238.8922 | | E-0 | | E-21 | | 75016.63 | 20911.21 | 4763.05 | 18774.96 |
| >SCCCLR1C01E08 | | rna recognition motif -containing protein | 196 | | 117 | | 1247.7788 | | NE-0 | | E-21 | | 242328.6 | 275123.2 | 232412 | 248022.6 |
| >SCQSHR1022C01 | | beta- partial | 18 | | 10 | | 111.7126 | | NE-0 | | E-21 | | 3687.016 | 69496.47 | 176.5582 | 62758.76 |
| >SCJFRT1009B01 | | patatin t5 precursor | 17 | | 9 | | 132.0317 | | NE-0 | | E-0 | | 7983.405 | 136596.3 | 16331.03 | 123497.9 |
| >SCCCRZ1004G05 | | chaperonin 60 beta precursor | 199 | | 111 | | 1640.3547 | | NE-0 | | NE-21 | | 322240 | 323297.6 | 299951.1 | 292477.3 |
| >SCMCAM1101A10 | | karyopherin-beta 3 variant | 9 | | 8 | | 65.0093 | | E-0 | | E-21 | | 15636.45 | 8341.553 | 4196.399 | 7559.728 |
| >SCAGLR2033E02 | | clc2_orysj | 4 | | 1 | | 26.605 | | NE-0 | | E-21 | | 106.9704 | 5419.096 | 19.69416 | 4915.32 |
| >SCCCLR1C06E11 | | oxidative-stress responsive | 3 | | 2 | | 20.2947 | | NE-0 | | E-21 | | 3943.804 | 4151.246 | 3269.793 | 3765.798 |
| >SCBFSB1046C09 | | nuclear-pore anchor-like | 27 | | 17 | | 154.6113 | | E-21 | | E-0 | | 7647.63 | 23130.41 | 30347.07 | 21021.24 |
| >SCVPLR2005H09 | | 3-oxoacyl-synthase i | 28 | | 20 | | 219.4778 | | E-21 | | E-0 | | 16984.86 | 24437.36 | 26258.63 | 22212.33 |
| >SCJFRT1007A07 | | short chain alcohol | 6 | | 5 | | 49.7768 | | NE-0 | | E-21 | | 15377.02 | 42823.59 | 11274.35 | 38972.53 |
| >SCSBFL5014H12 | | lipoxygenase 2 | 8 | | 2 | | 46.8468 | | E-21 | | E-0 | | 531.2384 | 2376.585 | 2902.554 | 2168.075 |
| >SCBFRZ2016H11 | | ribophorin ii precursor | 25 | | 20 | | 175.5667 | | NE-0 | | E-0 | | 22407.37 | 57584.27 | 23840.1 | 52576.43 |
| >SCCCCL6004H07 | | beta-galactosidase expressed | 16 | | 6 | | 106.4066 | | E-0 | | E-21 | | 4709.118 | 2706.784 | 615.7299 | 2475.56 |
| >SCCCLR1048H09 | | catalase cata | 67 | | 43 | | 668.8414 | | E-21 | | NE-21 | | 178035.9 | 95822.58 | 325197.2 | 87673.27 |
| >SCCCRZ2C04F03 | | thioredoxin-dependent peroxidase | 12 | | 7 | | 116.8022 | | E-21 | | NE-21 | | 32589.29 | 15966.42 | 35880.11 | 14617.86 |
| >SCACRZ3036C09 | | act domain containing protein | 12 | | 7 | | 71.5636 | | E-21 | | E-0 | | 3620.28 | 13367.08 | 13706.72 | 12239.88 |
| >SCMCST1051A04 | | tripeptidyl peptidase ii | 23 | | 16 | | 135.7629 | | E-0 | | E-21 | | 28470.11 | 27600.57 | 22641.71 | 25366.68 |
| >SCVPRZ2043F04 | | atp synthase gamma chain | 180 | | 104 | | 1492.9312 | | E-21 | | NE-21 | | 710958.2 | 579632.2 | 874379 | 532859.5 |
| >SCCCLR1070F12 | | calnexin precursor | 45 | | 35 | | 358.3521 | | E-21 | | NE-21 | | 135344.4 | 139828.1 | 178953.2 | 128959.2 |
| >SCRLSD1012E03 | | probable ubiquitin ribosomal protein s27a fusion... | 226 | | 100 | | 1576.8175 | | E-21 | | NE-21 | | 234070.2 | 167346 | 246005.2 | 154865.4 |
| >SCCCFL5061H09 | | dihydrolipoyl dehydrogenase mitochondrial-like | 80 | | 55 | | 645.8615 | | NE-0 | | E-0 | | 89077.59 | 126891 | 105539.8 | 117759.4 |
| >SCSGLR1045D11 | | glutaryl- dehydrogenase | 8 | | 6 | | 45.0578 | | E-21 | | NE-21 | | 6520.363 | 5822.297 | 8689.453 | 5406.487 |
| >SCBFRT1069C12 | | wd-repeat domain phosphoinositide-interacting... | 4 | | 3 | | 17.709 | | E-0 | | NE-21 | | 4251.437 | 672.3406 | 2690.154 | 624.9944 |
| >SCCCST1002H09 | | pi starvation-induced protein | 6 | | 4 | | 46.1816 | | E-0 | | NE-21 | | 4110.506 | 3117.959 | 3892.471 | 2909.314 |
| >SCCCRT2001A05 | | heme oxygenasE-0 | 10 | | 5 | | 56.6951 | | NE-0 | | E-21 | | 6236.536 | 6243.141 | 4731.32 | 5863.327 |
| **Table S1.** Continued |  | | |  | |  | |  | |  | |  |  |  |  |  |
| >SCVPLB1015D09 | | vip1 protein | 21 | | 14 | | 159.6792 | | E-21 | | NE-21 | | 35136.33 | 24987.25 | 47390.15 | 23485.42 |
| >SCCCCL6004A10 | | succinyl- ligase beta-chain ligase beta-chain | 73 | | 52 | | 622.0395 | | E-21 | | NE-21 | | 200257.6 | 163278.7 | 209832 | 154238.3 |
| >SCCCCL3001A05 | | acoc_orysj | 83 | | 47 | | 768.8705 | | E-21 | | NE-21 | | 382800.1 | 194173.1 | 396829.5 | 184156.7 |
| >P00489 | | PYGM_RABIT Glycogen phosphorylase... | 40 | | 24 | | 250.9546 | | NE-0 | | E-21 | | 55047.29 | 80009.64 | 42201.15 | 76109.81 |
| >SCMCRT2103C07 | | acidic leucine-rich nuclear phosphoprotein 32... | 25 | | 13 | | 214.9496 | | E-0 | | NE-21 | | 30444.34 | 15093.86 | 21931.63 | 14378.68 |
| >SCEZLR1052F04 | | mitochondrial atp synthase precursor | 348 | | 188 | | 2614.9436 | | E-21 | | NE-21 | | 477559.5 | 475156.8 | 564870 | 452741.6 |
| >SCCCCL4007E05 | | diphosphate-fructose-6-phosphate... | 29 | | 6 | | 186.7013 | | E-21 | | NE-21 | | 5047.84 | 1229.181 | 5479.865 | 1178.199 |
| >SCBGFL5077B05 | | sap domain containing expressed | 36 | | 25 | | 212.062 | | NE-0 | | E-0 | | 30134.77 | 55889.39 | 30600.65 | 53681.94 |
| >SCCCRZ2004B10 | | 3-isopropylmalate dehydrogenase | 56 | | 38 | | 433.1876 | | E-21 | | E-0 | | 74361.05 | 94099.22 | 107285.5 | 90406.2 |
| >SCSGAM2105D01 | | ubiquitin-activating enzyme e1 expressed | 99 | | 55 | | 733.3053 | | NE-0 | | E-21 | | 163905 | 235930.1 | 162598.9 | 226733.9 |
| >SCEQLR1091D05 | | 60s ribosomal protein l5-1 | 413 | | 243 | | 3353.6115 | | NE-0 | | E-21 | | 616271.9 | 1305998 | 576340.7 | 1257170 |
| >SCQSLB1052H09 | | ubiquitin 11 | 224 | | 109 | | 1440.2458 | | E-0 | | NE-21 | | 226826.8 | 120700.9 | 205361 | 116594.1 |
| >SCQSRT2034F02 | | proline iminopeptidase-like | 25 | | 12 | | 154.2862 | | E-0 | | NE-21 | | 28462.36 | 14980.24 | 19199.36 | 14489.87 |
| >SCSBFL5015D06 | | snf1-related protein kinase regulatory subunit... | 16 | | 5 | | 87.3172 | | E-0 | | E-21 | | 4813.092 | 3883.842 | 2456.041 | 3765.678 |
| >SCCCCL3120F12 | | rh40_orysj | 36 | | 13 | | 219.1224 | | E-0 | | NE-21 | | 23847.74 | 10600.99 | 22310.53 | 10351.01 |
| >SCRFHR1007E11 | | cop9 signalosome complex subunit 6a | 56 | | 34 | | 371.1732 | | E-0 | | NE-21 | | 37362.75 | 20016.46 | 32685.37 | 19563.76 |
| >SCCCAM2C08A07 | | senescence-associated protein dh | 7 | | 5 | | 37.8831 | | E-0 | | E-21 | | 2890.548 | 2502.012 | 2203.827 | 2454.084 |
| >SCCCCL4008A08 | | loc100282868 precursor | 12 | | 8 | | 77.1027 | | E-0 | | NE-21 | | 12800.81 | 3146.509 | 5709.609 | 3092.195 |
| >SCBGST3105A03 | | glyoxylate reductase | 1 | | 1 | | 8.7718 | | NE-0 | | E-21 | | 404.5068 | 14346.22 | 328.7608 | 14160.24 |
| >SCJLRZ1027C05 | | dna-damage inducible protein | 14 | | 7 | | 110.6966 | | NE-0 | | E-21 | | 11443.65 | 18147.02 | 10588.12 | 18015.29 |
| >SCCCCL3002A09.b | | alanine aminotransferase | 80 | | 56 | | 678.0574 | | NE-0 | | E-0 | | 202204.6 | 505406 | 203762.3 | 502113.9 |
| >SCACCL6010C02 | | bifunctional aspartokinase homoserine... | 85 | | 47 | | 487.475 | | NE-0 | | E-21 | | 65739.17 | 86571.58 | 47541 | 86020.39 |
| >SCJLLR1105C04 | | nadp-specific isocitrate dehydrogenase | 57 | | 34 | | 502.0314 | | NE-0 | | E-0 | | 92510.06 | 112355.3 | 109416.8 | 111713.7 |
| >SCCCRZ2002F04 | | clh1_orysj | 60 | | 41 | | 465.982 | | NE-0 | | E-21 | | 100014.8 | 124453.4 | 76329.71 | 123804.9 |
| >SCJLRT1020A08 | | aglu_orysj | 42 | | 29 | | 295.2149 | | NE-0 | | E-0 | | 19244.59 | 150781.1 | 21605.46 | 150497.3 |
| >SCJFLR2035A04 | | rnase h domain-containing protein | 4 | | 4 | | 21.6426 | | E-0 | | NE-21 | | 1167.399 | 144.9018 | 435.074 | 144.6489 |
| >SCCCLB1025F10 | | secondary cell wall-related glycosyltransferase... | 7 | | 2 | | 37.07 | | E-0 | | NE-21 | | 2680.56 | 412.7753 | 1629.088 | 412.4008 |
| **Table S1.** Continued |  | | |  | |  | |  | |  | |  |  |  |  |  |
| >SCEQRT1025E03 | | cysteine proteinase inhibitor | 162 | | 102 | | 1195.8613 | | NE-21 | | E-0 | | 275449.6 | 440534.2 | 396208.1 | 440547.3 |
| >SCEPLR1051C09 | | multidomain cystatin | 16 | | 15 | | 147.907 | | E-21 | | NE-0 | | 61441.32 | 28034.2 | 63438.25 | 28044.22 |
| >SCSGFL1078C02 | | phytanoyl- dioxygenase domain-containing... | 9 | | 6 | | 62.8412 | | E-0 | | NE-0 | | 41729.73 | 9043.181 | 38538.06 | 9084.32 |
| >SCMCST1054G01 | | 3-mercaptopyruvate sulfurtransferase-like... | 12 | | 10 | | 67.7397 | | E-21 | | NE-0 | | 12314.11 | 10662.4 | 12490.04 | 10729.29 |
| >SCEQRZ3089H09 | | metal ion binding protein | 4 | | 2 | | 17.6808 | | NE-21 | | E-0 | | 1701.247 | 15213.95 | 1875.499 | 15425.23 |
| >SCUTSD1085B08 | | adenine nucleotide translocator | 52 | | 28 | | 409.9623 | | E-0 | | NE-0 | | 66774.71 | 25403.42 | 63463.33 | 25854.91 |
| >SCJFRT1007H07 | | af271894_1 lipoxygenase | 38 | | 17 | | 207.7176 | | NE-21 | | E-0 | | 10665.81 | 15031.55 | 11843.78 | 15299.31 |
| >SCSGRZ3061H04 | | cycloartenol-c-24-methyltransferase 1 | 10 | | 4 | | 59.9545 | | NE-21 | | E-21 | | 32710.04 | 33930.2 | 16234.64 | 34635.37 |
| >SCAGLR1021H11 | | nadh-ubiquinone oxidoreductase 13 kda-b subunit | 118 | | 71 | | 810.5534 | | E-21 | | NE-0 | | 175022.7 | 133216.1 | 201999.6 | 135991.9 |
| >SCQGLR1041H04 | | aquaporin pip2-1 | 21 | | 17 | | 170.9123 | | E-0 | | NE-0 | | 75480.15 | 18161.01 | 55717.89 | 18546.79 |
| >SCQSLB2055F11.b | | bri1-kd interacting protein 103 | 25 | | 19 | | 161.8403 | | E-21 | | NE-0 | | 10050.31 | 5301.024 | 12324.36 | 5430.382 |
| >SCRLFL4004D05 | | udp-glucuronic acid decarboxylase | 22 | | 13 | | 128.2554 | | NE-21 | | E-0 | | 18707.44 | 125930.8 | 22839.65 | 129314.4 |
| >SCRFLR1055E09 | | alpha-soluble nsf attachment protein | 39 | | 19 | | 270.6368 | | E-0 | | NE-0 | | 25086.5 | 15128.36 | 21985.48 | 15580.72 |
| >SCJFLR1073D03 | | gsa_orysj | 18 | | 8 | | 114.6194 | | E-21 | | NE-0 | | 8252.239 | 7086.912 | 8553.328 | 7314.693 |
| >SCUTLR1015G05 | | auxin-independent growth promoter | 5 | | 2 | | 26.843 | | E-0 | | E-21 | | 4183.449 | 1356.207 | 202.1771 | 1400.345 |
| >SCMCSD1059B05 | | argininosuccinate synthase | 67 | | 44 | | 469.7506 | | E-21 | | NE-0 | | 148344.2 | 70118.19 | 221706.7 | 72638.41 |
| >SCEPLR1030A02 | | stress-related protein | 21 | | 14 | | 121.325 | | E-0 | | NE-0 | | 61506.73 | 7803.571 | 23249.67 | 8108.076 |
| >SCJLLB2077A12 | | heat shock protein 90 | 621 | | 341 | | 5280.6091 | | E-0 | | NE-0 | | 1404223 | 1068935 | 1344565 | 1110969 |
| >SCQSRT2033A11 | | ras gtpase-activating protein-binding protein 1-like | 8 | | 4 | | 53.3578 | | E-0 | | E-21 | | 4432.478 | 3189.202 | 2604.93 | 3316.552 |
| >SCAGLB1070D03 | | protein disulfide isomerase | 575 | | 335 | | 4019.7566 | | E-21 | | E-0 | | 999001.7 | 1135185 | 1487718 | 1182717 |
| >SCEPFL3082H10 | | copper chaperone | 17 | | 12 | | 157.652 | | E-21 | | NE-0 | | 77342.9 | 54884.27 | 107173.7 | 57199.94 |
| >SCRUFL1118G01.b | | ago4b_orysj | 27 | | 13 | | 163.1605 | | E-21 | | NE-0 | | 25806.41 | 25803.79 | 28345.79 | 26929.96 |
| >SCCCRT2002G04 | | tktc_maize | 38 | | 28 | | 303.029 | | NE-21 | | E-21 | | 45643.09 | 100909.8 | 34290.13 | 105367.1 |
| >SCCCRT2001H08 | | ap-1 complex subunit gamma-1 | 8 | | 5 | | 47.958 | | NE-21 | | E-21 | | 3217.953 | 8204.493 | 658.4614 | 8605.073 |
| >SCEPLR1030H09 | | dynamin like protein 2a | 12 | | 8 | | 67.3017 | | NE-21 | | E-0 | | 5427.083 | 8373.557 | 7318.93 | 8808.275 |
| >SCCCLR1048A06 | | super-oxide dismutase | 20 | | 12 | | 206.7179 | | NE-21 | | E-0 | | 90410.08 | 151270.2 | 105994.7 | 159308.8 |
| >SCEPLR1051C05 | | cystathionine gamma-synthase | 12 | | 5 | | 83.6517 | | NE-21 | | E-21 | | 2814.152 | 4085.836 | 2192.489 | 4303.821 |
| **Table S1.** Continued |  | | |  | |  | |  | |  | |  |  |  |  |  |
| >SCCCLR1072B10 | | 26s proteasome non-atpase regulatory subunit 6 | 405 | | 266 | | 3093.8801 | | E-21 | | NE-0 | | 713145.1 | 540558.8 | 1154515 | 574143.5 |
| >SCCCHR1001E04 | | class iv family protein | 37 | | 24 | | 302.5096 | | NE-21 | | E-21 | | 153727 | 153717 | 88550.83 | 163478.4 |
| >SCEQHR1079G09 | | haloacid dehalogenase-like hydrolase... | 23 | | 16 | | 132.6663 | | NE-21 | | E-0 | | 26123.77 | 48774.03 | 34577.33 | 51891.08 |
| >SCEQLB2020F06 | | mitochondrial-processing peptidase beta subunit | 105 | | 70 | | 834.0594 | | NE-21 | | E-0 | | 170107.4 | 287188.9 | 299087.2 | 306005.5 |
| >SCJLFL1053H07 | | bsl2_orysj | 2 | | 2 | | 9.1788 | | NE-21 | | E-21 | | 1347.884 | 2990.377 | 597.0121 | 3189.799 |
| >SCEPAM1015E11 | | rubisco subunit binding-protein beta subunit | 13 | | 9 | | 75.852 | | E-0 | | NE-0 | | 31271.86 | 1463.795 | 6771.609 | 1562.146 |
| >SCAGLR1043G11 | | 40s ribosomal protein s3 | 394 | | 238 | | 3253.4612 | | NE-21 | | E-0 | | 734524.5 | 1140312 | 920593.8 | 1233218 |
| >SCCCRZ2C01E11 | | ill1_orysj | 11 | | 8 | | 91.9757 | | E-21 | | NE-0 | | 15609.7 | 4696.915 | 22657.71 | 5089.189 |
| >SCCCLR2C03H03 | | at5g19150 t24g5_50 | 26 | | 19 | | 165.4289 | | NE-21 | | E-21 | | 22707.2 | 23268.44 | 10471.95 | 25241.56 |
| >SCBGLR1096H12 | | propionyl- carboxylase beta chain | 17 | | 14 | | 118.9826 | | E-0 | | NE-0 | | 35097.72 | 11590.03 | 21252.65 | 12687.71 |
| >SCEQRT2027H04 | | dolichyl-diphosphooligosaccharide--protein... | 29 | | 16 | | 173.5003 | | E-21 | | NE-0 | | 10693.52 | 9345.925 | 14919.41 | 10233.88 |
| >SCACAM1070F09 | | ima1b_orysj | 25 | | 12 | | 196.6967 | | NE-21 | | E-21 | | 26262.97 | 30810.96 | 21914.93 | 34095.46 |
| >SCCCCL3004C10.b | | transaminase transferring nitrogenous groups | 29 | | 19 | | 202.3379 | | E-21 | | E-0 | | 9621.908 | 20176.19 | 28526.65 | 22420.29 |
| >SCVPRT2073B10 | | glyoxysomal fatty acid beta-oxidation... | 56 | | 33 | | 387.5833 | | E-21 | | NE-0 | | 68268.35 | 41793.45 | 103147.6 | 46585.84 |
| >SCSGRT2066A01 | | fumarylacetoacetate hydrolase | 21 | | 13 | | 158.0197 | | E-21 | | NE-0 | | 33086.21 | 19053.84 | 39659.45 | 21258.4 |
| >SCCCCL4014B10 | | presequence protease chloroplastic mitochondrial... | 28 | | 17 | | 174.8154 | | E-21 | | NE-0 | | 20054.32 | 10205.61 | 107555.1 | 11455.01 |
| >SCJFRT2059E04 | | puromycin-sensitive aminopeptidase isoform 1 | 25 | | 17 | | 170.3212 | | NE-21 | | E-21 | | 38388.13 | 35881.97 | 32707.55 | 40376.22 |
| >SCCCRZ1004H06 | | glutaredoxin subgroup i | 24 | | 18 | | 183.4941 | | E-0 | | NE-0 | | 51039.19 | 14299.07 | 41353.84 | 16127.11 |
| >SCQGST1033G06 | | cob22_orysj | 17 | | 9 | | 100.4087 | | NE-21 | | E-21 | | 3673.939 | 21220.27 | 1573.117 | 24160.69 |
| >SCRFAM1025D12 | | valyl trna synthetase | 19 | | 13 | | 129.4339 | | E-21 | | E-0 | | 8378.943 | 9528.301 | 11991.85 | 10854.33 |
| >SCUTFL1062H05 | | ubiquitin-like protein | 23 | | 7 | | 215.7332 | | E-21 | | NE-0 | | 12598.82 | 3215.468 | 31146.56 | 3677.824 |
| >SCCCRZ2C03E03 | | wound stress protein precursor | 7 | | 7 | | 43.3138 | | E-0 | | NE-0 | | 10780.18 | 2555.116 | 9962.961 | 2937.639 |
| >SCEZAM2059G02 | | thioredoxin-like 1 | 12 | | 8 | | 79.0355 | | E-0 | | NE-0 | | 12951.78 | 6472.717 | 10242.02 | 7473.954 |
| >SCCCCL4001H04 | | coatomer subunit delta | 58 | | 28 | | 377.3979 | | E-0 | | NE-0 | | 64114.69 | 24244.12 | 32083.11 | 28091.67 |
| >SCQSRT1035G12 | | universal stress protein family expressed | 8 | | 5 | | 51.7592 | | E-21 | | NE-0 | | 2424.146 | 1185.589 | 4750.498 | 1374.017 |
| >SCQSST3116B04 | | qltg3-1 | 6 | | 5 | | 48.0942 | | E-21 | | NE-0 | | 9187.646 | 7121.796 | 39501.46 | 8270.209 |
| >SCBGRT1047D12 | | hxk3_orysj | 12 | | 8 | | 70.3645 | | NE-21 | | E-21 | | 44664.15 | 46667.32 | 31618.61 | 54268.7 |
| **Table S1.** Continued |  | | |  | |  | |  | |  | |  |  |  |  |  |
| >SCEQRT1028A07 | | lactate dehydrogenase | 6 | | 4 | | 35.1814 | | NE-21 | | E-21 | | 2032.269 | 6471.751 | 1904.016 | 7536.504 |
| >SCCCCL3001F05 | | ru large subunit-binding protein subunit beta | 118 | | 68 | | 953.4877 | | NE-21 | | E-21 | | 188671.4 | 180628.2 | 176587.5 | 211816.9 |
| >SCJFRZ2033E06 | | stomatin-like protein 2 | 34 | | 25 | | 214.2932 | | E-0 | | NE-0 | | 47319.8 | 22415.5 | 40269.05 | 26298.93 |
| >SCMCCL6050B01 | | branched-chain-amino-acid aminotransferase | 23 | | 16 | | 154.3267 | | E-21 | | NE-0 | | 45488.53 | 10861.08 | 47427.96 | 12747.03 |
| >SCCCCL7001C09 | | suppressor enhancer of lin-12 protein 9 precursor | 26 | | 17 | | 192.1813 | | NE-21 | | E-0 | | 15255.58 | 27529.94 | 17062.81 | 32376.29 |
| >SCCCRZ1003A10 | | cell division cycle protein expressed | 153 | | 104 | | 1261.2591 | | NE-21 | | E-0 | | 332895.1 | 420749.2 | 337471 | 494924.8 |
| >SCVPRZ2042B04 | | protease candidate1 | 100 | | 68 | | 635.5616 | | E-0 | | NE-0 | | 147217.5 | 96500.64 | 108618.3 | 113881.1 |
| >SCVPRZ2043H12 | | methionine synthase protein | 44 | | 21 | | 332.3212 | | E-21 | | NE-0 | | 31496.72 | 10282.8 | 34402.68 | 12135.85 |
| >SCCCRZ1003E07 | | moco containing protein | 7 | | 2 | | 49.4682 | | E-0 | | NE-0 | | 1340.946 | 461.4353 | 995.2368 | 550.8515 |
| >SCVPLR1049C03 | | methylmalonate semi-aldehyde dehydrogenase | 39 | | 26 | | 286.8357 | | E-0 | | NE-0 | | 86217.27 | 43079.56 | 71486.13 | 51461.55 |
| >SCJFRT1007A12 | | ethylene receptor | 6 | | 3 | | 34.2764 | | E-21 | | NE-0 | | 1297.331 | 350.6846 | 2686.527 | 419.5365 |
| >SCBFRT1069A05 | | mrna capping c-terminal domain containing... | 24 | | 15 | | 149.8674 | | E-0 | | NE-0 | | 26037.84 | 18122.24 | 19213.34 | 21719.97 |
| >SCCCLR1024D02 | | atp-citrate expressed | 62 | | 47 | | 466.261 | | NE-21 | | E-21 | | 72774.39 | 77207 | 63597.36 | 92545.76 |
| >SCMCRZ3067G02 | | atypical receptor-like kinase mark precursor | 30 | | 17 | | 170.0077 | | E-0 | | NE-0 | | 27493.07 | 8792.677 | 19344.25 | 10554.63 |
| >SCMCLR1010F10 | | fip1 motif family expressed | 3 | | 1 | | 17.2944 | | NE-21 | | E-21 | | 553.0657 | 3785.652 | 351.7943 | 4546.825 |
| >SCMCST1050A01 | | nucleoporin nup43 | 11 | | 9 | | 68.013 | | E-21 | | NE-0 | | 16260.2 | 3460.165 | 17239.78 | 4163.922 |
| >SCCCST1002C11 | | loc100281207 precursor | 4 | | 4 | | 24.8516 | | NE-21 | | E-21 | | 3068.106 | 5953.97 | 2565.706 | 7172.015 |
| >SCEPCL6019A06 | | prolyl endopeptidase | 21 | | 12 | | 150.5659 | | NE-21 | | E-21 | | 28167.73 | 28859.55 | 15860.29 | 34820.55 |
| >SCJFRT2055F03.b | | succinate dehydrogenase flavoprotein precursor | 81 | | 55 | | 630.6849 | | NE-21 | | NE-0 | | 166367.5 | 151404.4 | 160946.9 | 183652.2 |
| >SCJLRT2049D05 | | heme-binding protein 2 | 14 | | 10 | | 145.3774 | | E-0 | | NE-0 | | 138552.9 | 10760.01 | 88564.24 | 13095.66 |
| >SCEPAM1051A02 | | predicted protein [Hordeum vulgare subsp. vulgare] | 11 | | 6 | | 57.3682 | | NE-21 | | E-0 | | 2537.247 | 7613.048 | 5394.041 | 9300.845 |
| >SCAGLR2011G10 | | ob-fold nucleic acid binding domain containing... | 16 | | 6 | | 147.7353 | | E-0 | | NE-0 | | 43497.08 | 19099.52 | 33001.49 | 23379.74 |
| >SCCCLR1001F10 | | fact complex subunit spt16 | 21 | | 10 | | 123.9478 | | E-0 | | NE-0 | | 12100.22 | 3265.551 | 7170.555 | 4004.201 |
| >SCCCRZ1002D03 | | pinin sdk mema protein conserved region... | 5 | | 3 | | 23.8042 | | E-0 | | E-21 | | 1970.916 | 1067.075 | 401.0014 | 1311.418 |
| >SCBFRZ2016E05 | | zinc finger transcription factor zfp30 | 40 | | 16 | | 238.4418 | | E-21 | | NE-0 | | 12706.34 | 11528.82 | 39464.63 | 14181.21 |
| >SCCCCL4004A04 | | ras-related protein rab-2-a | 105 | | 35 | | 700.8236 | | E-0 | | NE-0 | | 44214.92 | 29537.13 | 39707.29 | 36366.14 |
| >SCBGHR1058B11 | | pp2ac-2 - phosphatase 2a isoform 2 belonging... | 14 | | 4 | | 88.7217 | | E-0 | | E-21 | | 2415.739 | 1400.939 | 795.6635 | 1725.677 |
| **Table S1.** Continued |  | | |  | |  | |  | |  | |  |  |  |  |  |
| >SCJFST1011D09 | | dag protein | 14 | | 9 | | 86.0034 | | NE-21 | | E-21 | | 40059.28 | 52431.24 | 39722.88 | 65260.43 |
| >SCRFLR1012E06 | | scrk1_maize | 32 | | 23 | | 335.8111 | | NE-21 | | NE-0 | | 205384.2 | 200720.9 | 208936.4 | 249934.2 |
| >SCRURT2005F02 | | bowman-birk serine protease inhibitor precursor | 9 | | 8 | | 74.2667 | | NE-21 | | E-21 | | 12867.06 | 113296.5 | 11053.55 | 141584 |
| >SCEPAM2055H02 | | legumin-like protein | 13 | | 13 | | 94.475 | | E-21 | | NE-0 | | 42608 | 18363.68 | 59377.46 | 23071.9 |
| >SCCCLR2001B02 | | carbamoyl-phosphate synthase small chain | 21 | | 12 | | 146.7265 | | NE-21 | | E-0 | | 8297.006 | 11364.45 | 8601.214 | 14298.93 |
| >SCCCRZ1001B12 | | rhamnose biosynthetic enzyme expressed | 17 | | 7 | | 97.925 | | NE-21 | | E-0 | | 1579.386 | 3469.244 | 2805.366 | 4369.419 |
| >SCJLRZ1024A04 | | af465643_1 lipoxygenase | 79 | | 55 | | 547.9581 | | NE-21 | | E-21 | | 79446.95 | 121314.6 | 62498.72 | 153402 |
| >SCEQLR1029D10 | | acetyl-coenzyme a carboxylase | 11 | | 7 | | 52.8785 | | NE-21 | | E-21 | | 2890.338 | 8286.234 | 1604.966 | 10517.6 |
| >SCCCCL3001F10 | | ribosomal protein component of cytosolic... | 22 | | 4 | | 191.6009 | | NE-21 | | E-21 | | 480.7644 | 1539.105 | 318.8181 | 1955.506 |
| >SCEPAM1015F11 | | n-acetyl-gamma-glutamyl-phosphate reductase | 28 | | 19 | | 176.3599 | | E-21 | | NE-0 | | 34731.71 | 10999.42 | 39366.14 | 13986.46 |
| >SCCCLR2002G03 | | inorganic pyrophosphatase | 55 | | 24 | | 372.5985 | | NE-21 | | E-21 | | 25592.12 | 53542.32 | 24884.91 | 68423.48 |
| >SCCCCL3001C07.b | | alpha tubulin | 72 | | 36 | | 669.6514 | | E-21 | | NE-0 | | 128890.9 | 80791.83 | 130596.8 | 103345 |
| >SCEPSB1135E11 | | vacuolar atp synthase subunit g | 302 | | 149 | | 2115.1985 | | E-21 | | NE-0 | | 317051.7 | 315335.8 | 419811.4 | 404419.2 |
| >SCRUSB1063D08 | | aminoacylasE-0 precursor | 16 | | 13 | | 90.2429 | | E-0 | | E-21 | | 16088.28 | 10051.54 | 8655.785 | 12898.89 |
| >SCVPFL3047F02.b | | purple acid phosphatase precursor | 29 | | 20 | | 203.5377 | | E-0 | | NE-0 | | 49988.84 | 30447.57 | 47708.35 | 39203.73 |
| >SCRFLR1034D01 | | p68 rna helicase | 19 | | 7 | | 119.8216 | | E-0 | | NE-0 | | 13917.87 | 10146.25 | 13258.77 | 13104.54 |
| >SCCCLR1C01B06 | | dj-1 family protein | 8 | | 7 | | 61.6831 | | NE-21 | | E-0 | | 5287.953 | 10774.77 | 7079.429 | 14004.85 |
| >SCAGRT3047C03 | | peptidyl- cis-trans isomerase | 127 | | 75 | | 991.8356 | | NE-21 | | E-0 | | 404455.7 | 609752.3 | 559338 | 796457.8 |
| >SCSGHR1066E03 | | cold shock protein-1 | 10 | | 8 | | 104.5509 | | E-0 | | NE-0 | | 15271.17 | 5570.054 | 7749.754 | 7300.073 |
| >SCCCHR1002H03 | | 6-phosphogluconate decarboxylating | 50 | | 22 | | 412.7844 | | E-21 | | E-0 | | 42308.74 | 51196.06 | 80027.77 | 67374.03 |
| >SCRLLR1038E03 | | ---NA--- | 389 | | 179 | | 2459.316 | | E-21 | | NE-0 | | 343523.5 | 168499.4 | 374987.1 | 221767.9 |
| >SCEZLR1031A11 | | acetyl- cytosolic 1 | 34 | | 22 | | 212.4588 | | E-21 | | E-0 | | 21700.13 | 23520.74 | 61964.22 | 31014 |
| >SCJFRZ1006H10 | | threonine synthase | 21 | | 12 | | 144.9893 | | NE-21 | | NE-0 | | 16518.85 | 13470.36 | 14217.22 | 17766.35 |
| >SCSGLR1045G02 | | rh9_orysj | 10 | | 5 | | 54.8735 | | E-0 | | E-21 | | 7853.337 | 4078.961 | 2357.435 | 5382.759 |
| >SCMCRT2107G02 | | aspartate-semialdehyde dehydrogenase | 12 | | 8 | | 103.813 | | E-21 | | NE-0 | | 25829.11 | 20631.46 | 30040.75 | 27257.55 |
| >SCUTSD1024F06 | | 3-ketoacyl- thiolase peroxisomal precursor | 26 | | 18 | | 238.7143 | | E-21 | | NE-0 | | 108690.2 | 37748.4 | 113640.9 | 50057.1 |
| >SCCCCL5002H02 | | globulin-1 s allele precursor | 20 | | 18 | | 265.8498 | | E-21 | | NE-0 | | 82181.6 | 21216.6 | 457292.2 | 28227.8 |
| **Table S1.** Continued |  | | |  | |  | |  | |  | |  |  |  |  |  |
| >SCQSRT1034D04 | | transmembrane 9 superfamily protein member 4 | 18 | | 8 | | 112.6511 | | NE-21 | | E-0 | | 380.4992 | 14427.35 | 921.0051 | 19546.78 |
| >SCJFRZ2027G04 | | phenylalanyl-trna synthetase beta chain | 35 | | 17 | | 201.2606 | | NE-21 | | E-0 | | 34885.21 | 54565.86 | 52370.81 | 73952.76 |
| >SCJFRT1009E03 | | maize insect resistance1 precursor | 8 | | 3 | | 66.0039 | | NE-21 | | E-0 | | 384.4646 | 15976.62 | 745.5031 | 21687.91 |
| >SCCCLR1C01H06 | | topoisomerase-like protein | 19 | | 12 | | 116.4818 | | E-21 | | NE-0 | | 22874.32 | 5583.625 | 46788.92 | 7584.866 |
| >SCRLFL4025A02 | | af458962_1 cdc5 protein | 17 | | 7 | | 89.5041 | | E-0 | | NE-0 | | 5145.047 | 1434.214 | 2650.658 | 1953.87 |
| >SCQGLR1062D04 | | udp-glucose pyrophosphorylase | 115 | | 67 | | 1016.7641 | | NE-21 | | E-21 | | 377805.3 | 491240.9 | 360357.4 | 669449 |
| >SCCCCL4011B08 | | 2-isopropylmalate synthase b | 80 | | 57 | | 659.0114 | | E-0 | | NE-0 | | 212387.4 | 122253.9 | 189894.7 | 166916.3 |
| >SCQSRT1034D03 | | loc100282597 precursor | 13 | | 6 | | 80.1427 | | NE-21 | | E-21 | | 8765.896 | 12396.74 | 4276.14 | 16949.74 |
| >SCCCCL3080H09.b | | glutamate dehydrogenase | 49 | | 27 | | 310.2596 | | NE-21 | | E-0 | | 34070.8 | 46302.3 | 42390.75 | 63347.22 |
| >SCSGAM2105G02 | | mar binding protein | 5 | | 2 | | 29.3544 | | E-0 | | NE-0 | | 4918.739 | 230.5194 | 1825.902 | 316.1165 |
| >SCCCRT2004D04 | | alcohol dehydrogenase 1 | 89 | | 70 | | 861.8701 | | NE-21 | | E-0 | | 315919.3 | 715396.4 | 369319.1 | 984478.7 |
| >SCCCRT2001B05 | | aminolevulinic acid dehydratase | 26 | | 17 | | 171.1902 | | E-21 | | NE-0 | | 14924.84 | 8168.829 | 22587.5 | 11284.3 |
| >SCJLFL4098H05 | | tip120 protein | 40 | | 30 | | 248.1536 | | NE-21 | | E-21 | | 28672.93 | 27495.89 | 24687.29 | 37989.71 |
| >SCUTLR2023D11 | | aldehyde dehydrogenase family 7 member a1 | 38 | | 26 | | 255.0943 | | E-0 | | NE-0 | | 60259.48 | 35711.93 | 53284.55 | 49467.38 |
| >SCEQLR1050G04 | | dihydrodipicolinate synthase 2 | 14 | | 6 | | 80.496 | | E-0 | | NE-0 | | 7433.244 | 3244.413 | 5212.532 | 4545.602 |
| >SCCCRZ1003A11 | | glutamine synthetase | 51 | | 32 | | 457.7516 | | NE-21 | | E-0 | | 94232.76 | 274729.8 | 146333.6 | 385881.8 |
| >SCBGLR1023B04 | | udp-sulfoquinovose synthase | 16 | | 7 | | 92.6635 | | E-0 | | NE-0 | | 16530.31 | 8614.156 | 13320.13 | 12104.48 |
| >SCCCCL3001H07 | | f-box ankyrin repeat protein skip35-like | 21 | | 9 | | 122.3541 | | NE-21 | | E-0 | | 12609.29 | 18044.71 | 15741.29 | 25522.43 |
| >SCQSLR1018B02 | | chromosome region maintenance protein | 15 | | 7 | | 102.5908 | | NE-21 | | E-0 | | 3056.833 | 6784.304 | 3963.241 | 9616.037 |
| >SCCCRZ2003A07 | | aminopeptidase m | 87 | | 48 | | 598.4648 | | NE-21 | | E-21 | | 110617.9 | 97053.93 | 92684.49 | 137589.4 |
| >SCSBSD2058B02 | | aldose 1-epimerase | 19 | | 12 | | 121.8085 | | NE-21 | | E-0 | | 4892.929 | 26869.36 | 7885.853 | 38130.83 |
| >SCRFRT3059F04 | | uridine monophosphate synthase | 19 | | 13 | | 118.7684 | | E-21 | | NE-0 | | 28360.23 | 12215.18 | 30418.28 | 17342.09 |
| >SCJLRZ1019A04 | | calcium-dependent protein kinase sk5-like | 45 | | 25 | | 280.2227 | | E-0 | | NE-0 | | 29661.68 | 7439.057 | 12142.09 | 10590.72 |
| >SCCCRZ1001C05 | | phosphoglycerate kinase | 97 | | 55 | | 849.4121 | | NE-21 | | E-0 | | 309307.2 | 379834.6 | 355789.9 | 541411 |
| >SCBGLR1027E10 | | 3-hydroxyisobutyryl- hydrolase-like protein... | 7 | | 5 | | 44.4597 | | E-0 | | NE-0 | | 6697.5 | 1751.861 | 2999.477 | 2503.341 |
| >SCCCST1002D07 | | glyoxalase i | 26 | | 20 | | 204.2009 | | NE-21 | | E-0 | | 101601.4 | 194117.3 | 121108.3 | 277624.3 |
| >SCJLLR1033A07 | | hydroxyacylglutathione hydrolase | 30 | | 24 | | 204.0219 | | E-0 | | NE-0 | | 95237.97 | 65068.47 | 93782.52 | 93321.25 |
| **Table S1.** Continued |  | | |  | |  | |  | |  | |  |  |  |  |  |
| >SCJFRZ2010G03 | | r40c1 protein - rice | 31 | | 15 | | 372.5379 | | NE-21 | | E-0 | | 224111.3 | 302982.3 | 276490.4 | 436147.7 |
| >SCCCLR1C01C08 | | chloroplast outer envelope 86-like protein | 50 | | 29 | | 306.1569 | | E-0 | | NE-0 | | 45060.27 | 16676.13 | 25635.49 | 24010.59 |
| >SCVPRT2075E07 | | c-1-tetrahydrofolate cytoplasmic | 11 | | 8 | | 69.6575 | | E-0 | | NE-0 | | 26721.03 | 3533.091 | 20813.83 | 5097.71 |
| >SCCCCL3002F02.b | | betaine aldehyde dehydrogenase | 34 | | 21 | | 282.924 | | E-21 | | NE-0 | | 57725.95 | 43964.56 | 68680.09 | 63525.59 |
| >SCEQRT1029D09 | | glucose-6-phosphate isomerase | 133 | | 79 | | 905.1306 | | NE-21 | | E-0 | | 105731 | 138714.9 | 109463 | 200525.1 |
| >SCRLFL1004D11 | | dihydroxy-acid dehydratase | 36 | | 27 | | 349.203 | | E-0 | | NE-0 | | 104953.3 | 63244.99 | 101291.8 | 92142.34 |
| >SCQGLR1019B02 | | ferredoxin-sulfite reductase precursor | 28 | | 20 | | 197.9992 | | E-0 | | E-21 | | 61646.55 | 33767.91 | 32806.94 | 49253.7 |
| >SCEPAM2053F09 | | cyclase dehydrase family protein | 17 | | 12 | | 133.167 | | E-21 | | NE-0 | | 17463.18 | 6281.617 | 21660.67 | 9210.173 |
| >SCJFRZ2031F10 | | acetyltransferase 1-like | 8 | | 6 | | 51.5425 | | NE-21 | | E-0 | | 10369.8 | 13683.24 | 10371.67 | 20121.15 |
| >SCEPCL6021H04 | | cytochrome b5 | 120 | | 73 | | 991.5899 | | NE-21 | | NE-0 | | 236304.3 | 183604.4 | 243752.6 | 270263.5 |
| >SCCCLR1C05E01 | | signal recognition particle 9 kda protein | 20 | | 12 | | 121.976 | | NE-21 | | E-0 | | 8539.665 | 25458.94 | 10901.45 | 37540.98 |
| >SCCCLR1076E03 | | brassinosteroid biosynthesis-like protein | 36 | | 18 | | 215.9217 | | NE-21 | | E-21 | | 20966.05 | 16219.72 | 10917.65 | 24003.16 |
| >SCVPLR1028C10 | | ornithine carbamoyltransferase | 25 | | 20 | | 177.9965 | | NE-21 | | E-21 | | 17607.61 | 21077.51 | 14434.94 | 31295.4 |
| >SCJLLR1054H06 | | pur alpha-1 | 14 | | 9 | | 80.6289 | | E-0 | | NE-0 | | 18506.65 | 7837.401 | 12761.71 | 11744.34 |
| >SCCCCL3001C03 | | citrate synthase mitochondrial expressed | 61 | | 46 | | 461.2317 | | NE-21 | | NE-0 | | 159397.4 | 126388 | 141184.1 | 189466.9 |
| >SCCCCL4003G01 | | serine-threonine kinase receptor-associated protein | 19 | | 12 | | 100.2133 | | E-0 | | NE-0 | | 12959.93 | 5153.485 | 11203.1 | 7725.854 |
| >SCJFRZ1007H03 | | aspartate kinase-homoserine dehydrogenase | 121 | | 84 | | 956.9267 | | NE-21 | | E-0 | | 215527.3 | 235045.6 | 305899.5 | 352380.5 |
| >SCBFLR1083D11 | | seryl-trna synthetase | 34 | | 19 | | 201.9085 | | NE-21 | | NE-0 | | 17802.41 | 14209.1 | 17716.95 | 21429.02 |
| >SCCCRT1002A01 | | importin alpha-2 subunit | 19 | | 11 | | 113.4126 | | NE-21 | | E-21 | | 11646.02 | 20079.64 | 8296.315 | 30368.05 |
| >SCJFRT2057F01 | | subtilisin-like proteinase | 17 | | 11 | | 97.3431 | | NE-21 | | E-21 | | 7817.369 | 22807.2 | 7589.001 | 34792.62 |
| >SCRFRT3058D06 | | rh52b_orysj | 40 | | 16 | | 271.0959 | | E-0 | | NE-0 | | 16649.44 | 3018.379 | 7659.141 | 4622.378 |
| >SCMCLR1123A03 | | vhs and gat domain protein | 13 | | 5 | | 67.8877 | | NE-21 | | E-0 | | 1776.447 | 2640.003 | 2114.112 | 4050.854 |
| >SCJFST1015G05 | | dnaj protein | 17 | | 7 | | 98.3281 | | E-0 | | NE-0 | | 5239.415 | 1964.189 | 4459.301 | 3016.247 |
| >SCJFRZ2025F03 | | farnesylcysteine lyase-like | 5 | | 4 | | 28.287 | | E-0 | | NE-0 | | 4885.708 | 2515.415 | 3799.67 | 3869.415 |
| >SCAGHR1016D09 | | acetolactate small | 20 | | 15 | | 154.4059 | | NE-21 | | E-21 | | 19396.47 | 29239.07 | 8721.339 | 45499.67 |
| >SCRFLR2034B06 | | at-hook protein 1 | 16 | | 7 | | 95.2811 | | NE-21 | | NE-0 | | 7342.632 | 4754.321 | 5388.878 | 7467.916 |
| >SCSGRT2066B08 | | rh52c_orysj | 15 | | 8 | | 106.8476 | | NE-21 | | E-0 | | 1309.541 | 14114.26 | 2112.538 | 22183.35 |
| **Table S1.** Continued |  | | |  | |  | |  | |  | |  |  |  |  |  |
| >SCJFRZ2032H05 | | tubulin-specific chaperone a | 9 | | 8 | | 64.93 | | E-21 | | NE-0 | | 12034.44 | 6456.251 | 18514.77 | 10166.81 |
| >SCCCLR2C02F10 | | achain complex of hsp90 n-terminal and sgt1 cs... | 30 | | 5 | | 346.3485 | | NE-21 | | E-21 | | 21972.75 | 48010.27 | 17560.82 | 75629.67 |
| >SCEZLR1031E10 | | translationally-controlled tumor protein | 12 | | 10 | | 125.9446 | | NE-21 | | E-21 | | 44786.28 | 44482.63 | 31590.9 | 70360.5 |
| >SCAGRT2039A02 | | asparagine synthetase | 24 | | 16 | | 142.802 | | E-21 | | NE-0 | | 12988.34 | 6238.179 | 24271.43 | 9899.098 |
| >SCCCFL6001G05 | | auxin response factor 7a | 20 | | 9 | | 108.7202 | | E-21 | | NE-0 | | 4536.967 | 3130.943 | 5188.852 | 4981.613 |
| >SCJFST1015F01 | | glycoside family 28 precursor | 9 | | 4 | | 50.6523 | | NE-21 | | E-0 | | 565.7834 | 1951.493 | 2085.221 | 3119.36 |
| >SCSGAM2076D04 | | actin [Brassica napus var. napus] | 42 | | 7 | | 399.5254 | | NE-21 | | E-0 | | 8028.612 | 10639.38 | 13549.69 | 17096.08 |
| >SCEPFL4175E04 | | gtp-binding protein | 111 | | 54 | | 855.4305 | | NE-21 | | NE-0 | | 109429.6 | 108118.5 | 156686.3 | 173767.6 |
| >SCSFFL3090D03 | | h2b1_wheat | 11 | | 2 | | 108.8972 | | NE-21 | | E-0 | | 1488.735 | 5637.372 | 2421.792 | 9085.678 |
| >SCJFRZ3C03A07.b | | d-amino acid oxidase | 6 | | 3 | | 33.1243 | | NE-21 | | E-21 | | 6096.713 | 5715.711 | 2498.878 | 9245.482 |
| >SCCCLB1004G11 | | transcription factor apfi | 152 | | 92 | | 989.8626 | | E-0 | | NE-0 | | 181263.2 | 78276.84 | 174570.2 | 126622.9 |
| >SCCCLR2003A05 | | cullin- expressed | 8 | | 4 | | 40.1554 | | NE-21 | | E-21 | | 3191.496 | 5004.104 | 1576.257 | 8096.117 |
| >SCEQRT2094H09 | | receptor-mediated endocytosis 1 isoform i | 8 | | 3 | | 45.8398 | | NE-21 | | E-0 | | 453.6316 | 749.1057 | 667.2405 | 1213.935 |
| >SCSFRT2068B02 | | receptor-like kinase | 17 | | 7 | | 104.9318 | | E-21 | | NE-0 | | 4131.051 | 1774.282 | 5107.771 | 2897.995 |
| >SCJFLR1074A06 | | pdi-like protein | 40 | | 30 | | 330.8388 | | E-0 | | NE-0 | | 183540.1 | 107895.2 | 157599.2 | 177371.3 |
| >SCCCCL3120E12 | | at1g05520 t25n20_16 | 13 | | 8 | | 70.7751 | | NE-21 | | E-21 | | 2705.186 | 11726.74 | 1518.933 | 19324.37 |
| >SCEZRZ1013F01 | | cullin-1-like isoform 1 | 33 | | 18 | | 198.0795 | | NE-21 | | E-0 | | 7829.818 | 25037.35 | 15623.14 | 41650.58 |
| >SCJFRZ2005G03 | | swib mdm2 domain containing protein | 19 | | 14 | | 147.6731 | | E-21 | | NE-0 | | 22951.72 | 4222.274 | 26844.58 | 7029.609 |
| >SCVPLR1049A01 | | smc3 protein | 42 | | 22 | | 225.6356 | | NE-21 | | E-21 | | 12048.24 | 11200.47 | 10280.78 | 18665.82 |
| >SCCCLR1070A12 | | dihydrolipoyllysine-residue succinyltransferase... | 37 | | 17 | | 257.8554 | | E-21 | | NE-0 | | 96013.11 | 34774.6 | 100769.1 | 58054.22 |
| >SCCCCL4011H03 | | ribulose-phosphate 3-epimerase | 6 | | 5 | | 44.5961 | | NE-21 | | E-21 | | 9744.957 | 13348.71 | 9176.036 | 22435.05 |
| >SCJLLR1011F03 | | ketol-acid chloroplastic-like | 54 | | 36 | | 440.086 | | NE-21 | | E-0 | | 171108.5 | 223383.2 | 188328.1 | 375617.3 |
| >SCCCRZ2002H11 | | pentatricopeptide repeat-containing protein... | 50 | | 16 | | 296.6477 | | NE-21 | | E-0 | | 15523.22 | 15804.1 | 20478.14 | 26739.4 |
| >SCCCHR1004H09 | | choline-phosphate cytidylyltransferase b | 5 | | 3 | | 29.7236 | | E-21 | | NE-0 | | 1667.216 | 1123.523 | 2645.953 | 1904.696 |
| >SCCCAM1001G03 | | beta-ketoacyl-acp synthase | 9 | | 4 | | 48.2706 | | NE-21 | | NE-0 | | 4649.644 | 3224.028 | 4142.611 | 5506.579 |
| >SCRLLR1109C03 | | arginine serine-rich splicing factor | 14 | | 9 | | 79.1207 | | E-21 | | NE-0 | | 11569.13 | 2052.711 | 12240.41 | 3509.128 |
| >SCQGLR1086B07 | | selenium binding protein | 19 | | 9 | | 127.7782 | | E-21 | | NE-0 | | 7698.515 | 3268.772 | 44171.77 | 5606.784 |
| **Table S1.** Continued |  | | |  | |  | |  | |  | |  |  |  |  |  |
| >SCUTST3090E03 | | unknow protein | 11 | | 6 | | 55.1742 | | E-21 | | NE-0 | | 3098.405 | 2604.365 | 4829.062 | 4506.125 |
| >SCQSRT1036D03 | | pathogenesis-related protein 1 | 19 | | 10 | | 157.4153 | | NE-21 | | E-21 | | 8623.274 | 40430.5 | 6541.682 | 70516.4 |
| >SCCCCL3002C07.b | | amy3c_orysj | 22 | | 16 | | 221.7233 | | E-0 | | NE-0 | | 118016.9 | 7184.268 | 66777.42 | 12595.85 |
| >SCVPLR2027B06 | | ubiquitin-associated ts-n domain-containing | 12 | | 4 | | 81.2512 | | NE-21 | | E-21 | | 15910.05 | 12810.09 | 1541.389 | 22560.89 |
| >SCQSRT2036A12 | | monodehydroascorbate reductase | 53 | | 32 | | 423.8478 | | NE-21 | | E-0 | | 87285.34 | 94612.6 | 112541 | 167025.4 |
| >SCCCRZ1001E09 | | uncharacterized protein LOC100191561 [Zea mays] | 329 | | 139 | | 2248.5165 | | E-0 | | NE-0 | | 228858.8 | 110777.8 | 225016.6 | 195636.9 |
| >SCCCLR1C04E01 | | wheat adenosylhomocysteinase-like protein | 39 | | 29 | | 441.5666 | | NE-21 | | NE-0 | | 217368.5 | 180554.2 | 283080.5 | 321103.5 |
| >SCBFSB1049C11 | | cgep_orysj | 8 | | 4 | | 39.862 | | NE-21 | | E-0 | | 2957.885 | 4772.647 | 4667.987 | 8504.882 |
| >SCRLLR1059B07 | | like protein | 15 | | 6 | | 110.2418 | | E-0 | | NE-0 | | 6800.722 | 2304.057 | 6486.117 | 4120.669 |
| >SCCCLR1001B11 | | pyridoxin biosynthesis protein er1 | 12 | | 9 | | 96.5465 | | NE-21 | | E-0 | | 19475.31 | 37711.94 | 34624.76 | 67730.24 |
| >SCCCCL3001B09.b | | aconitate hydratase 1 | 106 | | 67 | | 793.2703 | | NE-21 | | E-0 | | 125324.8 | 147981.7 | 145864.5 | 267525.8 |
| >SCCCLR1022D04 | | mdhg_orysj | 10 | | 5 | | 76.2918 | | E-0 | | NE-0 | | 8740.465 | 2769.662 | 5426.668 | 5007.454 |
| >SCJLRT1014A07 | | ac090882_14 dehydratase deaminase | 5 | | 2 | | 23.1247 | | NE-21 | | NE-0 | | 1062.78 | 745.2839 | 960.77 | 1348.763 |
| >SCRLRT3033C05 | | gibberellin 20 oxidase 2 | 28 | | 21 | | 171.3414 | | NE-21 | | E-21 | | 4072.503 | 26679.31 | 3579.308 | 48492.59 |
| >SCJLLR1107G02 | | late-embryogenesis-abundant protein | 8 | | 6 | | 60.6813 | | E-0 | | NE-0 | | 33245.43 | 6074.199 | 19980.44 | 11049.53 |
| >SCCCCL1001A02 | | translocase of chloroplast 34 | 15 | | 11 | | 90.3312 | | E-0 | | NE-0 | | 11396.76 | 5444.552 | 10983.96 | 9912.481 |
| >SCCCCL7037A10 | | loc100282411 precursor | 27 | | 20 | | 247.3887 | | NE-21 | | E-21 | | 69744.79 | 105846 | 58123.57 | 193589.7 |
| >SCUTFL3075D02 | | thioredoxin domain-containing protein 9 | 88 | | 51 | | 665.9546 | | NE-21 | | E-0 | | 177307 | 190994.5 | 214851.4 | 351326.9 |
| >SCRFLR2034A07 | | ma3 domain-containing protein | 33 | | 22 | | 272.9223 | | E-0 | | NE-0 | | 76354.49 | 28008.09 | 50187.56 | 51823.68 |
| >SCQSRT2034G08 | | alpha-galactosidase expressed | 26 | | 15 | | 173.2528 | | NE-21 | | E-21 | | 5217.268 | 65524.54 | 3819.028 | 121285.9 |
| >SCEZAM2059G08 | | pwwp domain containing protein | 4 | | 3 | | 24.4405 | | E-0 | | NE-0 | | 7140.614 | 268.4423 | 811.5248 | 502.5144 |
| >SCMCRZ3068H09 | | s-adenosylmethionine synthetase 1 | 19 | | 7 | | 151.1013 | | NE-21 | | E-0 | | 1848.258 | 12281.99 | 2505.215 | 23113.9 |
| >SCEQLR1091F06 | | sorbitol dehydrogenase | 30 | | 21 | | 284.4472 | | E-21 | | NE-0 | | 116466 | 31413.24 | 167142.1 | 59651.2 |
| >SCCCLR1C05F08 | | fumarate hydratase chloroplastic-like | 19 | | 14 | | 129.2365 | | E-0 | | NE-0 | | 21137.66 | 7143.467 | 18879.05 | 13640.97 |
| >SCJLRT1014F08 | | progesterone 5-beta- | 9 | | 5 | | 62.509 | | NE-21 | | E-0 | | 5806.666 | 6867.778 | 11005.56 | 13304.04 |
| >SCACRZ3109E01 | | xaa-pro aminopeptidase 1 | 25 | | 14 | | 163.0807 | | NE-21 | | E-0 | | 9572.21 | 10963.84 | 11514.54 | 21308.39 |
| >SCUTLR2023H05 | | r40g2 protein | 23 | | 12 | | 221.494 | | NE-21 | | E-0 | | 7703.439 | 17602.11 | 31186.44 | 34541.32 |
| **Table S1.** Continued |  | | |  | |  | |  | |  | |  |  |  |  |  |
| >SCACLR2007H10 | | va0d_orysj | 8 | | 5 | | 51.4652 | | NE-21 | | E-21 | | 2989.956 | 15511.95 | 428.9026 | 31064 |
| >SCCCCL4013H06 | | coproporphyrinogen iii oxidase | 19 | | 13 | | 135.6832 | | NE-21 | | E-21 | | 24151.03 | 27019.77 | 20355.45 | 54416.96 |
| >SCSBRZ3117C09 | | clpc1_orysj | 55 | | 15 | | 352.4594 | | E-21 | | NE-0 | | 20172.2 | 7061.096 | 41075.3 | 14877.87 |
| >SCRULB1060C09 | | cathepsin b-like | 17 | | 10 | | 133.737 | | NE-21 | | E-21 | | 25132.57 | 46530.27 | 21095.82 | 99015.48 |
| >SCCCLR1C07C11 | | fiber protein fb15 | 4 | | 4 | | 40.9095 | | NE-21 | | E-21 | | 2800.244 | 2967.319 | 2411.688 | 6325.932 |
| >SCCCLR1065E09 | | 3-dehydroquinate synthase | 19 | | 14 | | 127.9345 | | NE-21 | | E-21 | | 8311.268 | 18401.9 | 5941.92 | 39236.58 |
| >SCQGLR1019B10 | | ac077693_3 eukaryotic initiation factor subunit | 19 | | 10 | | 142.0314 | | E-21 | | NE-0 | | 15553.62 | 2005.269 | 19065.23 | 4283.104 |
| >SCEQRT2095D10 | | gdp-mannose -epimerase 1 | 40 | | 17 | | 298.3393 | | E-21 | | NE-0 | | 15608.31 | 12066.73 | 32949.84 | 25947.64 |
| >SCCCCL4007F09 | | adenylosuccinate lyase | 30 | | 13 | | 218.9687 | | NE-21 | | E-21 | | 13769.58 | 27693.02 | 10373.83 | 59589.14 |
| >SCCCLR1065D04 | | beta-cyanoalanine synthase | 5 | | 5 | | 33.438 | | NE-21 | | E-0 | | 238.4804 | 1076.118 | 2069.228 | 2324.78 |
| >SCSGFL1083E06 | | imidazole glycerol phosphate synthase chloroplast... | 11 | | 7 | | 66.4856 | | E-21 | | NE-0 | | 7257.865 | 2535.442 | 13968.84 | 5483.522 |
| >SCEZLB1010A09 | | anamorsin homolog | 8 | | 6 | | 43.5762 | | E-0 | | NE-0 | | 7638.322 | 623.2277 | 5106.552 | 1356.134 |
| >SCMCAM2084F10.b | | cigr1_orysj | 5 | | 4 | | 24.9975 | | NE-21 | | E-0 | | 808.4208 | 2573.248 | 2240.177 | 5613.158 |
| >SCCCCL4014E03 | | set domain-containing protein set104 | 30 | | 14 | | 178.5288 | | E-0 | | NE-0 | | 42396.89 | 2853.61 | 12535.67 | 6252.984 |
| >SCMCLB2081E11 | | dna-dependent atpase snf2h | 9 | | 5 | | 47.0861 | | NE-21 | | E-21 | | 1990.877 | 3774.249 | 938.3026 | 8276.813 |
| >SCEZLR1031A02 | | fructose tagatose bisphosphate aldolase | 7 | | 4 | | 43.4762 | | NE-21 | | E-0 | | 418.9709 | 4383.906 | 708.6348 | 9623.53 |
| >SCBFRZ2045G10 | | metallopeptidase family m24 containing expressed | 16 | | 6 | | 83.8374 | | E-0 | | NE-0 | | 2438.725 | 981.3237 | 1103.247 | 2157.212 |
| >SCCCCL3004C07.b | | regulatory protein viviparous-1 protein viviparous-1 | 8 | | 5 | | 47.7295 | | NE-21 | | E-21 | | 165.2351 | 3122.073 | 47.47746 | 6865.82 |
| >SCSGFL5C03H04 | | chromatin complex subunit a101 | 12 | | 7 | | 71.9779 | | NE-21 | | NE-0 | | 7285.664 | 6351.098 | 6355.65 | 14070.68 |
| >SCSGFL4C03C08 | | disease resistance response protein 206 | 12 | | 8 | | 73.8497 | | NE-21 | | E-21 | | 25347.95 | 34295.6 | 17178.97 | 76056.39 |
| >SCJFRT1058F10 | | ferredoxin--nadp root isozyme | 22 | | 13 | | 154.9769 | | E-21 | | NE-0 | | 24374.23 | 8857.359 | 53189.86 | 19739.95 |
| >SCCCLB1026C11 | | transposon pong sub-class | 52 | | 26 | | 357.4911 | | NE-21 | | E-21 | | 44137.82 | 28791.7 | 21797.05 | 64203.66 |
| >SCCCRZ1001F01 | | chaperone dna j2 | 43 | | 15 | | 280.9767 | | E-0 | | NE-0 | | 29354.22 | 11709.75 | 16493.7 | 26273.05 |
| >SCEPSB1130D06 | | retrotransposon line subclass | 20 | | 12 | | 136.3313 | | E-21 | | NE-0 | | 9178.465 | 2803.421 | 12814.13 | 6381.456 |
| >SCBGSD2049D08 | | cdpk-related protein kinase | 3 | | 1 | | 12.1363 | | NE-21 | | E-21 | | 439.6682 | 1438.646 | 344.0187 | 3310.968 |
| >SCBFSB1048E08 | | ent domain containing protein | 9 | | 5 | | 57.0652 | | E-0 | | NE-0 | | 3670.505 | 190.4852 | 2541.492 | 441.2616 |
| >SCCCRZ2C01A04 | | cinnamoyl- reductase | 12 | | 10 | | 71.6016 | | E-21 | | NE-0 | | 8586.397 | 2441.43 | 10656.51 | 5661.807 |
| **Table S1.** Continued |  | | |  | |  | |  | |  | |  |  |  |  |  |
| >SCSGFL1083D09 | | snrnp core sm protein sm-x5-like protein | 6 | | 6 | | 74.7124 | | NE-21 | | E-0 | | 4443.626 | 14633.8 | 4782.347 | 34153.27 |
| >SCACLR1036C04 | | pp2a regulatory subunit tap46 | 15 | | 11 | | 86.4801 | | E-0 | | NE-0 | | 6397.695 | 1251.437 | 4439.526 | 2941.933 |
| >SCJLST1022F12 | | n-ethylmaleimide sensitive fusion protein | 9 | | 3 | | 51.3135 | | NE-21 | | E-0 | | 2923.719 | 11565.36 | 2961.371 | 27516.53 |
| >SCJLRT1020E04 | | urate oxidase | 6 | | 5 | | 41.8733 | | NE-21 | | E-0 | | 3425.733 | 4887.648 | 3776.72 | 11703.28 |
| >SCEPLB1041A06 | | stad5_orysj | 8 | | 6 | | 46.189 | | NE-21 | | NE-0 | | 1267.809 | 667.0424 | 1534.473 | 1602.916 |
| >SCACSB1036C04 | | ruvbl1 protein | 28 | | 14 | | 178.2026 | | NE-21 | | NE-0 | | 24827.43 | 10252.01 | 21015.22 | 24903.52 |
| >SCACLR1036A01 | | h aca ribonucleoprotein complex subunit 2 | 7 | | 6 | | 45.3146 | | E-0 | | NE-0 | | 16144.75 | 1691.947 | 4487.39 | 4182.239 |
| >SCEQAD1018G06 | | ramosa 1 enhancer locus 2 | 29 | | 20 | | 215.6709 | | NE-21 | | NE-0 | | 24081.56 | 17986.74 | 25146.16 | 44636.39 |
| >SCJFRZ2025G05 | | chorismate mutase | 28 | | 14 | | 174.8319 | | NE-21 | | E-21 | | 19001.09 | 12094.21 | 4606.621 | 30110.01 |
| >SCSFAM1077D01 | | oxidoreductase [Zea mays] | 4 | | 2 | | 21.5302 | | NE-21 | | E-21 | | 755.3486 | 501.6291 | 82.62142 | 1262.165 |
| >SCCCRZ1004B05 | | topless-related protein 1-like | 26 | | 11 | | 161.7293 | | NE-21 | | NE-0 | | 8829.038 | 8449.516 | 15381.7 | 21321.71 |
| >SCJLLR1033G07 | | uba ubx kda protein | 15 | | 6 | | 84.003 | | NE-21 | | NE-0 | | 2268.045 | 1346.015 | 1875.489 | 3430.943 |
| >SCJFFL1C04C08.b | | 3-n-debenzoyl-2-deoxytaxol n-benzoyltransferase | 23 | | 14 | | 146.1507 | | NE-21 | | E-0 | | 7354.013 | 10140.09 | 9263.287 | 26259.26 |
| >SCSGLR1045A03 | | shikimate dehydrogenase1 | 30 | | 22 | | 190.7724 | | E-0 | | NE-0 | | 71224.86 | 14565.08 | 24695.19 | 37887.26 |
| >SCVPCL6046C06 | | Os04g0543900 [Oryza sativa Japonica Group] | 3 | | 2 | | 17.7088 | | NE-21 | | E-21 | | 1220.615 | 1415.225 | 100.06 | 3682.269 |
| >SCEZAM2059G06 | | ribosome recycling factor | 17 | | 9 | | 89.349 | | E-0 | | E-21 | | 17052.58 | 3938.213 | 3538.494 | 10269.45 |
| >SCEZRZ1012D01 | | rh37_orysj | 29 | | 9 | | 190.8626 | | NE-21 | | NE-0 | | 5115.331 | 3066.284 | 3948.095 | 8013.377 |
| >SCCCCL4015F12 | | xylanase inhibitor | 43 | | 23 | | 421.2061 | | NE-21 | | E-21 | | 139803 | 259774.9 | 82399.06 | 678950.7 |
| >SCRFAD1116E01 | | stem-specific protein tsjt1 | 34 | | 22 | | 276.8097 | | E-0 | | NE-0 | | 84224.95 | 23838.91 | 77818 | 62404.23 |
| >SCMCLV1031F09 | | invertase inhibitor-like | 8 | | 8 | | 70.4384 | | NE-21 | | E-0 | | 2163.731 | 27038.84 | 2723.95 | 71024.12 |
| >SCEPLB1043E09 | | rab gdp dissociation inhibitor alpha | 25 | | 12 | | 163.8801 | | E-21 | | NE-0 | | 3423.373 | 2676.743 | 7295.997 | 7032.843 |
| >SCEZLR1031B05 | | spfh domain band 7 family | 6 | | 3 | | 34.6506 | | NE-21 | | NE-0 | | 4345.645 | 2976.264 | 4382.28 | 7867.49 |
| >SCSGST1072B08 | | cytosol aminopeptidase | 16 | | 5 | | 136.9601 | | E-21 | | NE-0 | | 612.8977 | 389.3293 | 3859.101 | 1030.659 |
| >SCEPAM1015E02 | | d-3-phosphoglycerate dehydrogenase | 31 | | 25 | | 294.4057 | | NE-21 | | NE-0 | | 326750.2 | 187075.4 | 221275.1 | 498447.3 |
| >SCEQRT1030G05 | | ribose-phosphate pyrophosphokinase 4 | 14 | | 10 | | 118.0301 | | NE-21 | | NE-0 | | 26046.48 | 23973.2 | 27841.22 | 64399.63 |
| >SCCCRT1002E01 | | stad1_orysj | 8 | | 5 | | 46.97 | | NE-21 | | E-0 | | 3903.905 | 6875.601 | 5962.644 | 18702.35 |
| >SCJLST1027A10 | | thiamine thiazole synthase chloroplastic precursor | 9 | | 4 | | 56.0639 | | NE-21 | | E-21 | | 3055.331 | 7117.093 | 2284.071 | 19424.24 |
| **Table S1.** Continued |  | | |  | |  | |  | |  | |  |  |  |  |  |
| >SCCCCL4017B10 | | iws1 c-terminus family protein | 4 | | 3 | | 20.8175 | | NE-21 | | E-0 | | 489.9689 | 1540.352 | 1315.218 | 4221.65 |
| >SCMCCL6051B09 | | u3 small nucleolar rna-associated protein 25-like | 5 | | 4 | | 24.9419 | | NE-21 | | E-21 | | 11521.38 | 17671.76 | 1595.531 | 48995.25 |
| >SCJLRZ1027G11 | | in2-1 protein | 9 | | 7 | | 69.8633 | | E-21 | | NE-0 | | 12129.4 | 5725.117 | 23303.72 | 16061.56 |
| >SCCCCL7C03F06 | | isocitrate dehydrogenase | 32 | | 13 | | 244.3969 | | E-0 | | NE-0 | | 24403 | 6220.061 | 21570.44 | 17485.25 |
| >SCJFRT1010G02 | | dynamin-2b-like isoform 1 | 7 | | 5 | | 33.0535 | | E-21 | | NE-0 | | 6015.886 | 5999.79 | 18203.75 | 16942.4 |
| >SCCCCL4001H11 | | bgl15_orysj | 15 | | 11 | | 101.3812 | | E-21 | | NE-0 | | 3822.912 | 2200.807 | 6484.751 | 6230.069 |
| >SCVPRT2083C04 | | cue domain containing expressed | 3 | | 2 | | 15.2139 | | E-21 | | NE-0 | | 5441.777 | 1082.909 | 8172.5 | 3077.681 |
| >SCJLLR1101A05 | | dcp1-like decapping family protein | 7 | | 7 | | 42.4029 | | NE-21 | | E-0 | | 3716.453 | 5634.838 | 10559.9 | 16257.66 |
| >SCQSRT2034D09 | | carbonyl reductase 1 | 20 | | 13 | | 148.2089 | | E-0 | | NE-0 | | 29878.96 | 1672.689 | 23122.39 | 4949.219 |
| >SCCCCL3002B11.b | | alpha-amylase isozyme 3d precursor | 23 | | 15 | | 236.2604 | | E-0 | | NE-0 | | 154504.3 | 3261.995 | 136655.4 | 9655.833 |
| >SCBGSD2053C08 | | abscisic stress ripening protein 2 | 12 | | 7 | | 85.7047 | | NE-21 | | E-21 | | 5120.264 | 13486.04 | 1755.259 | 39963.07 |
| >SCJFAD1012C08 | | translation elongation factor 1 | 52 | | 11 | | 373.8943 | | NE-21 | | NE-0 | | 11383.29 | 4404.694 | 6885.204 | 13070.62 |
| >SCEPRZ1011H03 | | pyrroline 5-carboxylate reductase | 11 | | 7 | | 73.9084 | | NE-21 | | E-0 | | 3735.677 | 8580.364 | 4876.706 | 25741.32 |
| >SCJLAM1062B12 | | nadph-dependent reductase a1-a | 6 | | 4 | | 30.0011 | | E-0 | | NE-0 | | 22699.37 | 3426.701 | 20360.12 | 10341.15 |
| >SCCCLB1004B02 | | atpase subunit 1 | 11 | | 7 | | 105.8416 | | E-21 | | NE-0 | | 109311.1 | 29210.41 | 128329.5 | 88829.27 |
| >SCEPRZ1008B12 | | macronuclear development protein | 12 | | 8 | | 89.8305 | | NE-21 | | E-21 | | 2573.052 | 2932.062 | 2338.488 | 8922.866 |
| >SCVPRZ2037B11 | | dihydropyrimidine dehydrogenase | 19 | | 18 | | 159.4111 | | E-21 | | NE-0 | | 24026.93 | 14750.75 | 60567.56 | 45059.51 |
| >SCBGLR1113H05 | | farnesyl pyrophosphate synthetase | 12 | | 6 | | 91.2445 | | NE-21 | | E-21 | | 824.645 | 5621.449 | 422.5236 | 17235.47 |
| >SCSBAD1085A06 | | xylose isomerase | 35 | | 26 | | 332.9796 | | E-0 | | NE-0 | | 142849 | 39521.35 | 112764.8 | 123477.2 |
| >SCEQRZ3024B12 | | microtubule-associated protein | 28 | | 8 | | 160.1693 | | E-21 | | NE-0 | | 10088.79 | 1760.422 | 26434.63 | 5549.039 |
| >SCVPCL6061A06 | | plastid adp-glucose pyrophosphorylase large... | 10 | | 7 | | 55.5052 | | E-21 | | NE-0 | | 11303.03 | 988.4725 | 20576.22 | 3144.502 |
| >SCCCLR1070G05 | | mtbc_sorbi | 28 | | 17 | | 201.1421 | | E-21 | | NE-0 | | 28878.75 | 11935.25 | 140519.2 | 37973.03 |
| >SCQSRT3051B10 | | methylthioribose kinase | 10 | | 5 | | 62.65 | | E-21 | | NE-0 | | 2112.438 | 698.3932 | 2270.266 | 2229.916 |
| >SCCCRZ1003G02 | | pp1 pp2a phosphatases pleiotropic regulator prl1 | 5 | | 3 | | 26.3387 | | NE-21 | | NE-0 | | 1118.624 | 918.0021 | 1635.127 | 2971.032 |
| >SCCCCL4017A06 | | beta-glucosidase 31-like isoform 1 | 3 | | 2 | | 15.2858 | | NE-21 | | NE-0 | | 1678.982 | 1297.473 | 1329.622 | 4281.552 |
| >SCMCRZ3064B09 | | hemoglobin 2 | 14 | | 10 | | 121.1966 | | NE-21 | | E-0 | | 5347.02 | 22358.1 | 9900.894 | 73895.37 |
| >SCCCRT1002F07 | | pma1_orysj | 63 | | 40 | | 445.3845 | | NE-21 | | NE-0 | | 59287.03 | 25766.8 | 42766.4 | 89763.74 |
| **Table S1.** Continued |  | | |  | |  | |  | |  | |  |  |  |  |  |
| >SCRLFL4025E09 | | ribokinase-like isoform 1 | 9 | | 7 | | 54.8817 | | NE-21 | | NE-0 | | 4690.264 | 3783.085 | 6364.6 | 13283.89 |
| >SCCCRT1002C11 | | nicalin precursor | 17 | | 10 | | 96.7543 | | NE-21 | | E-21 | | 8836.812 | 4928.647 | 2762.683 | 17734.92 |
| >SCJFRT1008E10 | | af486280_1 cytosolic 6-phosphogluconate... | 24 | | 6 | | 193.7775 | | NE-21 | | E-0 | | 5332.047 | 6743.223 | 8151.91 | 25499.17 |
| >SCVPLR2027G04 | | peptidase family m48 containing protein | 4 | | 3 | | 22.5992 | | NE-21 | | NE-0 | | 1267.189 | 924.762 | 3063.853 | 3541.028 |
| >SCBFAD1094E08.b | | nadh dependent glutamate synthase | 85 | | 53 | | 604.6363 | | NE-21 | | E-21 | | 46421.4 | 53149.13 | 34266.18 | 204104.5 |
| >SCCCCL4002C03 | | letm1 and ef-hand domain-containing protein... | 24 | | 14 | | 147.6087 | | NE-21 | | NE-0 | | 13628.69 | 9902.282 | 16796.81 | 38696.67 |
| >SCSGST3118A01.b | | caffeic acid 3-o-methyltransferase | 20 | | 10 | | 137.8339 | | NE-21 | | E-21 | | 15412.92 | 7276.696 | 5117.86 | 28601.06 |
| >SCVPRT2078B04 | | myosin heavy chain-like protein | 6 | | 3 | | 29.1347 | | E-21 | | NE-0 | | 1552.039 | 484.2082 | 2948.918 | 1925.027 |
| >SCSBRZ2020D09 | | caffeoyl- o-methyltransferase-like | 16 | | 12 | | 100.8831 | | NE-21 | | E-0 | | 2891.371 | 18228.43 | 3587.817 | 74177.19 |
| >SCEQLR1092H02 | | oleosin 18 kda | 13 | | 12 | | 134.5861 | | E-21 | | NE-0 | | 18608.38 | 339.0747 | 120747 | 1386.569 |
| >SCCCCL3001A01.b | | 2-hydroxyphytanoyl- lyase | 14 | | 10 | | 88.0419 | | NE-21 | | E-0 | | 2777.797 | 11868.02 | 3355.886 | 48582.47 |
| >SCEQRT1024E12 | | phenylalanine ammonia-lyase | 56 | | 26 | | 360.1322 | | NE-21 | | E-21 | | 5448.114 | 27334.54 | 4022.891 | 113240.5 |
| >SCQGLR1086C06 | | kelch motif family protein | 29 | | 13 | | 198.6539 | | NE-21 | | NE-0 | | 14730.27 | 7704.591 | 10289.36 | 32421.39 |
| >SCCCCL5071H05 | | isovaleryl- dehydrogenase | 28 | | 14 | | 200.6749 | | NE-21 | | NE-0 | | 24172.15 | 5958.669 | 12368.21 | 25300.65 |
| >SCCCRZ2001C09 | | golgin candidate 5-like | 8 | | 4 | | 46.3676 | | NE-21 | | E-0 | | 151.301 | 820.8344 | 167.0511 | 3498.631 |
| >SCCCLR1067H01 | | mitogen activated protein kinase 20-1 | 8 | | 4 | | 39.9389 | | NE-21 | | NE-0 | | 2999.45 | 1033.968 | 2987.816 | 4652.819 |
| >SCACAD1037G05 | | substrate binding domain containing expressed | 12 | | 7 | | 81.5268 | | NE-21 | | E-0 | | 607.6821 | 5441.159 | 4157.033 | 24868.29 |
| >SCVPRT2075C09 | | c4-specific pyruvate orthophosphate dikinase | 30 | | 19 | | 187.1215 | | NE-21 | | NE-0 | | 45520.3 | 39157.38 | 55884.2 | 185790.6 |
| >SCAGLR2026C06 | | 4-methyl-5(b-hydroxyethyl)-thiazol monophosphate... | 21 | | 16 | | 168.0412 | | NE-21 | | NE-0 | | 16074.89 | 12031.5 | 16133.93 | 57486.78 |
| >SCRLLR1038A10 | | transaldolase 2 | 48 | | 29 | | 422.1533 | | NE-21 | | NE-0 | | 71264.32 | 47654.31 | 54510.37 | 229310.3 |
| >SCBFLR1083H09 | | pathogen induced protein 2-4 | 4 | | 4 | | 32.8852 | | NE-21 | | E-0 | | 2478.073 | 5277.258 | 3309.175 | 25524.87 |
| >SCCCLR1001H04 | | ubiquinol-cytochrome c reductase complex 14 kda... | 22 | | 10 | | 177.0342 | | E-21 | | NE-0 | | 14481.66 | 1877.655 | 19385.14 | 9144.241 |
| >SCEZRZ1016A05 | | casp c | 3 | | 2 | | 13.9554 | | NE-21 | | NE-0 | | 1141.956 | 327.5088 | 645.2625 | 1655.038 |
| >SCCCRT1003D07 | | b12d protein | 3 | | 2 | | 23.2545 | | E-21 | | NE-0 | | 93.18415 | 59.18682 | 1913.174 | 313.7282 |
| >SCQGLR2032B05 | | dehydrogenase reductase sdr family member 2 | 6 | | 3 | | 31.7552 | | E-0 | | NE-0 | | 9104.99 | 135.0033 | 6257.349 | 719.0342 |
| >SCQSLR1090A06 | | casein kinase ii alpha subunit | 10 | | 6 | | 70.769 | | NE-21 | | E-0 | | 3015.455 | 12954.44 | 3373.671 | 71110.41 |
| **Table S1.** Continued |  | | |  | |  | |  | |  | |  |  |  |  |  |
| >SCRUFL1111F06.b | | phospho-2-dehydro-3-deoxyheptonate aldolase 1 | 9 | | 3 | | 43.6486 | | NE-21 | | E-0 | | 564.6521 | 3012.684 | 1143.641 | 16561.47 |
| >SCCCLR1070A11 | | surfactant protein b containing protein | 7 | | 5 | | 52.1495 | | NE-21 | | E-0 | | 1176.12 | 3191.471 | 3484.528 | 18842.92 |
| >SCEZHR1087B05 | | homeobox-leucine zipper protein hox25-like | 1 | | 1 | | 5.2492 | | E-21 | | NE-0 | | 2049.79 | 305.1361 | 2468.555 | 1919.442 |
| >SCAGAD1076F02 | | ac074282_20 o-deacetylbaccatin... | 3 | | 2 | | 13.8464 | | E-21 | | NE-0 | | 2330.606 | 339.5244 | 3268.893 | 2222.181 |
| >SCCCCL7038F03 | | chloroplastic group iia intron splicing facilitator... | 3 | | 2 | | 15.0471 | | NE-21 | | E-0 | | 748.9879 | 1384.657 | 873.7133 | 9446.71 |
| >SCBFFL5072F08 | | kinase interacting protein 1 | 4 | | 2 | | 18.5957 | | E-21 | | E-0 | | 11.68252 | 92.78824 | 1394.916 | 644.0767 |
| >SCRLAM2050F05 | | domain containing expressed | 7 | | 4 | | 42.2633 | | NE-21 | | E-21 | | 498.2039 | 124.8057 | 111.0818 | 890.1537 |
| >SCCCCL7C03C05 | | af467541_1 aldehyde dehydrogenase mis1 | 20 | | 14 | | 158.9494 | | NE-21 | | NE-0 | | 31671.77 | 6804.664 | 48792.25 | 48851.5 |
| >SCJFRT1008A03.b | | wd repeat-containing protein 61-like | 19 | | 14 | | 119.7596 | | NE-21 | | NE-0 | | 73034.95 | 14837.62 | 26925.31 | 117662 |
| >SCACST3159E08 | | ferredoxin--nitrite reductase | 10 | | 8 | | 53.0397 | | NE-21 | | E-0 | | 2700.26 | 3154.465 | 3290.24 | 25180.65 |
| >SCBFRZ2047H07 | | receptor protein kinase clavata1 precursor | 17 | | 9 | | 105.8308 | | NE-21 | | E-0 | | 2476.152 | 7856.113 | 5605.902 | 63509.9 |
| >SCCCCL3080G02.b | | nucellin-like aspartic protease | 6 | | 4 | | 49.306 | | NE-21 | | E-0 | | 657.4146 | 1739.987 | 667.3672 | 14590.29 |
| >SCBGLR1095E10 | | blue copper protein precursor | 6 | | 2 | | 33.2082 | | NE-21 | | E-0 | | 3.966386 | 1374.824 | 24.96262 | 11616.93 |
| >SCAGLR1043B02 | | ap-2 complex subunit um | 9 | | 7 | | 44.1013 | | NE-21 | | E-21 | | 641.3112 | 1667.972 | 630.1002 | 14883.17 |
| >SCCCRZ2C04C12 | | inosine triphosphate pyrophosphatase | 3 | | 2 | | 22.7064 | | E-21 | | NE-0 | | 1794.463 | 112.9193 | 2557.42 | 1111.249 |
| >SCJFAD1011E09 | | arogenate dehydrogenase isoform 2 | 4 | | 3 | | 23.0113 | | NE-21 | | E-0 | | 38.8445 | 950.0637 | 96.9687 | 11675.79 |
| >SCCCLR1C04H07 | | fas-associated factor 1-like protein | 7 | | 5 | | 34.7869 | | NE-21 | | NE-0 | | 534.1301 | 399.9501 | 714.0567 | 5350.828 |
| >SCCCRZ2001F12 | | aluminum-induced protein | 12 | | 8 | | 67.4665 | | E-21 | | NE-0 | | 23395.65 | 2410.174 | 35225.23 | 32503.22 |
| >SCPRLB2030E07 | | mitogen-activated protein kinase-like | 8 | | 4 | | 44.1889 | | NE-21 | | NE-0 | | 620.0231 | 402.8649 | 889.1058 | 5829.69 |
| >SCAGLR1043H01 | | porphobilinogen deaminase | 10 | | 7 | | 72.3999 | | NE-21 | | NE-0 | | 1676.533 | 798.4983 | 2264.919 | 12047.12 |
| >SCCCLB1003B08 | | 5-enolpyruvylshikimate-3-phosphate partial | 5 | | 4 | | 39.0088 | | NE-21 | | E-21 | | 330.2331 | 490.2632 | 204.7612 | 7580.013 |
| >SCCCLB1003D11 | | exosome component 10-like | 2 | | 2 | | 10.5547 | | NE-21 | | NE-0 | | 3448.666 | 227.7669 | 3358.809 | 4598.659 |
| >SCBGLR1082E04 | | diphosphonucleotide phosphatase1 | 6 | | 4 | | 35.0161 | | NE-21 | | NE-0 | | 1526.339 | 377.1985 | 1391.945 | 7690.57 |
| >SCCCST3002H10 | | cysteine-type endopeptidase ubiquitin thiolesterase | 8 | | 2 | | 36.5644 | | NE-21 | | E-0 | | 13.73474 | 83.81224 | 92.6475 | 1710.596 |
| >SCAGFL8010F12 | | isco subunit binding-protein beta subunit | 28 | | 7 | | 192.5824 | | NE-21 | | NE-0 | | 1942.145 | 1389.551 | 2318.338 | 31924.4 |
| >SCCCCL7037E09 | | germin-like protein subfamily 1 member 17... | 3 | | 3 | | 25.9475 | | E-0 | | NE-0 | | 4731.655 | 126.189 | 388.9264 | 3606.88 |
| >SCSGFL4035A04 | | nefa-interacting nuclear protein | 4 | | 3 | | 21.8265 | | NE-21 | | NE-0 | | 921.2072 | 38.8317 | 608.9068 | 1187.849 |
| **Table S1.** Continued |  | | |  | |  | |  | |  | |  |  |  |  |  |
| >SCEQLR1007C02 | | adapter-related protein complex 1 beta 1 expressed | 6 | | 5 | | 30.9711 | | NE-21 | | NE-0 | | 1493.91 | 37.99793 | 1658.479 | 1954.023 |

1. Confidence scores are calculated by ProteinLynxGlobalServer (PLGS). They are indicated from biological repeat 1–3.
