# Supplementary material for: Label-Free Quantitative Proteomics of Embryogenic and Non-Embryogenic Callus during Sugarcane Somatic Embryogenesis
Source: PLoS One. 2015 Jun 2;10(6):e0127803. doi: 10.1371/journal.pone.0127803 (PMC4452777; doi:10.1371/journal.pone.0127803)
Supplement: S2 Table — (DOCX) [file pone.0127803.s004.docx]

**Table S2.** Non-exclusive proteins identified in the embryogenic and non-embryogenic sugarcane cultures submitted to maturation treatments.

| SUCEST accession number | Description | Peptide count | Peptides used for quantitation | Confidence score | Highest mean condition | Lowest mean condition | E-0 | NE-0 | E-21 | NE-21 |
| --- | --- | --- | --- | --- | --- | --- | --- | --- | --- | --- |
| **E-0 and E-21 co-expressed proteins** | |  |  |  |  |  |  |  |  |  |
| >SCCCCL7C05H06 | af377694_1globulin 1 | 3 | 1 | 31.3073 | E-21 | NE-0 | 119422.8 | - | 158714.7 | - |
| >SCRFLB1054G09 | hgd_orysj | 4 | 3 | 24.7468 | E-21 | NE-0 | 1078.17 | - | 24681.04 | - |
| >SCRUHR1074B04 | bet v i allergen | 4 | 3 | 23.4893 | E-21 | NE-0 | 546.4049 | - | 11045.67 | - |
| >SCSBRZ3124G06 | mutator protein | 2 | 1 | 9.921 | E-21 | NE-21 | 2366.436 | - | 11039.03 | - |
| >SCACCL6008G06 | moc31_maize | 1 | 1 | 4.6752 | E-21 | NE-0 | 14.22238 | - | 4936.53 | - |
| >SCCCLB1021E06 | granule bound starch synthase iia precursor | 5 | 3 | 33.8698 | E-0 | NE-0 | 96629.06 | - | 4618.632 | - |
| >SCSFAM1075G06 | nucleotide-binding protein 1 | 4 | 3 | 29.1558 | E-21 | NE-21 | 780.8832 | - | 4586.045 | - |
| >SCAGSD1041H08 | cleavage and polyadenylation specificity factor 5 | 5 | 3 | 29.7977 | E-21 | NE-21 | 772.5915 | - | 4284.757 | - |
| >SCQSRZ3037C07 | spl15_orysj | 2 | 1 | 15.4379 | E-21 | NE-21 | 2730.865 | - | 3168.567 | - |
| >SCBFRT1068E01 | ac093180_11 abc transporter | 3 | 2 | 16.8625 | E-21 | NE-21 | 528.3885 | - | 2881.488 | - |
| >SCCCLR1048F06 | phospholipid transfer protein 1 | 11 | 1 | 96.2497 | E-21 | NE-21 | 2200.105 | - | 2827.642 | - |
| >SCEZLB1005D03 | tld family expressed | 1 | 1 | 8.5722 | E-21 | NE-0 | 1310.173 | - | 2819.915 | - |
| >SCMCAM2081A09 | cepp protein | 1 | 1 | 5.3661 | E-0 | NE-21 | 6466.554 | - | 2717.662 | - |
| >SCMCCL6055G06 | math domain containing protein | 1 | 1 | 5.1637 | E-21 | NE-0 | 389.9162 | - | 2667.703 | - |
| >SCJLAM1062A05 | gp protein | 16 | 3 | 122.8607 | E-0 | NE-0 | 3032.642 | - | 1789.144 | - |
| >SCJLRZ1027C02 | truncated xa1-like protein | 4 | 2 | 23.7848 | E-21 | NE-21 | 1016.923 | - | 1630.003 | - |
| >SCQGLR2025G12 | nitrate-induced noi protein | 1 | 1 | 6.0061 | E-0 | NE-21 | 1732.745 | - | 1483.335 | - |
| >SCCCRZ2C02B02 | adaptor protein kanadaptin | 3 | 2 | 20.4898 | E-21 | NE-21 | 510.75 | - | 1453.147 | - |
| >SCJFLR1074D04 | 6pgl4_orysi short=6pgl 4 flags: precursor | 2 | 2 | 10.5112 | E-21 | NE-0 | 788.3677 | - | 1400.964 | - |
| >SCCCLR1048A07 | af083327_1101 kda heat shock protein | 3 | 2 | 24.7449 | E-21 | NE-21 | 991.7542 | - | 1327.934 | - |
| >SCCCLR1048B09 | alpha-expansin 5 | 3 | 1 | 19.7897 | E-21 | NE-0 | 220.1973 | - | 1176.967 | - |
| >SCEZFL5091D09 | adaptin n terminal region family protein | 5 | 1 | 36.2095 | E-21 | NE-0 | 601.8373 | - | 1167.83 | - |
| **Table S2.** Continued |  |  |  |  |  |  |  |  |  |  |
| >SCJFLR1013B09 | endonuclease exonuclease phosphatase family... | 8 | 1 | 42.4942 | E-0 | NE-21 | 1296.781 | - | 963.8132 | - |
| >SCRLAM1007E03 | ydg sra domain containing expressed | 3 | 1 | 18.68 | E-0 | NE-21 | 1320.854 | - | 910.6222 | - |
| >SCVPHR1090G07 | enhancer of rudimentary | 1 | 1 | 6.9601 | E-21 | NE-21 | 228.3095 | - | 871.0664 | - |
| >SCCCLR1001F08 | photosystem i reaction center subunit iii | 2 | 2 | 10.3432 | E-0 | NE-0 | 3278.126 | - | 866.8843 | - |
| >SCVPAM2068D11 | uridylate kinase | 3 | 2 | 19.0962 | E-21 | NE-0 | 410.3214 | - | 775.2716 | - |
| >SCQSST1037G12 | cdk5rap3-like protein | 6 | 2 | 29.3875 | E-0 | NE-21 | 913.9618 | - | 769.7857 | - |
| >SCMCFL5005E07 | fertilization-independent endosperm protein | 2 | 1 | 8.8596 | E-21 | NE-21 | 253.6779 | - | 765.5348 | - |
| >SCBFSD1038G02 | steroid 22-alpha-hydroxylase protein | 2 | 2 | 9.3773 | E-21 | NE-21 | 686.902 | - | 707.3843 | - |
| >SCACCL6010B02 | wrky transcription factor 4 | 6 | 2 | 29.7225 | E-21 | NE-21 | 540.7644 | - | 662.4369 | - |
| >SCBGFL4053H08 | ligatin (hepatocellular carcinoma-associated...) | 7 | 4 | 38.545 | E-0 | NE-0 | 2231.231 | - | 655.6858 | - |
| >SCSGFL4031D04 | duf246 domain-containing protein at1g04910-like | 1 | 1 | 5.287 | NE-21 | NE-0 | 573.3003 | - | 637.9043 | - |
| >SCEZRZ1015H08 | trehalose expressed | 1 | 1 | 5.315 | E-21 | NE-21 | 533.8582 | - | 549.0573 | - |
| >SCEQRT2093F11 | l-ascorbate peroxidase chloroplastic-like isoform1 | 2 | 1 | 13.1473 | E-0 | NE-0 | 2194.198 | - | 483.5013 | - |
| >SCCCLR1001A08 | negatively light-regulated protein | 3 | 1 | 17.8638 | E-21 | NE-21 | 46.65051 | - | 457.9018 | - |
| >SCJFRT1005C01 | 3-hydroxybutyryl- dehydrogenase | 2 | 1 | 11.6597 | NE-21 | NE-0 | 394.6509 | - | 433.4855 | - |
| >SCCCLR1080F11 | cycloartenol synthase | 6 | 2 | 31.1505 | E-0 | NE-21 | 960.6443 | - | 432.5893 | - |
| >SCEQRT2094F10 | scar-like protein 2-like | 2 | 1 | 9.0117 | E-21 | NE-0 | 99.73954 | - | 376.0142 | - |
| >SCRLAD1040A04 | ac104428_17 glutathione reductase | 1 | 1 | 5.8039 | E-21 | NE-21 | 217.0267 | - | 310.4601 | - |
| >SCEPLB1042B03 | heavy-metal-associated domain-containing... | 1 | 1 | 5.5208 | E-21 | NE-21 | 276.6267 | - | 310.2851 | - |
| >SCSFRT2071G10 | ubiquitin-specific protease | 9 | 1 | 56.174 | E-21 | NE-0 | 226.8296 | - | 302.0132 | - |
| >SCRUFL1019B10.b | ac090713_15 peroxidase | 4 | 1 | 22.7776 | E-0 | NE-21 | 285.5838 | - | 281.931 | - |
| >SCUTAM2010B06 | formin-like protein 16-like | 1 | 1 | 5.7904 | E-21 | NE-0 | 105.768 | - | 253.9452 | - |
| >SCUTST3086D12 | erebp-like protein | 2 | 1 | 11.1536 | E-0 | NE-21 | 339.3342 | - | 233.7392 | - |
| >SCQSRT1036B03 | lysine-ketoglutarate reductase saccharopine... | 3 | 1 | 15.7737 | E-21 | NE-0 | 110.9464 | - | 232.8247 | - |
| >SCEZLB1014A01 | saccharopine dehydrogenase | 2 | 1 | 10.128 | E-21 | NE-21 | 153.6449 | - | 230.0149 | - |
| >SCBGRT3071C03 | syntaxin-related protein nt-syr1 | 2 | 1 | 10.7001 | E-0 | NE-0 | 367.1587 | - | 193.2725 | - |
| >SCRFST1041F07 | map kinase activating | 2 | 1 | 11.3203 | E-0 | NE-0 | 218.3869 | - | 187.3048 | - |
| **Table S2.** Continued |  |  |  |  |  |  |  |  |  |  |
| >SCAGCL6016A03 | exocyst complex component expressed | 5 | 3 | 31.305 | E-0 | NE-21 | 10537.88 | - | 186.0253 | - |
| >SCJFRT2056F09 | tousled-like kinase 2 | 2 | 1 | 14.894 | E-0 | NE-21 | 149.2125 | - | 126.2326 | - |
| >SCRFRT3056B06 | fyve zinc finger family protein | 4 | 1 | 20.6945 | E-0 | NE-21 | 371.9014 | - | 122.9216 | - |
| >SCCCLB1003E11 | sapk3_orysi | 11 | 1 | 52.6467 | E-21 | NE-21 | 56.86367 | - | 121.2291 | - |
| >SCJFHR1034E06 | histidyl-trna synthetase | 2 | 1 | 10.605 | E-0 | NE-0 | 452.5816 | - | 115.9657 | - |
| >SCEQRT2095F07 | chitin-inducible gibberellin-responsive protein... | 2 | 1 | 15.2063 | E-21 | NE-21 | 31.31632 | - | 110.4365 | - |
| >SCEZLR1052A09 | vamp-like protein ykt62 | 4 | 2 | 21.126 | NE-21 | NE-0 | 44.02738 | - | 95.08381 | - |
| >SCEZRZ3049D03 | xylosyltransferase oxt | 2 | 1 | 11.4949 | E-0 | NE-21 | 230.1275 | - | 73.95792 | - |
| >SCJFLR1073H12 | delta 1-pyrroline-5-carboxylate synthetase | 7 | 1 | 38.2654 | E-0 | NE-21 | 874.9202 | - | 34.03878 | - |
| >SCEQRT1025A05 | ferritin- chloroplastic | 24 | 2 | 224.1018 | E-0 | NE-0 | 15105.71 | - | 10379.16 | - |
| >SCBFAM2023E05 | cohesin-like protein | 10 | 4 | 63.4802 | E-21 | NE-0 | 6482.564 | - | 15126.45 | - |
| >SCJFRZ2005H03 | cleft lip and palate transmembrane protein 1... | 7 | 4 | 34.4338 | E-21 | NE-0 | 7186.469 | - | 19755.17 | - |
| >SCCCCL3080B02.b | r-interacting factor1 | 15 | 9 | 98.6392 | E-21 | NE-21 | 8046.608 | - | 11324.67 | - |
| >SCBFRZ2019H02 | p-loop containing nucleoside triphosphate... | 3 | 3 | 21.2021 | E-21 | NE-0 | 742.373 | - | 13313.98 | - |
| >SCRLFL1005H09 | adenylyl cyclase-like | 4 | 3 | 19.2341 | E-21 | NE-0 | 5322.507 | - | 13257.39 | - |
| >SCBFLR1026E05 | vata_maize | 43 | 2 | 346.9665 | E-21 | NE-21 | 10230.56 | - | 12893.73 | - |
| >SCJFRT2053C06 | bola-like protein | 3 | 1 | 26.6587 | E-21 | NE-21 | 173.096 | - | 9946.539 | - |
| >SCQSAM2099E03 | Os12g0109600 [Oryza sativa Japonica Group] | 6 | 4 | 36.8272 | E-21 | NE-21 | 5754.243 | - | 8522.741 | - |
| >SCJLRT1023H02 | growth regulator | 5 | 2 | 28.8823 | E-0 | NE-21 | 12022.32 | - | 7059.053 | - |
| >SCAGFL8010G09 | skip interacting protein 18 | 11 | 3 | 63.4319 | E-0 | NE-0 | 8648.712 | - | 7036.335 | - |
| >SCEQAM1040A04 | cyclopropane fatty acid synthase | 7 | 2 | 41.6012 | E-21 | NE-0 | 3967.459 | - | 6886.839 | - |
| >SCEQRT1030A09 | aba stimulation map kinase | 7 | 3 | 46.1654 | E-0 | NE-0 | 7529.452 | - | 6850.633 | - |
| >SCCCCL3080B09.b | gh32_orysj | 3 | 2 | 22.2683 | E-0 | NE-21 | 6827.306 | - | 6181.195 | - |
| >SCAGLR1021D07 | acetohydroxyacid synthase | 15 | 10 | 80.9393 | E-0 | NE-21 | 11078.04 | - | 6132.397 | - |
| >SCJFLR1073H05 | ac079935_17 pyridoxamine 5-phosphate oxidase | 4 | 3 | 21.6584 | E-21 | NE-0 | 5675.852 | - | 5739.901 | - |
| >SCCCLR1076G12 | act7_orysi | 32 | 4 | 312.9281 | E-21 | NE-0 | 557.3014 | - | 5345.201 | - |
| >SCAGRT2042G05 | npl4 family protein | 9 | 6 | 65.6475 | E-0 | NE-0 | 11086.45 | - | 5336.08 | - |
| **Table S2.** Continued |  |  |  |  |  |  |  |  |  |  |
| >SCQSAM2100C06 | nudix hydrolase 13 | 7 | 2 | 43.6333 | E-0 | NE-21 | 4643.253 | - | 4577.331 | - |
| >SCEQRT1031A11 | 27k vesicle-associated membrane protein.... | 4 | 2 | 28.9022 | E-21 | NE-21 | 171.2609 | - | 4475.963 | - |
| >SCCCLR1024B12 | molybdenum cofactor biosynthesis protein | 4 | 3 | 27.2865 | E-21 | NE-0 | 2027.243 | - | 4111.339 | - |
| >SCCCRZ2001B06 | gtpase sar1 | 12 | 2 | 88.6145 | E-21 | NE-21 | 2112.971 | - | 3289.907 | - |
| >SCSGRT2062D07 | loc100280824 precursor | 6 | 3 | 48.4203 | E-0 | NE-21 | 5919.34 | - | 3465.834 | - |
| >SCRFAM1025D09 | sad1 unc-84-like protein 2 | 5 | 3 | 32.0012 | NE-21 | NE-0 | 1586.699 | - | 2522.812 | - |
| >SCJLLR2020C02 | pumilio-family rna-binding domain-containing... | 3 | 2 | 22.6798 | E-0 | NE-21 | 2628.643 | - | 2451.743 | - |
| >SCJFRZ2015B02 | myb family | 4 | 3 | 15.8657 | E-21 | NE-21 | 6.333296 | - | 2695.924 | - |
| >SCEZLR1009F04 | triose phosphate phosphate non-green precursor | 3 | 2 | 25.0663 | E-0 | NE-21 | 38914.99 | - | 3058.252 | - |
| >SCJLFL4186E12 | tocopherol cyclase | 2 | 1 | 10.8585 | NE-0 | E-21 | 2663.302 | - | 1489.258 | - |
| >SCJFRZ3C07F11 | amine expressed | 1 | 1 | 6.7596 | E-21 | NE-0 | 535.4959 | - | 1433.951 | - |
| >SCAGAM2126D12 | peptide-n4-(n-acetyl-beta-glucosaminyl)asparagine | 3 | 2 | 15.8159 | E-21 | NE-21 | 1227.892 | - | 1383.561 | - |
| >SCEZRZ3127F12 | phi-1 precursor | 7 | 3 | 57.2496 | E-21 | NE-0 | 465.9253 | - | 1652.396 | - |
| >SCSBRZ3125G04 | mechanosensitive ion channel domain-containing | 3 | 1 | 27.1658 | E-21 | NE-21 | 1357.818 | - | 1592.713 | - |
| >SCSBHR1052D02 | 12-oxo-phytodienoic acid reductase | 2 | 2 | 10.2798 | NE-0 | E-21 | 3174.634 | - | 1868.285 | - |
| >SCSGAD1009D06 | Os04g0136700 [Oryza sativa Japonica Group] | 4 | 1 | 21.5311 | E-21 | NE-0 | 865.6683 | - | 1900.441 | - |
| >SCQSST1038H12 | non-green plastid inner envelope membrane protein | 4 | 2 | 24.2926 | E-21 | NE-0 | 146.9131 | - | 2139.624 | - |
| >SCQSRT2036H09 | glycylpeptide n-tetradecanoyltransferase 1 | 3 | 2 | 24.1167 | E-21 | NE-21 | 1865.422 | - | 2122.651 | - |
| >SCCCRT1001F11 | decussate protein | 3 | 1 | 16.2406 | E-21 | NE-21 | 1300.464 | - | 1923.162 | - |
| >SCJLRT1006F02 | ngg1 interacting factor 3 like 1 binding protein 1... | 3 | 1 | 16.5657 | E-0 | NE-21 | 2297.967 | - | 1908.425 | - |
| >SCCCCL3004A05.b | ac079890_16 myb-related protein | 1 | 1 | 6.0122 | E-0 | NE-21 | 1950.133 | - | 1279.045 | - |
| >SCVPLR2027B10 | sm protein f | 4 | 1 | 24.4947 | E-21 | NE-0 | 1128.436 | - | 1247.712 | - |
| >SCRFLR1012H01 | 24 kda seed maturation protein | 2 | 1 | 15.5171 | E-21 | NE-21 | 544.5423 | - | 1247.681 | - |
| >SCRFLR1034A06 | upf0172 protein at5g55940-like | 5 | 3 | 27.047 | E-0 | NE-0 | 1396.791 | - | 1172.476 | - |
| >SCCCRT1003F05 | proline-rich cell wall | 5 | 2 | 25.4011 | E-21 | NE-0 | 420.9564 | - | 660.702 | - |
| >SCSGFL4194F09 | ocs-element binding factor | 2 | 1 | 12.7575 | E-21 | NE-21 | 233.7876 | - | 658.6958 | - |
| >SCSGLR1045F12 | rl11_orysj ame: full=60s ribosomal protein l11 | 12 | 2 | 95.7492 | E-0 | NE-21 | 1263.722 | - | 1056.037 | - |
| **Table S2.** Continued |  |  |  |  |  |  |  |  |  |  |
| >SCMCRT3084G08 | lactoylglutathione lyase | 8 | 3 | 53.6044 | E-0 | NE-21 | 1125.058 | - | 1007.641 | - |
| >SCSFAM1074E12 | abc-type transport system involved in resistance... | 6 | 3 | 40.9388 | NE-21 | NE-0 | 443.3123 | - | 989.4016 | - |
| >SCCCHR1003D08 | swirm domain-containing protein | 2 | 1 | 9.7118 | E-21 | NE-0 | 388.4356 | - | 906.4819 | - |
| >SCVPRT3090B11 | uvrb uvrc motif family protein | 3 | 2 | 13.8908 | E-0 | NE-0 | 995.5613 | - | 959.5507 | - |
| >SCSBAD1052A06 | fk506 binding protein | 1 | 1 | 4.6229 | E-0 | NE-0 | 872.1036 | - | 846.172 | - |
| >SCCCLB1004F04 | flowering time control protein isoform -4 | 4 | 3 | 26.8101 | NE-0 | E-0 | 453.2016 | - | 774.7984 | - |
| >SCACLR2007D05 | phloem-specific lectin | 1 | 1 | 6.3101 | E-0 | NE-0 | 1029.3 | - | 767.7769 | - |
| >SCVPRZ2038G11 | aaa-type atpase family expressed | 4 | 1 | 21.5782 | E-21 | NE-21 | 717.8112 | - | 737.1259 | - |
| >SCUTAM2088B01 | switch sucrose nonfermenting 3c | 2 | 2 | 9.8033 | E-0 | NE-0 | 1291.087 | - | 726.099 | - |
| >SCAGLR1021H04 | upf0510 protein inm02-like | 4 | 2 | 19.9272 | NE-21 | E-0 | 405.801 | - | 719.3362 | - |
| >SCCCRT1002A11 | neutral alpha-glucosidase ab expressed | 8 | 3 | 47.791 | E-21 | NE-21 | 537.7151 | - | 671.4131 | - |
| >SCEZRZ1013A06 | senescence-inducible chloroplast stay-green protein | 3 | 1 | 21.0673 | NE-0 | NE-21 | 393.2263 | - | 549.1498 | - |
| >SCBGSB1026A09 | bgl01_orysj | 1 | 1 | 5.2736 | E-21 | NE-21 | 310.0842 | - | 535.8566 | - |
| >SCJFRT1010D08 | 5-azacytidine resistance expressed | 2 | 2 | 10.9678 | NE-21 | E-0 | 148.9985 | - | 521.9716 | - |
| >SCBFRZ2046C02 | dual specificity protein phosphatase 4 | 2 | 1 | 10.8165 | E-0 | NE-21 | 1012.094 | - | 499.8202 | - |
| >SCJLLR1104B01 | p8mtcp1 | 2 | 1 | 16.2956 | NE-0 | E-0 | 249.1479 | - | 498.3417 | - |
| >SCBGLB2012C11 | polygalacturonase-1 non-catalytic beta subunit... | 2 | 1 | 9.8858 | E-0 | NE-21 | 310.1021 | - | 125.2583 | - |
| >SCEQRT2098A06 | phytochrome and flowering time protein 1 | 5 | 3 | 30.1907 | E-0 | NE-21 | 731.7702 | - | 349.6319 | - |
| >SCQSLR1090C03 | embryogenic callus protein 98b | 1 | 1 | 5.3158 | E-0 | NE-21 | 834.3746 | - | 347.4189 | - |
| >SCRLAM1007C02 | af272758_1kinesin heavy chain | 5 | 2 | 21.9783 | E-0 | NE-0 | 1038.233 | - | 434.9498 | - |
| >SCUTAD1032A02 | bzip transcription factor | 3 | 2 | 18.2784 | E-0 | NE-21 | 1190.952 | - | 429.0899 | - |
| >SCCCRT1002B01 | homocysteine s-methyltransferase 3 | 4 | 1 | 17.9407 | E-0 | NE-0 | 390.5908 | - | 339.2392 | - |
| >SCQGST1030G10 | gebp transcription factor | 3 | 1 | 18.7071 | E-21 | NE-21 | 126.6987 | - | 363.2258 | - |
| >SCRFLR1012F06 | sec61 alpha form 2 | 3 | 3 | 12.0126 | NE-21 | E-0 | 298.3154 | - | 359.4085 | - |
| >SCEQRT1028B04 | gck-like kinase mik | 2 | 1 | 8.8674 | E-21 | NE-21 | 123.7242 | - | 383.7148 | - |
| >SCAGAM2124H09 | parp1_maize ame: full=poly synthase 1 | 3 | 1 | 16.1601 | E-0 | NE-21 | 412.5143 | - | 326.4693 | - |
| >SCEZLR1031B12 | rh27_orysj | 5 | 4 | 24.0521 | E-0 | NE-21 | 3984.795 | - | 207.3643 | - |
| **Table S2.** Continued |  |  |  |  |  |  |  |  |  |  |
| >SCCCRT1001D03 | leaf senescence related | 1 | 1 | 4.7681 | E-21 | NE-21 | 182.0567 | - | 312.8176 | - |
| >SCJLHR1027G02 | anthocyanin biosynthetic gene regulator pac1 | 2 | 1 | 16.477 | E-21 | NE-0 | 169.2787 | - | 312.3001 | - |
| >SCQGLB2042B09 | wall-associated kinase 3 precursor | 1 | 1 | 6.1102 | NE-21 | E-0 | 149.3308 | - | 311.7912 | - |
| >SCRLFL1005D09 | clv1 receptor kinase-like protein | 1 | 1 | 5.2744 | NE-21 | E-0 | 47.30683 | - | 311.2656 | - |
| >SCACCL6007G01 | centromeric protein | 1 | 1 | 4.7387 | E-0 | NE-21 | 384.9289 | - | 294.6766 | - |
| >SCCCLR1C07B06 | gamma-glutamyl hydrolase precursor | 8 | 2 | 40.7686 | E-21 | NE-21 | 209.1849 | - | 285.2517 | - |
| >SCUTAM2086B11 | high light protein | 1 | 1 | 6.247 | NE-0 | E-21 | 487.2497 | - | 282.281 | - |
| >SCCCLR1048C10 | carbon-nitrogen family expressed | 11 | 6 | 57.2693 | NE-0 | E-21 | 163.7967 | - | 140.0126 | - |
| >SCRFLR1012H03 | ferritin 1a | 14 | 1 | 137.6533 | NE-21 | E-21 | 251.712 | - | 75.44348 | - |
| >SCCCCL3080E01 | loc100282003 precursor | 1 | 1 | 7.0442 | NE-0 | E-21 | 162.3203 | - | 73.05045 | - |
| >SCEPAM1017F02 | adenylate kinase | 4 | 2 | 24.3173 | E-0 | NE-21 | 639.6828 | - | 61.40506 | - |
| >SCRULR1042H01 | molecular chaperone | 2 | 2 | 8.7704 | E-0 | NE-0 | 831.6082 | - | 60.60552 | - |
| **E-0 and NE-0 co-expressed proteins** | |  |  |  |  |  |  |  |  |  |
| >SCCCRZ1001H10 | af466201_7small heat shock-like protein | 1 | 1 | 4.9336 | E-0 | E-21 | 1060.256 | 78.8719 | - | - |
| >SCBGSB1027G09 | psag protein | 4 | 1 | 16.5154 | E-0 | E-21 | 3559.846 | 86.76414 | - | - |
| >SCCCLR1067B10 | sec14 cytosolic factor | 3 | 1 | 20.1895 | E-0 | NE-21 | 923.3768 | 306.7306 | - | - |
| >SCRURT2009H10 | bsd domain containing protein | 3 | 1 | 20.2821 | E-0 | NE-21 | 130.4291 | 18.79472 | - | - |
| >SCBGLR1116G01 | cml13_orysj | 1 | 1 | 9.7526 | E-0 | NE-21 | 1876.712 | 340.4877 | - | - |
| >SCEQRT1030C03 | potassium transporter | 4 | 2 | 24.4503 | E-0 | E-21 | 674.8477 | 221.9122 | - | - |
| >SCQGLR1086F03 | ssxt protein | 3 | 2 | 18.813 | E-0 | NE-21 | 3981.076 | 90.89605 | - | - |
| >SCEZAM1080D04 | kinase-like protein | 1 | 1 | 5.2168 | NE-0 | NE-21 | 50.21461 | 1875.201 | - | - |
| >SCEZRZ3053H07 | latex-abundant protein | 3 | 1 | 14.388 | NE-0 | E-21 | 67.75435 | 118.516 | - | - |
| >SCMCFL5006A05 | cap protein | 2 | 2 | 11.2477 | NE-0 | NE-21 | 14.42866 | 1048.996 | - | - |
| >SCBFAD1048G12 | c3h27_orysj | 1 | 1 | 4.1728 | E-0 | NE-21 | 5317.621 | 130.6885 | - | - |
| >SCCCRT3004D06 | sex determination protein tasselseed 2 | 2 | 1 | 13.1896 | E-21 | NE-0 | 1738.776 | 158.5947 | - | - |
| >SCEQRT1028D11 | carnitine racemase catalytic racemase catalytic | 5 | 3 | 29.541 | NE-21 | E-0 | 2304.128 | 2669.127 | - | - |
| >SCEPAM2015B05 | af311223_1c2h2 zinc-finger protein | 3 | 2 | 21.9422 | NE-21 | E-0 | 2536.517 | 4734.287 | - | - |
| **Table S2.** Continued |  |  |  |  |  |  |  |  |  |  |
| >SCQGLR2025F03 | loc100285096 precursor | 1 | 1 | 5.2321 | NE-0 | E-0 | 729.1455 | 29637.08 | - | - |
| >SCAGCL6016A11 | insulin-degrading enzyme-like | 1 | 1 | 4.0948 | E-21 | NE-21 | 399.7043 | 340.9489 | - | - |
| >SCEZRZ1013F08 | diacylglycerol kinase | 2 | 1 | 17.5291 | NE-21 | E-0 | 524.8941 | 1434.854 | - | - |
| >SCJLLR1011E05 | plus-3 domain containing expressed | 2 | 2 | 9.5705 | NE-0 | E-0 | 159.9445 | 542.0793 | - | - |
| >SCEPLR1030D10 | developmentally-regulated gtp-binding protein 1 | 7 | 3 | 36.4096 | NE-0 | NE-21 | 3005.882 | 10652.65 | - | - |
| >SCJFRZ2007A04 | bet1 sft1-related snare | 3 | 1 | 22.2033 | E-0 | NE-21 | 1207.948 | 98.89431 | - | - |
| >SCSBFL1043F07 | phosphatase 2a regulatory a subunit | 10 | 1 | 68.0643 | NE-0 | E-21 | 23.6132 | 471.5577 | - | - |
| >SCCCCL3120D03 | gst6 protein | 4 | 2 | 24.7338 | E-0 | NE-21 | 2868.377 | 2797.104 | - | - |
| >SCJFRT1012D11 | poly(adp-ribose) polymerase | 3 | 1 | 16.4694 | E-0 | NE-21 | 2721.516 | 373.1395 | - | - |
| >SCSBFL4013A07 | geminivirus rep-interacting motor | 2 | 1 | 10.2771 | E-0 | NE-21 | 1915.896 | 162.7622 | - | - |
| >SCACSD2017E01 | chch domain containing protein | 2 | 2 | 17.9928 | E-0 | NE-0 | 4313.055 | 750.5825 | - | - |
| >SCVPLB1020E02 | zf-hd homeobox protein | 4 | 2 | 22.5077 | NE-0 | E-21 | 522.1445 | 851.797 | - | - |
| >SCBGLR1099D07 | zinc-binding protein | 4 | 2 | 27.9748 | E-0 | NE-21 | 1740.814 | 1029.407 | - | - |
| >SCCCCL3005F05.b | vernalization independence 4 | 1 | 1 | 7.5398 | NE-21 | E-21 | 916.0861 | 1292.813 | - | - |
| >SCJFRZ2025H09 | glutaredoxin i | 3 | 1 | 20.9448 | E-0 | NE-21 | 476.186 | 91.56269 | - | - |
| >SCEPRT2044F09 | bgal6_orysj | 2 | 2 | 11.4444 | E-0 | NE-0 | 774.9935 | 109.3112 | - | - |
| >SCCCLR1C08E10 | receptor-type tyrosine-protein phosphatase s | 3 | 1 | 15.2273 | NE-0 | E-21 | 97.08772 | 105.9372 | - | - |
| >SCJLRT2052B09 | caleosin related protein | 4 | 1 | 27.0221 | E-0 | NE-21 | 671.067 | 130.4102 | - | - |
| >SCVPRZ2041D10 | ring-h2 finger protein | 2 | 2 | 10.7752 | E-0 | E-21 | 10576.95 | 9128.581 | - | - |
| >SCRLAM1013H01 | beta-alanine synthase | 6 | 4 | 34.3283 | E-0 | NE-0 | 4207.603 | 201.0223 | - | - |
| >SCCCFL8001G12 | arsenical pump-driving atpase | 2 | 2 | 15.8619 | E-0 | NE-21 | 723.257 | 494.4283 | - | - |
| >SCEPLB1042D02 | nac2 protein | 6 | 3 | 30.7981 | E-0 | E-21 | 8396.966 | 2049.159 | - | - |
| >SCEQRT2099E04 | glucosyl transferase | 6 | 4 | 28.072 | NE-21 | E-21 | 244.8818 | 65.25165 | - | - |
| >SCMCAM2084F02 | hexokinase 5 | 2 | 1 | 10.7125 | E-21 | NE-0 | 429.4005 | 241.5855 | - | - |
| >SCEQRT2030H10 | auxin-inducible protein | 1 | 1 | 4.7787 | NE-0 | E-0 | 16.37097 | 1051.223 | - | - |
| **E-0 and NE-21 co-expressed proteins** | |  |  |  |  |  |  |  |  |  |
| >SCRFAM1025C02 | b3 domain-containing protein os03g0619600-like | 1 | 1 | 5.8706 | NE-21 | E-21 | 30.48963 | - | - | 2405.302 |
| **Table S2.** Continued |  |  |  |  |  |  |  |  |  |  |
| >SCMCSB1111D02 | rhamnogalacturonate lyase-like | 4 | 1 | 23.5721 | NE-21 | E-21 | 139.3517 | - | - | 4415.754 |
| >SCVPFL3044D11 | monoglyceride lipase-like | 2 | 2 | 11.0007 | NE-0 | E-21 | 59.00658 | - | - | 2199.115 |
| >SCVPLR2012H05 | tonneau expressed | 5 | 2 | 31.7575 | E-0 | E-21 | 877.7539 | - | - | 61.80625 |
| >SCEQLR1007G09 | single-strand binding protein | 3 | 1 | 15.3955 | E-21 | NE-0 | 98.16094 | - | - | 432.9607 |
| >SCEQRT2026E04 | glo3_orysj | 5 | 4 | 42.2544 | NE-21 | NE-0 | 89.19105 | - | - | 11860.73 |
| >SCEPLB1043G04 | cytokinin-o-glucosyltransferase 2 | 1 | 1 | 5.4354 | NE-21 | NE-0 | 97.89129 | - | - | 974.5534 |
| >SCSGRT2063D10 | silencing gene b101 | 6 | 4 | 36.7001 | E-21 | NE-0 | 720.8007 | - | - | 1096.76 |
| >SCCCCL6001A04 | dehydrin 11 | 4 | 3 | 34.0289 | E-21 | NE-0 | 42463.02 | - | - | 87.53858 |
| >SCRFLB1055D11 | mir-interacting saposin-like protein precursor | 4 | 2 | 21.8278 | NE-0 | E-21 | 2799.262 | - | - | 2233.506 |
| >SCSGRT2066E06 | basic helix-loop-helix family protein | 2 | 2 | 10.0949 | E-0 | NE-0 | 588.7743 | - | - | 71.62245 |
| >SCQSRT2033B03 | single myb histone 1 | 2 | 2 | 7.7643 | E-0 | NE-0 | 181.9722 | - | - | 63.4017 |
| >SCBFST3133F03 | rab1 small gtp-binding protein | 4 | 1 | 31.0843 | NE-0 | E-21 | 62.57188 | - | - | 82.56396 |
| **E-21 and NE-21 co-expressed proteins** | |  |  |  |  |  |  |  |  |  |
| >SCQSRT2032G07 | atp-binding cassette sub-family e member expressed | 7 | 2 | 43.7112 | NE-21 | E-0 | - | - | 1542.181 | 1826.774 |
| >SCACLR2007G05 | remorin-like isoform 1 | 5 | 2 | 25.2142 | E-21 | NE-0 | - | - | 676.0972 | 165.2924 |
| >SCRFFL1025G01 | formate--tetrahydrofolate ligase-like | 6 | 3 | 33.8444 | E-21 | E-0 | - | - | 2190.808 | 149.0613 |
| **NE-0 and NE-21 co-expressed proteins** | |  |  |  |  |  |  |  |  |  |
| >SCCCCL2001A04.b | xylem serine proteinase 1 precursor | 4 | 1 | 18.2139 | NE-0 | E-0 | - | 597.346 | - | 206.8833 |
| >SCCCRZ1C01C03 | nonsense-mediated mrna decay protein 3 | 5 | 1 | 29.6395 | NE-0 | E-21 | - | 1646.653 | - | 1602.347 |
| >SCCCLR1048A10 | 60 ribosomal protein l14 | 10 | 1 | 85.762 | NE-0 | E-21 | - | 1702.804 | - | 1659.839 |
| >SCRLSB1042H02 | ribonucleoside-diphosphate reductase | 2 | 1 | 8.389 | NE-21 | E-21 | - | 200.1354 | - | 3142.556 |
| >SCJLRT1006C03 | acc synthase | 3 | 2 | 13.296 | E-0 | NE-21 | - | 687.4143 | - | 236.6315 |
| >SCEQRT2026F02 | smc2 protein | 3 | 2 | 18.836 | NE-21 | E-21 | - | 294.1504 | - | 2249.753 |
| **NE-0 and E-21 co-expressed proteins** | |  |  |  |  |  |  |  |  |  |
| >SCUTST3129G12 | gdp-l-fucose synthase 1 | 5 | 1 | 35.2344 | E-21 | NE-21 | - | 294.6131 | 5158.496 | - |
| >SCCCLR2003G12 | brefeldin a-inhibited guanine nucleotide-exchange... | 1 | 1 | 5.5506 | E-21 | E-0 | - | 1277.32 | 1493.317 | - |
| >SCMCRT2104E04 | glycerophosphodiester phosphodiesterase | 3 | 1 | 15.7586 | NE-0 | E-0 | - | 4236.655 | 608.6302 | - |
| **Table S2.** Continued |  |  |  |  |  |  |  |  |  |  |
| >SCSGFL4C06C03 | dna-damage-repair toleration protein | 3 | 2 | 15.7275 | NE-0 | E-0 | - | 2462.8 | 51.25466 | - |
| **E-0. E-21 and NE-21 co-expressed proteins** | |  |  |  |  |  |  |  |  |  |
| >SCVPRT2080F11 | 3-methyl-2-oxobutanoate hydroxymethyltransferase | 6 | 5 | 33.6337 | E-21 | NE-0 | 1761.78 | - | 3010.027 | 77.1727 |
| >SCCCCL7C02A05 | peroxin pex14 | 5 | 2 | 25.1365 | E-21 | NE-0 | 711.2938 | - | 1223.035 | 393.6839 |
| >SCCCCL4C01F06 | atp12 atpase | 4 | 1 | 20.3682 | NE-21 | NE-0 | 1319.603 | - | 990.3515 | 1986.301 |
| >SCSFST3076G09 | luminal binding protein | 13 | 1 | 118.626 | E-0 | NE-0 | 1116.049 | - | 929.9285 | 33.81498 |
| >SCJLRT1018D04 | roothairless 1 | 3 | 2 | 15.4031 | E-21 | NE-0 | 155.162 | - | 837.2004 | 27.37189 |
| >SCVPFL1072G12 | gamma-tubulin complex component 3 homolog | 1 | 1 | 6.2997 | E-21 | NE-0 | 247.4116 | - | 606.4626 | 51.91938 |
| >SCCCCL7C02F04 | dpe1_orysj | 5 | 1 | 24.3169 | E-0 | NE-0 | 903.6117 | - | 8.754731 | 74.74067 |
| >SCCCLR1024C05 | nonspecific lipid-transfer protein 3 precursor | 2 | 2 | 15.8891 | NE-21 | E-21 | 118.4203 | - | 72.65892 | 4332.72 |
| >SCQSRZ3037B01.b | minor isoform | 7 | 2 | 77.7615 | NE-21 | E-0 | 54.97563 | - | 58.2645 | 7694.211 |
| >SCSBST3101G12 | multiple c2 and transmembrane domain-containing... | 1 | 1 | 4.8189 | NE-21 | E-21 | 95.43805 | - | 90.65438 | 2987.049 |
| >SCRURT2012B06 | lysine ketoglutarate reductase saccharopine... | 6 | 3 | 30.5248 | E-0 | E-21 | 5117.682 | - | 84.28483 | 3192.181 |
| >SCEPAM1016H05 | early-responsive to dehydration stress protein | 2 | 1 | 10.7201 | NE-21 | NE-0 | 47.06252 | - | 32.82346 | 520.6783 |
| >SCCCLR1C07E12 | ccr4-not transcription complex subunit 7 | 1 | 1 | 9.7556 | NE-21 | E-21 | 55.3322 | - | 25.6008 | 22310.87 |
| >SCJFRZ2013G11 | metk4_horvu | 8 | 1 | 94.0577 | NE-21 | E-21 | 449.8119 | - | 19.97597 | 4465.023 |
| >SCBFAM2023E02 | homoserine kinase | 1 | 1 | 7.7797 | E-21 | NE-0 | 315.0325 | - | 2099.533 | 68.76865 |
| >SCCCRT2004D12 | snare domain containing protein | 6 | 3 | 44.2215 | E-21 | NE-0 | 813.9506 | - | 1904.813 | 1455.977 |
| >SCBGLR1119F12 | gldh1_orysj | 9 | 5 | 65.5153 | NE-21 | NE-0 | 510.0832 | - | 1883.551 | 4230.875 |
| >SCCCLR1024G08 | catalytic acting on nadh or nadph | 14 | 7 | 75.3387 | NE-21 | NE-0 | 1446.179 | - | 1857.036 | 24559.93 |
| >SCJLFL4097H07.b | 2-keto-3-deoxy-l-rhamnonate aldolase-like | 2 | 2 | 11.9587 | E-21 | NE-0 | 989.9905 | - | 1719.915 | 422.434 |
| >SCRUFL4020G01 | inositol monophosphatase 3 | 3 | 3 | 24.4356 | E-0 | NE-0 | 7733.469 | - | 1845.878 | 483.18 |
| >SCCCCL5071H07 | calcyclin-binding protein | 5 | 2 | 27.0673 | NE-21 | NE-0 | 2158.102 | - | 2957.128 | 3568.668 |
| >SCJLFL3015D04 | degp1 | 3 | 2 | 23.4407 | E-21 | NE-0 | 3428.293 | - | 14991.13 | 571.4653 |
| >SCCCCL3002C12.b | reticulon-like protein b2-like isoform 1 | 3 | 1 | 33.9967 | E-21 | NE-0 | 11528.89 | - | 11664.51 | 3517.665 |
| >SCCCRT3010C11 | amidohydrolase family protein | 4 | 3 | 25.2055 | E-21 | NE-21 | 5278.618 | - | 12061.74 | 244.8793 |
| >SCSBSD1033E10 | per1_sorbi | 9 | 7 | 75.9006 | E-21 | NE-0 | 1820.812 | - | 21628.64 | 396.7859 |
| **Table S2.** Continued |  |  |  |  |  |  |  |  |  |  |
| >SCRLLB2032D09 | flower-specific gamma-thionin precursor | 3 | 3 | 44.0915 | E-21 | NE-0 | 4384.83 | - | 32454.95 | 276.7213 |
| >SCRFLR2037D07 | nad h-dependent oxidoreductase | 7 | 3 | 57.5961 | E-0 | NE-21 | 12629.37 | - | 7182.559 | 387.2621 |
| >SCMCAM2084B09 | flavonoid 3 -hydroxylase | 12 | 4 | 66.6351 | NE-0 | NE-21 | 6115.678 | - | 6081.802 | 5103.755 |
| >SCCCAM2001F04 | auxin-repressed kda protein | 5 | 2 | 43.6788 | E-21 | NE-0 | 3923.346 | - | 4153.277 | 839.3202 |
| >SCCCAM2001F04 | ubiquinone biosynthesis protein coq9 | 2 | 2 | 19.0502 | E-21 | NE-0 | 1360.777 | - | 3196.04 | 1724.915 |
| >SCEZAM2031E09 | dut_orysj | 7 | 5 | 58.5372 | E-0 | NE-0 | 5109.524 | - | 3718.156 | 110.7801 |
| >SCVPFL3045D08 | hemoglobin-like protein | 3 | 3 | 15.8265 | E-21 | NE-21 | 1968.437 | - | 4875.157 | 820.0707 |
| >SCCCLR1C03B10 | Os01g0663800 [Oryza sativa Japonica Group] | 8 | 2 | 55.7002 | E-21 | NE-0 | 2286.275 | - | 5511.444 | 119.9844 |
| >SCVPLR2005B12 | mct-1 like pua rna binding-domain containing... | 8 | 6 | 53.3802 | E-0 | NE-0 | 10515.2 | - | 5749.859 | 3731.864 |
| >SCACCL6007H09 | fd vi | 9 | 6 | 57.4914 | E-0 | E-21 | 25061.01 | - | 2265.134 | 5722.617 |
| >SCVPAM2067E05 | fmn binding protein | 1 | 1 | 10.4592 | E-0 | NE-0 | 5503.53 | - | 2128.56 | 1258.772 |
| >SCACLR1036G09 | sucrose-phosphate synthase | 3 | 3 | 15.2631 | E-21 | E-0 | 135.9911 | - | 1025.502 | 387.0763 |
| >SCEQRT1031B11 | acyl-coenzyme a oxidase 2 | 15 | 7 | 83.2302 | E-0 | E-21 | 6411.549 | - | 1175.045 | 3626.623 |
| >SCJFST1015B05 | altered response to gravity | 2 | 2 | 14.9227 | E-0 | NE-21 | 1284.938 | - | 1199.719 | 871.6458 |
| >SCJLRT2051F02 | utp--glucose-1-phosphate uridylyltransferase-like | 16 | 6 | 132.9731 | NE-21 | NE-0 | 2319.989 | - | 1194.516 | 8818.51 |
| >SCJLRZ1024F09 | phosphoribosyl pyrophosphate synthase | 2 | 1 | 10.3874 | E-21 | E-0 | 368.6241 | - | 648.1967 | 416.075 |
| >SCRFSD2022E02 | apospory-associated protein c | 8 | 5 | 53.3628 | E-0 | NE-0 | 1518.211 | - | 1309.02 | 863.6151 |
| >SCSGAM1096C06 | insulinase containing expressed | 4 | 3 | 19.8139 | E-21 | NE-0 | 299.3052 | - | 533.0246 | 430.57 |
| >SCJFRZ2032C10 | dek protein | 6 | 3 | 37.214 | NE-21 | NE-0 | 794.4643 | - | 292.9086 | 8476.367 |
| >SCUTLR1037C03 | loc100282421 precursor | 3 | 2 | 18.2353 | NE-21 | E-0 | 87.07206 | - | 200.8873 | 5265.112 |
| >SCVPLR2005B02 | thylakoid lumenal kda chloroplast precursor | 3 | 2 | 15.259 | NE-0 | E-21 | 216.7141 | - | 198.5861 | 2526.918 |
| >SCQSLB2054E09 | gnat family protein | 4 | 1 | 19.5209 | E-0 | NE-21 | 567.4801 | - | 340.4747 | 155.1932 |
| >SCMCSB1107H02 | resistance protein rga2 | 2 | 1 | 9.8558 | NE-21 | E-0 | 40.10382 | - | 339.8106 | 47435.72 |
| >SCEQLB1063H10 | iaa21_orysj | 1 | 1 | 5.1469 | NE-21 | E-0 | 142.9205 | - | 476.9637 | 1163.111 |
| >SCEPLR1051C10 | ef-hand containing protein | 3 | 3 | 17.535 | E-21 | NE-0 | 197.5069 | - | 443.8401 | 290.0542 |
| >SCQSAD1059D02 | kinesin heavy chain | 9 | 6 | 41.5309 | NE-21 | NE-0 | 681.9785 | - | 442.3909 | 1943.035 |
| **E-0. NE-0 and E-21 co-expressed proteins** | |  |  |  |  |  |  |  |  |  |
| **Table S2.** Continued |  |  |  |  |  |  |  |  |  |  |
| >SCCCAM2004E12 | plastidic phosphate translocator-like protein1 | 4 | 3 | 18.1097 | E-0 | NE-21 | 15774.13 | 3858.449 | 2912.492 | - |
| >SCBGST3104H11 | 23 kda polypeptide of photosystem ii | 1 | 1 | 5.6361 | E-0 | NE-21 | 1051.793 | 355.9346 | 336.227 | - |
| >SCEZFL5087C02 | double strand break repair protein | 3 | 2 | 16.8577 | E-21 | NE-21 | 316.7647 | 9.06048 | 463.3046 | - |
| >SCCCRZ2002B03 | hydroxyproline-rich glycoprotein 1 | 9 | 1 | 73.8111 | E-0 | NE-21 | 64.28073 | 62.18255 | 40.53833 | - |
| >SCCCRZ1C01D10 | unnamed protein product [Oryza sativa Japonica...] | 7 | 2 | 42.653 | E-0 | E-21 | 7134.456 | 265.2607 | 53.59017 | - |
| >SCEZLR1031G01 | udp-arabinose 4-epimerase 2 | 5 | 2 | 27.6552 | E-0 | E-21 | 76735.26 | 153.3325 | 12.05067 | - |
| >SCACLB1048B09 | alpha-mannosidase calcium ion binding protein | 2 | 1 | 10.4472 | NE-21 | E-21 | 43.69594 | 82.67582 | 11.41805 | - |
| >SCACAD1037F10 | exb18_orysj | 4 | 3 | 23.7802 | NE-0 | E-21 | 138.148 | 8018.908 | 9.093619 | - |
| >SCAGLR1021H06 | vignain precursor | 8 | 4 | 61.456 | NE-0 | E-21 | 394.2885 | 7305.515 | 230.2136 | - |
| >SCVPAM1055E12 | selt selw selh selenoprotein domain containing... | 7 | 4 | 35.527 | E-0 | NE-21 | 802.3868 | 72.00119 | 284.8517 | - |
| >SCSGFL1081H07 | sad1-unc84-like protein | 7 | 4 | 33.3651 | NE-0 | E-21 | 191.0601 | 3428.673 | 128.8825 | - |
| >SCQGAM1049G02 | starch branching enzyme interacting protein-1 | 8 | 4 | 47.7523 | NE-0 | E-21 | 2151.239 | 7902.248 | 306.6329 | - |
| >SCUTST3092C06 | epoxide hydrolase 2-like | 8 | 6 | 47.2257 | E-0 | E-21 | 35158.14 | 2694.353 | 333.6142 | - |
| >SCQGST1034H10 | will die slowly expressed | 4 | 3 | 26.6666 | E-0 | E-21 | 1839.576 | 558.5942 | 418.769 | - |
| >SCUTRZ3106H10 | formylglycineamide ribotide amidotransferase | 10 | 5 | 68.5696 | NE-0 | E-21 | 744.0202 | 2344.876 | 470.7005 | - |
| >SCCCCL3140E08 | phosphoribosylformylglycinamidine cyclo-... | 4 | 2 | 31.6582 | NE-0 | E-21 | 1560.223 | 6223.431 | 429.5291 | - |
| >SCJFHR1031E10 | 50s ribosomal protein l12-1 | 9 | 4 | 46.5522 | NE-0 | E-0 | 71.22124 | 938.3691 | 718.7987 | - |
| >SCRUAD1132B11 | methylcytosine binding domain protein | 1 | 1 | 4.5156 | NE-21 | E-0 | 82.76021 | 583.608 | 103.9999 | - |
| >SCSGRT2065E10 | loc100282067 precursor | 3 | 2 | 19.9744 | NE-0 | NE-21 | 326.6184 | 5893.124 | 590.3282 | - |
| >SCRLAD1044E07 | l-galactose dehydrogenase | 1 | 1 | 6.8887 | E-0 | NE-21 | 766.9356 | 464.3744 | 588.204 | - |
| >SCRFAM2131E11 | phytoene synthase | 2 | 2 | 9.4491 | NE-21 | NE-0 | 732.2367 | 388.723 | 541.5807 | - |
| >SCACLR2029D05 | loc100285305 precursor | 3 | 2 | 21.7758 | NE-21 | E-0 | 109.2857 | 2573.115 | 534.3879 | - |
| >SCJLLR1105H10 | mp703_orysj | 2 | 2 | 8.1709 | NE-0 | E-0 | 501.2447 | 3640.307 | 517.0848 | - |
| >SCQGLB1039F03 | nodulation receptor kinase | 3 | 3 | 14.6472 | E-0 | NE-21 | 2000.654 | 1341.734 | 514.0626 | - |
| >SCCCRZ1001A11 | sec20 family protein | 3 | 2 | 20.8764 | NE-0 | NE-21 | 1395.914 | 22902.74 | 931.6277 | - |
| >SCEZLR1009E04 | eyes absent-like protein | 4 | 3 | 27.4525 | E-21 | NE-0 | 876.1701 | 15.54965 | 1176.04 | - |
| >SCRFAM2128E12 | sl-tps p | 3 | 2 | 16.8636 | E-21 | NE-0 | 96.65735 | 20.90383 | 1133.738 | - |
| **Table S2.** Continued |  |  |  |  |  |  |  |  |  |  |
| >SCQGAM1048E11 | ac079936_18 retroelement | 5 | 4 | 23.422 | E-0 | E-21 | 4672.923 | 2253.799 | 1000.622 | - |
| >SCCCRZ1001C07 | hap5 subunit of hap complex | 4 | 3 | 32.5117 | NE-0 | NE-21 | 1610.378 | 2849.615 | 623.6084 | - |
| >SCPIRT3022D04 | ac091238_14 cytosolic trna-ala synthetase | 13 | 2 | 86.5601 | NE-21 | E-0 | 135.0246 | 1074.185 | 953.3261 | - |
| >SCCCCL4013E03 | Oryza sativa unknown protein AAP03423... | 6 | 1 | 31.5819 | E-21 | E-0 | 125.188 | 551.5151 | 815.3719 | - |
| >SCMCST1055F01 | proliferating-cell nucleolar | 5 | 2 | 23.4391 | NE-0 | NE-21 | 963.3151 | 1050.656 | 777.4044 | - |
| >SCQSLB1051H10 | agenet domain containing expressed | 4 | 2 | 21.5879 | NE-0 | NE-21 | 1477.208 | 1634.64 | 755.4896 | - |
| >SCRFLR1012H05 | act1_orysj | 27 | 2 | 306.9723 | NE-0 | E-21 | 936.2848 | 2328.022 | 645.0663 | - |
| >SCJLRT3077C07 | sec1 family transport protein sly1-like | 7 | 1 | 36.2007 | E-0 | NE-21 | 1144.292 | 258.3949 | 640.4691 | - |
| >SCJLRT2049H04 | chorimate mutase | 2 | 2 | 9.5971 | NE-21 | E-0 | 1255.577 | 3891.668 | 1382.872 | - |
| >SCCCRZ1003H11 | mitochondria fission 1 protein | 6 | 3 | 34.1163 | E-0 | E-21 | 1315.439 | 1109.502 | 806.429 | - |
| >SCUTLR1058B12 | thymidylate kinase | 7 | 2 | 39.7275 | NE-0 | E-0 | 946.7576 | 1520.814 | 1311.927 | - |
| >SCJFRT1008C02 | retinol dehydrogenase 14 | 5 | 3 | 27.1081 | NE-0 | E-0 | 1706.215 | 6877.955 | 2033.654 | - |
| >SCEQRT1030C08 | translin [Zea mays] | 3 | 2 | 17.6202 | E-21 | NE-21 | 993.7517 | 398.1878 | 1981.164 | - |
| >SCBFRT1064C08 | iron ascorbate-dependent oxidoreductase | 10 | 4 | 76.1614 | NE-0 | E-0 | 1030.091 | 23383.08 | 1955.328 | - |
| >SCQGLR1019E12 | fha domain containing protein | 2 | 1 | 9.7062 | E-0 | NE-21 | 3641.748 | 1805.511 | 1925.347 | - |
| >SCEPRZ3045G05 | leucine-rich repeat receptor protein kinase exs | 14 | 4 | 79.1813 | NE-21 | NE-0 | 1589.139 | 319.3423 | 1745.326 | - |
| >SCCCST1006G04 | translin-associated protein x-like isoform 1 | 9 | 6 | 64.0795 | E-21 | NE-0 | 1355.846 | 521.3984 | 1848.783 | - |
| >SCQSRT2035A05 | infection-related protein | 4 | 3 | 37.0237 | NE-21 | E-0 | 1199.147 | 2145.877 | 1839.595 | - |
| >SCSFRT2072E04 | 4-hydroxyphenylpyruvate dioxygenase | 1 | 1 | 6.5948 | E-21 | NE-0 | 1581.854 | 823.1841 | 1665.959 | - |
| >SCCCAM2001A07 | pht42_orysj | 1 | 1 | 4.5075 | NE-0 | NE-21 | 655.922 | 4298.64 | 1649.644 | - |
| >SCCCCL2001A02.b | pepper esterase | 8 | 4 | 45.1032 | NE-0 | E-21 | 2370.735 | 10389.39 | 1526.71 | - |
| >SCEZRT3070D05 | mdr-like abc transporter | 4 | 2 | 21.1741 | E-0 | E-21 | 3428.655 | 2065.567 | 1465.477 | - |
| >SCVPCL6046A05 | copg1_orysj | 16 | 6 | 87.7479 | NE-21 | E-21 | 1632.75 | 2256.318 | 1430.982 | - |
| >SCVPRZ2038A09 | cob21_orysj | 6 | 4 | 37.3246 | E-0 | E-21 | 7837.044 | 6688.87 | 3020.179 | - |
| >SCQSRT2033C03 | ethanol tolerance protein geko1 | 3 | 1 | 20.3816 | E-21 | E-0 | 123.2549 | 125.1517 | 2919.365 | - |
| >SCCCRZ2001D05 | pre-mrna branch site p14-like protein | 3 | 3 | 28.6008 | E-21 | NE-0 | 2384.574 | 320.6915 | 2794.994 | - |
| >SCEPLB1044D11 | plant-specific domain tigr01615 family protein | 4 | 2 | 32.9506 | E-0 | E-21 | 8932.957 | 3606.297 | 2734.669 | - |
| **Table S2.** Continued |  |  |  |  |  |  |  |  |  |  |
| >SCCCRT1001G02 | versicolorin reductase | 6 | 2 | 33.2725 | E-0 | NE-21 | 5907.252 | 764.9383 | 3040.588 | - |
| >SCCCCL4001D12 | cyanate hydratase | 2 | 2 | 21.9722 | E-0 | NE-0 | 3520.175 | 1230.268 | 2658.649 | - |
| >SCCCST3144G03 | metal-dependent hydrolase-like protein | 8 | 6 | 63.2076 | NE-21 | NE-0 | 2905.149 | 584.8807 | 2551.911 | - |
| >SCCCRZ2004A04 | Os07g0150500 [Oryza sativa Japonica Group] | 5 | 4 | 23.9921 | E-21 | NE-21 | 1907.725 | 1059.21 | 2480.417 | - |
| >SCRUAD1132E05 | phytosulfokine receptor precursor | 2 | 2 | 9.2182 | E-21 | NE-21 | 2615.969 | 1287.666 | 3403.838 | - |
| >SCQGFL4079G07 | wd-40 repeat protein | 7 | 4 | 47.1281 | E-21 | NE-21 | 1953.717 | 105.6473 | 3127.854 | - |
| >SCRLRT3035G03 | ubitquitin c-terminal hydrolase | 9 | 3 | 54.4231 | NE-0 | NE-21 | 2755.972 | 31065.53 | 3119.615 | - |
| >SCCCCL3120G05.b | structure-specific recognition protein 1 | 5 | 4 | 25.8091 | E-0 | NE-21 | 4740.21 | 3887.497 | 3107.151 | - |
| >SCEZHR1084H04 | pollen-specific protein like | 4 | 2 | 38.15 | E-0 | NE-21 | 11533.86 | 3937.401 | 5343.229 | - |
| >SCQSRT2036A02 | pp2ac-3 - phosphatase 2a isoform 3 belonging... | 10 | 2 | 66.2043 | NE-21 | E-0 | 4669.505 | 6230.793 | 5340.275 | - |
| >SCEZLB1007D07 | polyphenol oxidase | 3 | 3 | 15.5869 | E-21 | NE-0 | 3012.103 | 792.4447 | 4794.5 | - |
| >SCEPAM2057G04 | dpod1_orysj ame: full=dna polymerase delta... | 2 | 2 | 10.6328 | E-0 | E-21 | 14747.64 | 6864.06 | 5015.448 | - |
| >SCVPCL6042B11 | salt-induced map kinase 1 | 11 | 6 | 64.2107 | E-21 | NE-21 | 1729.685 | 1026.303 | 4405.632 | - |
| >SCJLLR1104B12 | erwinia induced protein 1 | 1 | 1 | 7.5513 | E-0 | NE-21 | 8923.51 | 1944.728 | 3978.774 | - |
| >SCEZSB1090C11 | temperature-induced lipocalin-1 | 7 | 5 | 42.0165 | E-0 | NE-0 | 5708.457 | 602.8452 | 3955.759 | - |
| >SCBGSB1027H10 | soul heme-binding | 4 | 3 | 24.11 | E-21 | NE-0 | 2771.342 | 1243.865 | 3942.296 | - |
| >SCRFRZ3058E08 | external nadh-ubiquinone oxidoreductase... | 6 | 4 | 34.4928 | E-21 | E-0 | 2153.085 | 2430.45 | 3784.615 | - |
| >SCRLLR1111B02 | sam domain family protein | 10 | 5 | 64.7544 | E-0 | NE-21 | 5544.678 | 3792.77 | 3784.12 | - |
| >SCCCRT3009E06 | pre-mrna-processing atp-dependent rna helicase... | 10 | 4 | 58.0686 | E-0 | NE-0 | 6508.331 | 1039.53 | 3767.172 | - |
| >SCCCRZ1004B02 | ribonuclease h-related protein | 7 | 3 | 42.8958 | E-0 | NE-21 | 4946.254 | 2343.388 | 3660.463 | - |
| >SCRUSB1064E09 | cysp2_maize ame: full=cysteine proteinase 2 flags... | 6 | 3 | 47.9438 | NE-0 | E-21 | 7898.981 | 17869.75 | 6812.689 | - |
| >SCCCRZ1002G03 | act3_orysj ame: full=actin-3 | 33 | 2 | 344.3913 | E-0 | NE-21 | 8766.081 | 7300.621 | 6500.15 | - |
| >SCUTRZ3102C06 | phi-1-like phosphate-induced protein | 5 | 3 | 30.6385 | E-21 | NE-21 | 255.0773 | 18.28845 | 6448.763 | - |
| >SCRFRZ3057C09 | peptidoglycan-binding domain-containing | 3 | 3 | 22.375 | E-0 | NE-21 | 16684.2 | 2302.737 | 6431.682 | - |
| >SCEQLR1050H09 | drepp4 protein | 4 | 4 | 24.9143 | NE-0 | E-0 | 182.6463 | 9108.036 | 5844.083 | - |
| >SCEZLB1005C03 | tankyrase 1 | 6 | 2 | 33.3212 | E-21 | NE-0 | 374.6888 | 122.7598 | 5577.494 | - |
| >SCEZLR1009H06 | af215854_1hexose partial | 2 | 2 | 15.3778 | E-21 | NE-0 | 2358.403 | 55.32743 | 6867.49 | - |
| **Table S2.** Continued |  |  |  |  |  |  |  |  |  |  |
| >SCRFLB2059H06 | adp-glucose pyrophosphorylase small subunit | 22 | 12 | 144.9706 | E-0 | NE-21 | 10311.04 | 5065.598 | 7510.95 | - |
| >SCJLLR1108E06 | carbonic anhydrase | 7 | 4 | 49.5694 | NE-0 | E-21 | 12322.08 | 131555 | 7607.723 | - |
| >SCCCCL3001E04.b | non- nadp-malic enzyme | 8 | 3 | 49.1138 | E-0 | NE-0 | 11231.75 | 3670.582 | 7800.023 | - |
| >SCEPAM1108G11 | quaking isoform 5-like | 2 | 2 | 9.7339 | NE-0 | NE-21 | 4615.568 | 19602.34 | 8171.536 | - |
| >SCCCLR1077A10 | glutamyl-trna cytoplasmic | 9 | 6 | 57.1029 | E-21 | NE-0 | 1859.967 | 222.5148 | 8164.008 | - |
| >SCCCCL4012E07 | s-glutathione dehydrogenase class iii alcohol... | 14 | 4 | 109.5344 | E-21 | NE-21 | 6561.471 | 1333.066 | 8389.695 | - |
| >SCBGAM1090F06 | soluble inorganic pyrophosphatase | 11 | 6 | 82.1282 | E-0 | NE-21 | 15369.3 | 11799.69 | 8360.348 | - |
| >SCJFST1010F06 | nadph adrenodoxin oxidoreductase | 16 | 8 | 89.2106 | E-0 | NE-21 | 13603.5 | 380.2137 | 10603.94 | - |
| >SCSBFL1039B03 | dead h (asp-glu-ala-asp his) box polypeptide 8... | 2 | 1 | 11.1072 | NE-0 | NE-21 | 14624.58 | 23514.9 | 9636.499 | - |
| >SCJFLR1073E04 | strictosidine synthase precursor | 16 | 11 | 121.4546 | NE-0 | NE-21 | 4691.139 | 23539.87 | 9543.327 | - |
| >SCCCCL7038E07 | importin-beta n-terminal domain containing... | 10 | 7 | 69.512 | E-0 | NE-21 | 23223.14 | 10652.52 | 12029.34 | - |
| >SCAGLR1043A01 | yt521-b-like family protein | 4 | 2 | 29.1814 | E-21 | NE-21 | 12968.8 | 25011.41 | 37673.99 | - |
| >SCCCCL4012H11 | abc transporter f family member 3-like | 4 | 4 | 18.6233 | E-21 | NE-0 | 1944.476 | 440.4467 | 21238.46 | - |
| >SCCCLR2004H12 | ago1b_orysj | 29 | 4 | 169.1564 | E-21 | NE-21 | 9155.702 | 902.4522 | 23064.23 | - |
| >SCEQRZ3093C12 | ubiquitin-fold modifier 1 precursor | 3 | 2 | 24.4975 | E-21 | NE-21 | 2807.161 | 110.4378 | 16938.21 | - |
| >SCJFHR1C04H10.b | importin-alpha re-exporter | 6 | 6 | 42.7684 | E-21 | NE-21 | 6458.499 | 11190.28 | 15183.97 | - |
| >SCJLFL4100F04 | ferredoxin- chloroplastic-like | 4 | 2 | 35.6062 | E-0 | NE-0 | 29156.78 | 2232.372 | 15992.6 | - |
| >SCQGLB1039E05 | er6 protein | 6 | 3 | 53.6673 | E-0 | NE-0 | 12890.14 | 3735.439 | 10923.46 | - |
| >SCSGAD1005H05 | cullin 3 | 10 | 3 | 47.4942 | E-21 | NE-21 | 11670.8 | 2460.572 | 12344.64 | - |
| >SCEPAM1019D01 | nap16kda protein | 3 | 3 | 40.3968 | E-21 | NE-21 | 2428.835 | 817.1337 | 11219.64 | - |
| >SCCCCL4015D08 | e chain localization of the small subunit ribosomal... | 14 | 1 | 112.8181 | E-0 | NE-0 | 15671.35 | 11459.66 | 15235.81 | - |
| **E-0. NE-0 and NE-21 co-expressed proteins** | |  |  |  |  |  |  |  |  |  |
| >SCCCLB1001B05 | chitin elicitor-binding | 4 | 2 | 21.6771 | NE-21 | E-21 | 420.8737 | 6033.358 | - | 10116.82 |
| >SCJLFL1049E05 | clathrin-adaptor medium chain apm 4 | 10 | 4 | 58.9873 | NE-0 | E-0 | 78.62876 | 7125.762 | - | 6009.186 |
| >SCSBSB1053C11 | acc1_orysj ame: full=acetyl- carboxylase 1 includes | 1 | 1 | 4.3334 | NE-21 | E-0 | 114.3596 | 4268.581 | - | 4733.753 |
| >SCQSLR1040B10 | palmitoyl-protein thioesterase 1 precursor | 3 | 2 | 15.9526 | E-21 | E-0 | 344.386 | 422.7714 | - | 540.4591 |
| >SCBFLB2091F04 | flavonol sulfotransferase-like | 3 | 1 | 19.7916 | E-21 | NE-21 | 1166.721 | 517.0104 | - | 182.0259 |
| **Table S2.** Continued |  |  |  |  |  |  |  |  |  |  |
| >SCQSRT1035D12 | permatin precursor | 4 | 2 | 26.0562 | NE-0 | E-0 | 137.1969 | 4886.809 | - | 380.3357 |
| >SCCCCL4003D04 | isoleucyl-trna cytoplasmic-like | 12 | 5 | 67.6126 | NE-0 | E-0 | 4248.496 | 32897.05 | - | 23400.72 |
| >SCVPCL6042C08 | 1-deoxy-d-xylulose 5-phosphate reductoisomerase | 9 | 6 | 60.6001 | E-21 | NE-0 | 34304.79 | 4007.443 | - | 5205.608 |
| >SCJFRZ2013G10 | structural maintenance of chromosomes protein6... | 14 | 6 | 73.1554 | NE-21 | E-0 | 1278.765 | 4152.011 | - | 9250.292 |
| >SCCCLR1C02A06 | glo5_orysj | 3 | 1 | 17.5293 | NE-21 | NE-0 | 481.1488 | 151.9026 | - | 824.6285 |
| >SCSBAM1086G07 | zll pnh homologous protein | 8 | 1 | 41.2647 | E-0 | NE-0 | 541.8879 | 282.5288 | - | 412.4079 |
| >SCCCLR1066H03 | hydrogen-transporting atp rotational mechanism | 5 | 3 | 29.2021 | NE-21 | E-21 | 10412.86 | 18186.37 | - | 18335.99 |
| >SCCCCL4010H07 | mitotic checkpoint protein bub3 | 3 | 2 | 14.8223 | NE-21 | NE-0 | 1585.449 | 408.9103 | - | 2086.659 |
| >SCQSLB1052F09 | 10-deacetylbaccatin iii 10-o-acetyltransferase | 6 | 2 | 34.3282 | E-0 | NE-0 | 4607.275 | 1854.447 | - | 2368.631 |
| >SCCCLR1C05G11 | ring3 protein | 4 | 2 | 18.9807 | NE-0 | E-21 | 3324.157 | 9430.254 | - | 9051.915 |
| >SCCCLR1024B04 | vesicle-associated membrane protein 727 | 8 | 3 | 52.2444 | NE-0 | E-21 | 868.2298 | 2932.157 | - | 1219.637 |
| >SCCCLR1069D05 | actin- expressed | 36 | 1 | 386.7377 | NE-21 | NE-0 | 1786.629 | 1131.222 | - | 1820.509 |
| >SCEPLR1030A12 | inner envelope membrane protein | 4 | 3 | 28.3519 | NE-0 | E-21 | 1697.231 | 4256.233 | - | 2214.452 |
| >SCSFAM1075C07 | inositol-tetrakisphosphate 1-kinase 1 | 5 | 2 | 26.5442 | NE-21 | E-21 | 3592.448 | 5124.949 | - | 9023.039 |
| >SCQSRT1037E09 | nifu-like protein mitochondrial-like | 4 | 4 | 23.2077 | NE-21 | E-21 | 958.711 | 3212.499 | - | 3763.386 |
| >SCJFRT1059E08 | pto kinase interactor 1 | 11 | 4 | 70.7686 | E-0 | NE-21 | 5630.7 | 4074.264 | - | 1143.92 |
| >SCQGLR1062G06 | ferredoxin-dependent glutamate chloroplast... | 4 | 3 | 21.421 | NE-21 | E-21 | 257.6783 | 1181.762 | - | 6378.519 |
| >SCRFFL4009A01 | pp2ac-5 - phosphatase 2a isoform 5 belonging... | 19 | 3 | 117.212 | NE-21 | NE-0 | 1315.987 | 177.3273 | - | 2251.268 |
| >SCCCCL4009H01 | trans-cinnamate 4-monooxygenase | 4 | 2 | 18.8515 | E-0 | E-21 | 993.84 | 590.7497 | - | 703.3816 |
| >SCEQRT1026G06 | phosphoglucomutase chloroplast | 8 | 5 | 60.2844 | NE-21 | E-21 | 7917.716 | 4436.402 | - | 8722.87 |
| >SCACAD1037A09 | proton translocating pyrophosphatase | 21 | 3 | 141.4369 | NE-21 | E-21 | 1212.704 | 830.4915 | - | 3702.382 |
| >SCCCCL4002G01 | ctp synthase | 2 | 2 | 11.6762 | NE-0 | NE-21 | 1133.711 | 1938.844 | - | 68.32218 |
| >SCCCLR2C02D12 | chlorophyll a b-binding apoprotein cp26 precursor | 1 | 1 | 5.5556 | E-0 | E-21 | 6436.806 | 4318.837 | - | 1835.235 |
| >SCCCLR1022C06 | aci-reductone dioxygenase-like protein | 8 | 4 | 48.9687 | E-0 | E-21 | 11585.7 | 5988.313 | - | 3129.438 |
| >SCEQRT1033E11 | 2-nitropropane dioxygenase-like protein | 5 | 2 | 23.5674 | NE-21 | E-21 | 69.12966 | 2547.253 | - | 3060.996 |
| >SCEPRT2043A01 | mannitol dehydrogenase | 11 | 4 | 56.5515 | NE-0 | E-21 | 935.5621 | 2827.667 | - | 198.7454 |
| >SCACFL5027G07 | ac079179_18 retroelement | 1 | 1 | 6.3214 | NE-0 | E-21 | 743.9192 | 10095.22 | - | 6153.512 |
| **Table S2.** Continued |  |  |  |  |  |  |  |  |  |  |
| >SCCCRT2002D03 | type-1 pathogenesis-related protein | 2 | 1 | 13.8263 | NE-21 | NE-0 | 924.5275 | 62.41272 | - | 4775.178 |
| >SCACAM2043E01 | phenazine biosynthesis protein | 4 | 2 | 22.8644 | E-0 | E-21 | 9559.566 | 2208.471 | - | 723.995 |
| >SCRFLR2037A10 | type 1 membrane | 6 | 4 | 38.65 | NE-21 | E-21 | 448.33 | 514.1831 | - | 24574.63 |
| >SCCCCL4002F09 | snf2 transcription factor | 5 | 1 | 24.8291 | NE-0 | E-21 | 1099.163 | 2222.095 | - | 1174.269 |
| >SCCCRT2C09A10 | phosphoglucose isomerase | 10 | 1 | 79.4738 | NE-0 | E-21 | 198.1708 | 954.03 | - | 614.9566 |
| >SCQSRT2033E01 | nicotinate phosphoribosyltransferase-like protein | 5 | 2 | 29.3151 | NE-21 | E-21 | 72.96752 | 1114.423 | - | 4862.998 |
| **NE-0. E-21 and NE-21 co-expressed proteins** | |  |  |  |  |  |  |  |  |  |
| >SCJFAD1010G05.b | alpha-l-arabinofuranosidase c-terminus family... | 1 | 1 | 6.4773 | NE-0 | E-0 | - | 3298.045 | 120.2074 | 2617.588 |
| >SCQSLR1061D05 | ac068923_15 pre-mrna splicing factor | 3 | 1 | 16.5192 | E-21 | NE-0 | - | 69.07862 | 1514.217 | 733.1552 |
| >SCSGLR1025F04 | galactose kinase | 2 | 2 | 10.6786 | E-21 | E-0 | - | 4065.088 | 5396.657 | 898.1771 |
| >SCQGST3154G02 | nfyb4_orysj | 3 | 3 | 16.6891 | E-0 | NE-21 | - | 561.5512 | 479.0187 | 65.23303 |
| >SCQGLR2032A01 | copine i-like | 1 | 1 | 3.486 | NE-0 | E-0 | - | 8299.828 | 435.3948 | 6873.041 |

1. Confidence scores are calculated by ProteinLynxGlobalServer (PLGS). They are indicated from biological repeat 1–3.
